# Direct Observation of Membrane-Associated H-Ras in the Native Cellular Environment by In-Cell ^19^F-NMR Spectroscopy

**DOI:** 10.1021/jacsau.3c00108

**Published:** 2023-06-01

**Authors:** Masaomi Ikari, Hiromasa Yagi, Takuma Kasai, Kohsuke Inomata, Masahiro Ito, Kae Higuchi, Natsuko Matsuda, Yutaka Ito, Takanori Kigawa

**Affiliations:** † RIKEN Center for Biosystems Dynamics Research, Kanagawa 230-0045, Japan; ‡ PRESTO/Japan Science and Technology Agency, Saitama 332-0012, Japan; § SI Innovation Center, Taiyo Nippon Sanso Corporation, Tokyo 206-0001, Japan; ∥ Department of Chemistry, Graduate School of Science, Tokyo Metropolitan University, Tokyo 192-0397, Japan

**Keywords:** small GTPase, site-specific ^19^F-labeling, membrane-trafficking, exogenous protein delivery, Bayesian spectral deconvolution, unnatural amino acid incorporation

## Abstract

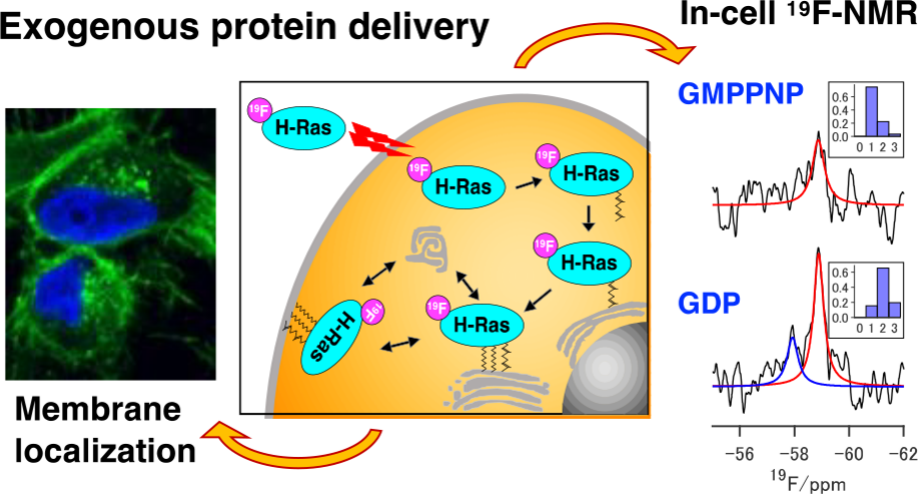

Ras acts as a molecular switch to control intracellular signaling on the plasma membrane (PM). Elucidating how Ras associates with PM in the native cellular environment is crucial for understanding its control mechanism. Here, we used in-cell nuclear magnetic resonance (NMR) spectroscopy combined with site-specific ^19^F-labeling to explore the membrane-associated states of H-Ras in living cells. The site-specific incorporation of *p*-trifluoromethoxyphenylalanine (OCF_3_Phe) at three different sites of H-Ras, i.e., Tyr32 in switch I, Tyr96 interacting with switch II, and Tyr157 on helix α5, allowed the characterization of their conformational states depending on the nucleotide-bound states and an oncogenic mutational state. Exogenously delivered ^19^F-labeled H-Ras protein containing a C-terminal hypervariable region was assimilated via endogenous membrane-trafficking, enabling proper association with the cell membrane compartments. Despite poor sensitivity of the in-cell NMR spectra of membrane-associated H-Ras, the Bayesian spectral deconvolution identified distinct signal components on three ^19^F-labeled sites, thus offering the conformational multiplicity of H-Ras on the PM. Our study may be helpful in elucidating the atomic-scale picture of membrane-associated proteins in living cells.

## Introduction

**Figure 1:**
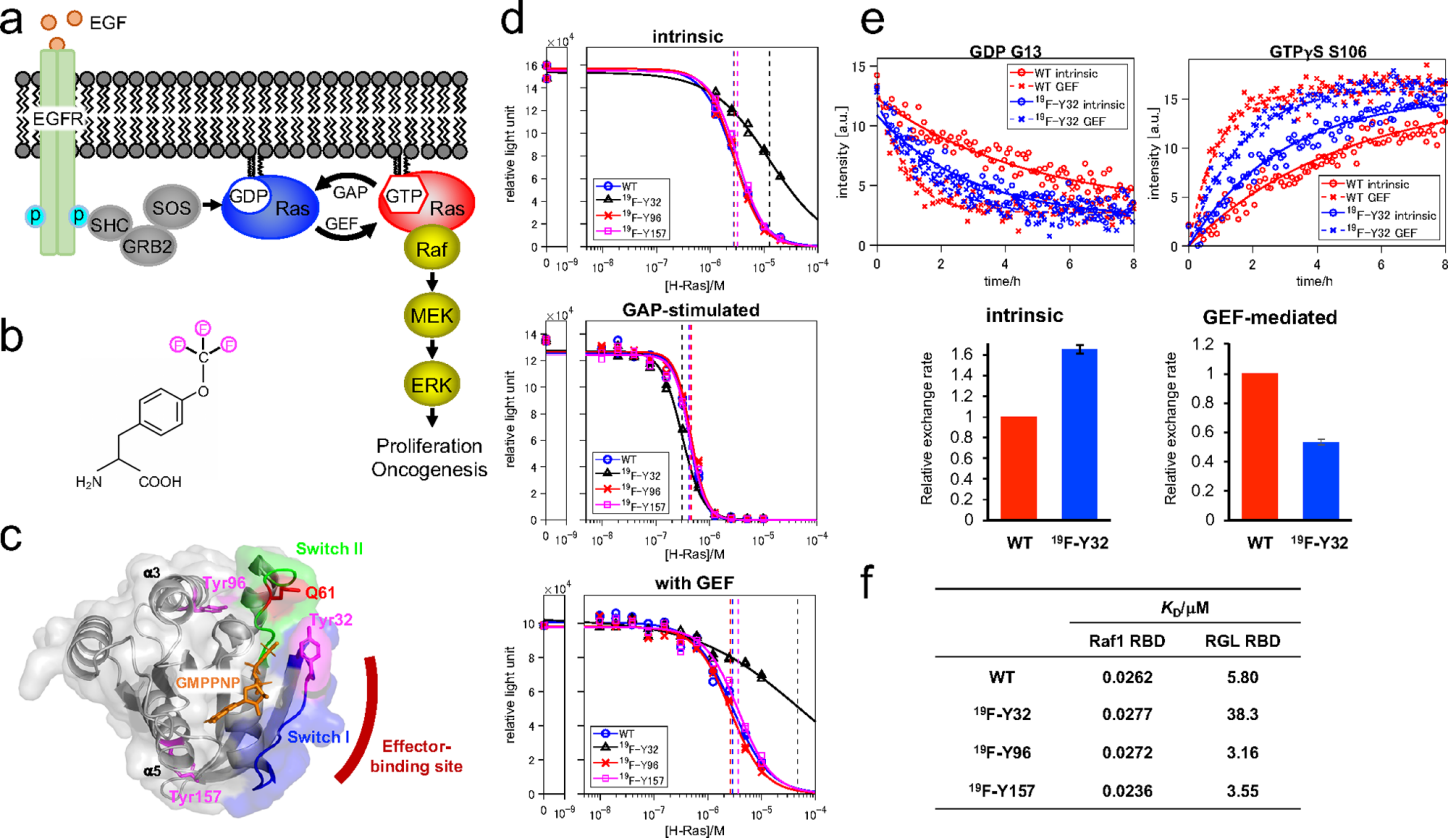
In vitro characterization of the ^19^F-labeled H-Ras. (a) Schematic illustration of Ras signaling on the plasma membrane. (b) Structure of *p*-trifluoromethoxyphenylalanine (OCF_3_Phe). (c) Overall protein architecture of H-Ras in this study. The positions of Tyr32, Tyr96, Tyr157, Gln61, switch I, and switch II are shown on the crystal structure of GMPPNP-bound H-Ras (PDB code: 5P21). The effector-binding site is also indicated. (d) GTP hydrolysis activity measurement using the Promega GTPase-Glo assay system. The data represent an average of three independent experiments. Dotted lines indicate the EC_50_ value of each H-Ras protein (also see Figure S4). (e) Intrinsic and GEF-mediated nucleotide-exchange (NE) activities of the H-Ras WT and ^19^F-Y32 H-Ras WT. (Top) Real-time NMR-derived NE curves. The peak intensities of G13 (GDP form) and S106 (GTPγS form) versus time are representatively plotted. The continuous and dotted lines represent fitting of the equations (see eqs S7 or S8 in the Supporting Information) to the experimental data. (Bottom) The intrinsic and GEF-mediated relative NE rates of the H-Ras WT and ^19^F-Y32 H-Ras WT. (f) Binding affinities of the H-Ras WT and ^19^F-labeled H-Ras WT proteins toward Raf1 RBD and RGL RBD.

Ras acts as a molecular switch to control multiple intracellular signal-transduction pathways (Figure 1a).^1^ Similar to many small GTPases, Ras is tethered to the cell membrane via its C-terminal hypervariable region (HVR), which undergoes multiple post-translational modifications and processing through membrane-trafficking.^2^ The interaction of Ras with the plasma membrane (PM) is essential for its activation, downstream signaling, and several switch mechanisms.^3,4^ Oncogenic mutations cause continuous Ras activation, promoting cancer progression; hence, inhibiting pathological Ras activation is a potential anticancer therapeutic target. Recent advancements in technology have allowed us to investigate the association of Ras with membranes. For example, NMR spectroscopy using paramagnetic relaxation enhancement (PRE) has demonstrated nucleotide-dependent reorientations of the globular domain (G-domain) of K-Ras4B on a nanodisc that controls its interaction with the Ras-binding domain (RBD) of Raf.^5,6^ A directional fly-casting model, with the membrane-distal conformation of K-Ras4B recruiting Raf to the membrane, has been proposed based on biophysical and computational studies.^7^ In contrast, an alternate nucleotide-specific configuration of the H-Ras G-domain associated with the membrane has been proposed based on a Förster resonance energy transfer (FRET) study^8^ and molecular dynamics (MD) simulations.^9,10^ Moreover, several studies have suggested Ras dimerization on membrane surfaces; however, the reported dimer interfaces were different.^11−15^

Most of the above-described studies used artificial membrane systems, such as dipalmitoyl lipid bilayers or nanodiscs in vitro^5−7,14,15^ or a fusion system consisting of fluorescent proteins and nanodomain markers, to indirectly observe Ras membrane orientation in vivo.^8^ Consequently, the Ras structure in the native cellular environment remains unclear. Therefore, direct observation of Ras association with membranes in living cells, especially at the atomic scale, is of interest to understand the structural basis of Ras activation in vivo. However, gaining structural insight into such flexible protein-membrane systems in living cells is challenging because the available observation tools are limited. In-cell NMR spectroscopy is one of the potential methods to analyze such challenging targets; it can investigate the behavior of bio-macromolecules at atomic resolution in living cells.^16−19^ A previous in-cell NMR study revealed that GTP-bound levels of H-Ras were modulated in the intracellular environment; however, this H-Ras construct was entirely distributed in the cytosol because it lacked its C-terminal HVR.^20^ Although the in-cell NMR signal of membrane proteins is considered almost undetectable resulting from the restricted rotational motion of the target molecules, Ras associates with membranes through a relatively long tether. Thus, providing some freedom of rotational motion to a G-domain could enable signal detection.

Herein, we explored the structural features of membrane-associated H-Ras in the native cellular environment using in-cell NMR spectroscopy. To overcome the low sensitivity of the in-cell NMR spectra, we used the site-specific ^19^F-labeled H-Ras to simplify the NMR spectra and a Bayesian spectral deconvolution to ensure the objectivity of our interpretation. We demonstrated that the exogenous H-Ras was assimilated by endogenous membrane-trafficking and adopted nucleotide-dependent multiple conformations relative to PM in the cell.

## Results and Discussion

### Incorporation of OCF_3_Phe into H-Ras for ^19^F-Labeling

^19^F-NMR is an attractive technique because the fluorine nucleus is the second most sensitive NMR-active nucleus and a 100% naturally abundant isotope, which can readily be observed in a simple 1D spectrum without any background in most biological samples.^21^ Many in-cell NMR studies utilizing ^19^F probes have been reported in yeast cells,^22^*Escherichia coli* cells,^23−25^*Xenopus laevis* oocytes,^25,26^ and *Danio rerio* oocytes.^27^ In addition, the ^19^F resonance of the trifluoromethyl (CF_3_) group is intrinsically narrow due to the short effective rotational correlation times arising from its fast rotation.^28^ Therefore, we site-specifically incorporated a *p*-trifluoromethoxyphenylalanine (OCF_3_Phe) (Figure 1b) into H-Ras wild-type (WT) protein at three different sites, namely, Tyr32, Tyr96, and Tyr157, which are referred to as ^19^F-Y32, ^19^F-Y96, and ^19^F-Y157 H-Ras WT, respectively. In spite of OCF_3_Phe incorporation, “WT” was added to the names of these constructs to distinguish them from their Q61L or C181S/C184S mutants. In the H-Ras protein, Tyr32 is located on switch I, and Tyr96 is in the vicinity of switch II (Figures 1c and S1). In contrast, Tyr157 is positioned on helix α5, neither close to the nucleotide-binding site (NBS) nor the effector-binding site (Figure 1c). We obtained ^19^F-Y32, ^19^F-Y96, and ^19^F-Y157 H-Ras WT proteins with ∼100, ∼50, and ∼70% OCF_3_Phe suppression efficiencies, respectively (Figure S2). Since the prematurely terminated byproducts without OCF_3_Phe incorporation were eliminated during protein purification, each full-length H-Ras protein should be site-specifically labeled with ^19^F at the desired site. The proper folding of each ^19^F-labeled H-Ras protein was confirmed with uniformly ^15^N-labeled samples (Figure S3).

### In Vitro Characterization of the ^19^F-Labeled H-Ras

To assess the effects of OCF_3_Phe incorporation on Ras functions, we performed in vitro characterization of each ^19^F-labeled H-Ras protein. First, the GTP hydrolysis activities were examined using the GTPase-Glo assay.^29^ The ^19^F-Y96 and ^19^F-Y157 H-Ras WT showed intrinsic and GTPase-activating protein (GAP)-stimulated GTPase activities similar to those of the H-Ras WT (Figures 1d and S4). In contrast, ^19^F-Y32 H-Ras WT exhibited a considerable ∼5-fold reduction in the intrinsic GTP hydrolysis activity. However, the GAP-stimulated GTP hydrolysis activity was slightly enhanced when compared to that of the H-Ras WT (Figures 1d and S4). In the presence of the catalytic domain of Son of Sevenless 1 (SOS^cat^; a guanine nucleotide-exchange factor (GEF) for Ras), the intrinsic GTP hydrolysis activity was further reduced for ^19^F-Y32 H-Ras WT. In contrast, no significant changes were observed for ^19^F-Y96 and ^19^F-Y157 H-Ras WT (Figures 1d and S4). Therefore, we investigated the nucleotide-exchange (NE) rate of ^19^F-Y32 H-Ras WT by real-time NMR.^30^ Compared with that of H-Ras WT, the intrinsic NE rate of ^19^F-Y32 H-Ras WT slightly increased by ∼1.6-fold, while the GEF-mediated NE rate reduced by ∼2-fold (Figure 1e and S5). These results indicate that ^19^F-Y96 and ^19^F-Y157 H-Ras WT do not disrupt the GTPase cycle. In contrast, ^19^F-Y32 H-Ras WT tends to adopt a GDP-bound form in the presence of both GAPs and GEFs, supported by the increase in GAP-stimulated GTP hydrolysis and reduced GEF-mediated NE activities.

Next, we examined effector binding using surface plasmon resonance (SPR) to measure the binding kinetics of each guanosine 5′-[β,γ-imido]triphosphate (GMPPNP; a nonhydrolyzable analog of GTP) bound ^19^F-labeled H-Ras WT protein to two different immobilized effectors’ RBDs, Raf1, and RGL. For the interaction with Raf1 RBD, the association rates (*k*_on_), dissociation rates (*k*_off_), and dissociation constants (*K*_D_) of all three ^19^F-labeled H-Ras WT proteins exhibited the same order of magnitude, as observed for the H-Ras WT (Figures 1f and S6). In contrast, for binding to RGL RBD, ^19^F-Y96 and ^19^F-Y157 H-Ras WT showed comparable *K*_D_ values to those of H-Ras WT (3–5 μM), while the *K*_D_ value of ^19^F-Y32 H-Ras WT substantially increased by one order of magnitude (38 μM) (Figures 1f and S7). These results indicate that ^19^F-Y96 and ^19^F-Y157 H-Ras WT do not interfere with the effector binding, whereas ^19^F-Y32 H-Ras WT affect the binding depending on the effectors.

### In Vitro NMR Studies of the ^19^F-Labeled H-Ras

**Figure 2:**
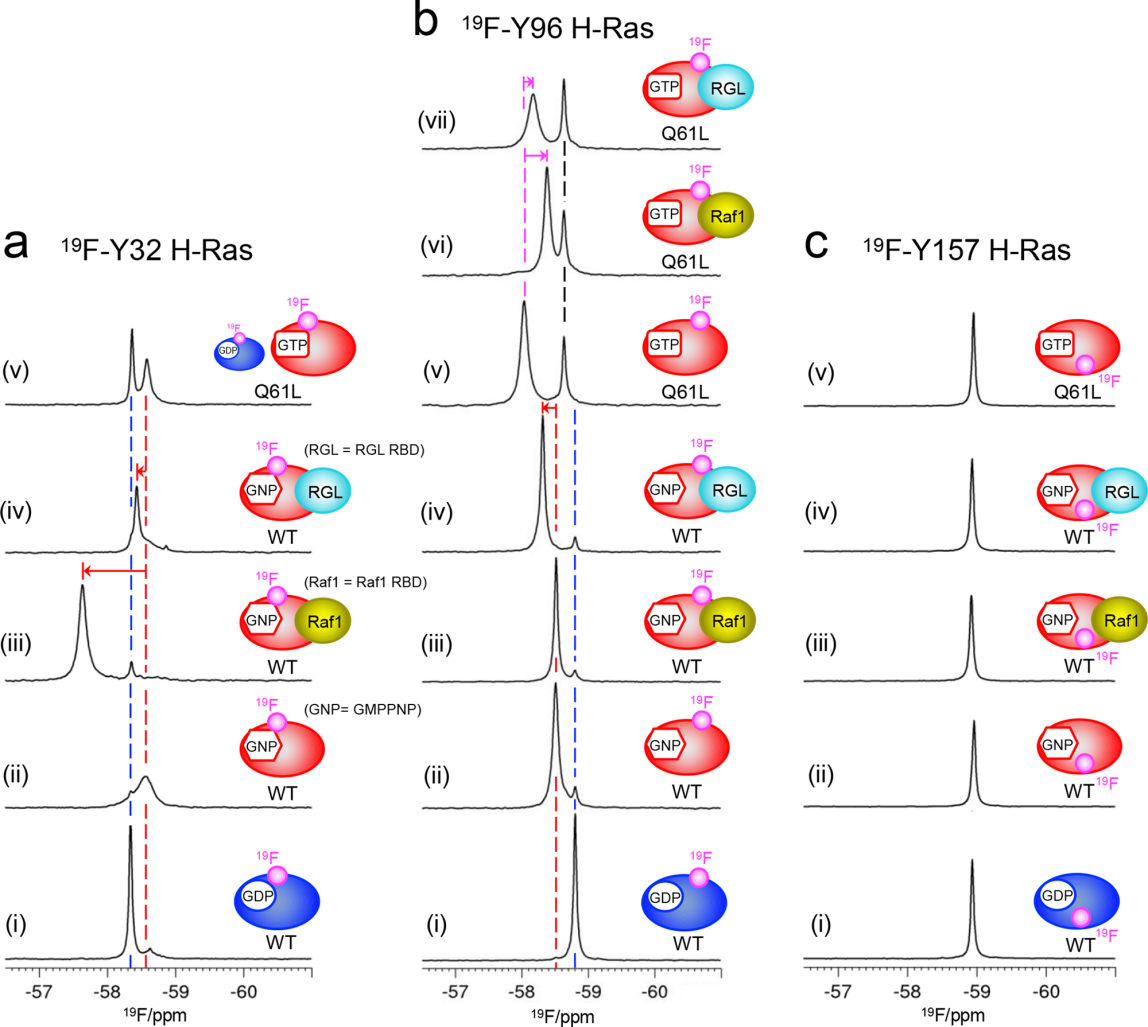
In vitro ^19^F-NMR analysis of the ^19^F-Y32 (a), ^19^F-Y96 (b), and ^19^F-Y157 (c) H-Ras proteins. GDP-bound WT (i), GMPPNP-bound WT (ii), GMPPNP-bound WT in the presence of Raf1 RBD (iii) and RGL RBD (iv), and Q61L mutant (v) are shown in each ^19^F-labeled H-Ras (a)–(c). In (b), the Q61L mutants in the presence of Raf1 RBD and RGL RBD are shown in (vi) and (vii), respectively. In (a) and (b), blue and red dashed lines show the chemical shifts corresponding to the GDP- and GMPPNP-bound forms, respectively, and red arrows indicate the chemical shift changes upon adding the effectors. In (b), magenta arrows show the chemical shift changes upon adding the effectors to the Q61L mutant. The expected ^19^F-labeled H-Ras are illustrated using cartoon models in each spectrum.

The ^19^F-Y32 H-Ras WT displayed a narrow signal (27.5 Hz) in the GDP-bound form and a broad signal (164.8 Hz) with an upfield shift by −0.22 ppm in the GMPPNP-bound form (Figure 2a(i) and (ii)). Since the NE procedure of the ^19^F-Y32 H-Ras WT from a GDP to a GMPPNP was imperfect (Figure S8), the residual GDP-bound signal was still observed in the GMPPNP-bound spectrum. Generally, an upfield shift (shielding) is observed when fluorine is in close contact with H-bond donors or water molecules, whereas a downfield shift (deshielding) is observed preferentially in close contact with hydrophobic side chains or with the carbon of carbonyl groups of the protein backbone.^31^ The upfield shift in the GMPPNP-bound form can be explained by the crystal structures of H-Ras, wherein the aromatic side chain of Tyr32 points toward the inside of the protein in the GDP-bound form^32^ but is exposed to the solvent in the GMPPNP-bound form^33^ (Figure S1). The broad signal observed for the GMPPNP-bound form indicates a conformational exchange in switch I, which is consistent with earlier results that switch I adopts at least two different conformational states (the low-affinity state I and the effector-binding state II).^34−36^ The ^19^F-Y96 H-Ras WT also showed a narrow signal (30.3 Hz) in the GDP-bound form, and a slightly broadened signal (55.6 Hz) shifted downfield by 0.29 ppm in the GMPPNP-bound form (Figure 2b(i) and (ii)). The downfield shift of the GMPPNP-bound form could reflect the interaction of the CF_3_ group with a carbonyl group of Gly60 in switch II, which is observed in the crystal structure of GMPPNP-bound H-Ras^33^ (Figure S1). The ^19^F-Y157 H-Ras WT exhibited a narrow signal in both the GDP-bound (27.6 Hz) and GMPPNP-bound (32.0 Hz) forms at almost the same chemical shifts (Figure 2c(i) and (ii)), as expected from its ^19^F-labeling site, where Tyr157 is far from the NBS (Figure 1c).

We next titrated the Raf1 RBD and RGL RBD to each ^19^F-labeled H-Ras WT protein. Both RBDs did not induce chemical signal changes for any of the GDP-bound ^19^F-labeled H-Ras proteins, although slight line-broadening was observed, especially in the presence of Raf1 RBD (Figure S9). In contrast, the GMPPNP-bound signal of ^19^F-Y32 H-Ras WT shifted downfield upon the addition of Raf1 RBD (0.93 ppm) and RGL RBD (0.13 ppm) (Figure 2a(iii) and (iv)). These chemical shift changes are consistent with the crystal structures of H-Ras in complex with the effectors. The aromatic side chain of Tyr32 considerably moves toward the P-loop in complex with the Raf1 RBD.^37^ In contrast, its movement is minimal when complexed with the RalGDS RBD^38^ (homologous to RGL RBD) (Figure S10a). For the ^19^F-Y96 H-Ras WT, the GMPPNP-bound signal shifted downfield by 0.20 ppm upon the addition of RGL RBD, whereas almost no change was observed by Raf1 RBD addition (Figure 2b(iii) and (iv)). This corroborates with the crystal structures of H-Ras in complex with effectors,^37,38^ where Tyr96 is perturbed only in complex with the RalGDS RBD (Figure S10b). The GMPPNP-bound signal of ^19^F-Y157 H-Ras WT was only slightly perturbed upon the addition of either RBD, with a chemical shift change of ∼0.02 ppm (Figure 2c(iii) and (iv)).

### Analyses of Oncogenic H-Ras Q61L Mutants

The oncogenic Ras Q61L mutant reportedly exhibits reduced in vitro GTP hydrolysis activity^39,40^ and rapid NE,^39^ thereby resulting in a continuously activated state. The ^19^F-Y96 and ^19^F-Y157 H-Ras Q61L mutants were dominated by more than 90% of GTP-bound forms. However, approximately 30% of GTP was hydrolyzed in the ^19^F-Y32 H-Ras Q61L mutant (Figure S11). This hydrolyzation activity was possibly due to the disruption of the H-bond formation between the hydroxyl group of Tyr32 and γ-phosphate of GTP^41^ by CF_3_ group incorporation (Figure S12). The ^19^F-Y32 H-Ras Q61L mutant displayed sharp and broad signals at the same chemical shifts as those observed in the GDP- and GMPPNP-bound signals of the ^19^F-Y32 H-Ras WT, respectively (Figure 2a(i), (ii), and (v)). The broad signal should be assigned as the GTP-bound form; however, its linewidth (64.6 Hz) was much narrower than that of the GMPPNP-bound signal of its WT counterpart (164.8 Hz) (Figure 2a(ii) and (v)). This linewidth difference is believed to either reflect the different activated state of the Q61L mutant from that of the WT protein or is just a consequence of the difference between GTP- and GMPPNP-bound forms. As GMPPNP reportedly increases the population of low-affinity state I when compared to GTP or GTPγS,^42^ we prepared the GMPPNP-bound ^19^F-Y32 H-Ras Q61L mutant using the NE procedure. The linewidth of its GMPPNP-bound signal was 80.5 Hz, which is comparable to that of the GTP-bound signal (Figure S13), thereby indicating that the oncogenic Q61L mutant acquires an activated state different from the WT protein. This is consistent with the previous evidence that the Q61L mutant results in the acquirement of state II-like structural features even in the GMPPNP-bound form.^43^

The ^19^F-Y96 H-Ras Q61L mutant also displayed two signals, which should be assigned as the GTP-bound forms (Figure 2b(v)). However, neither signal overlapped with that of the GMPPNP-bound ^19^F-Y96 H-Ras WT (Figure 2b(ii) and (v)). Interestingly, only one signal at −58.0 ppm shifted upfield with the addition of the effectors (Figure 2b(vi) and (vii)), suggesting that this signal may correspond to the effector-binding state II and the other one could reflect the low-affinity state I.^36^ The ^19^F-Y157 H-Ras Q61L mutant showed a sharp signal at almost the same chemical shift observed for the GMPPNP-bound signal of its WT counterpart (Figure 2c(v)), as expected from its nucleotide-insensitive ^19^F-NMR signal.

### Exogenous Delivery of H-Ras Protein into HeLa Cells

**Figure 3:**
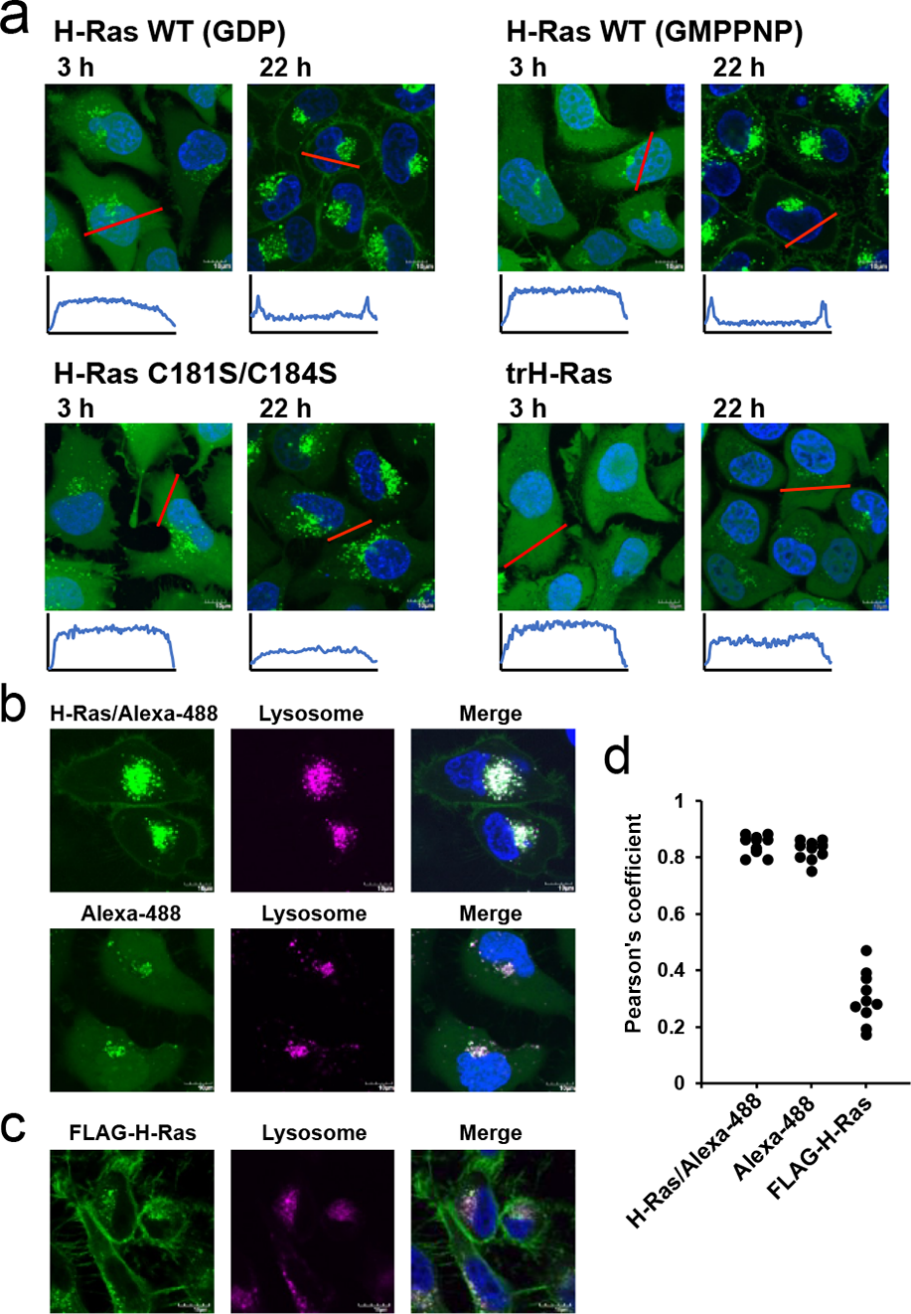
Intracellular distribution of the exogenous H-Ras in HeLa cells. (a) Representative images of the HeLa cells delivered with the Alexa 488 labeled H-Ras wild-type (WT) with GDP- and GMPPNP-bound forms, C181S/C184S mutant, and truncated H-Ras (trH-Ras) at 3 and 22 h after electroporation. The fluorescence intensity along the red line is shown below each cell image. (b) Colocalization of lysosomes with the Alexa 488-labeled H-Ras WT (H-Ras/Alexa-488) and with the monomeric Alexa 488 fluorophore. (c) Immunofluorescence microscopy of the FLAG-H-Ras and lysosome. (d) Pearson’s correlation coefficient of lysosome with the exogenous H-Ras and monomeric fluorophore.

Before performing in-cell NMR experiments, we confirmed the time dependence of intracellular distribution of the exogenously delivered H-Ras protein in HeLa cells by electroporation (EP)^44^ with the Alexa 488-labeled samples. The GDP-bound H-Ras was distributed in the cytosol 3 h after EP. However, it was substantially localized on the PM 22 h after EP and then continuously observed until 40 h after EP (Figures 3a and S14a). The GMPPNP-bound H-Ras also showed similar intracellular localization (Figure 3a). The PM-localized H-Ras amounts were linearly correlated with their delivery amount (Figure S14b). In addition to the PM localization, fluorescent foci were observed in the vicinity of nuclei irrespective of the delivery amount (Figures 3a and S14) and highly colocalized at the lysosome (Figure 3b,d). Hence, we further verified whether intact H-Ras proteins accumulate at the lysosome by immunofluorescence microscopy at 22 h after EP. The FLAG-tag fused H-Ras (FLAG-H-Ras) proteins were localized at the PM, similar to the case of the fluorescently labeled H-Ras delivery. However, the number of fluorescent foci in the vicinity of nuclei significantly reduced, and those foci were rarely colocalized with the lysosome (Figure 3c,d). An unconjugated monomeric fluorophore also accumulated at the lysosome (Figure 3b,d). These results suggest that most of the exogenous H-Ras in lysosomes were likely to be digested, although a possibility of a favorable lysosomal localization of the Alexa 488-labeled H-Ras rather than FLAG-H-Ras still remains. In contrast, a certain number of FLAG-H-Ras foci was still observed in the vicinity of nuclei (Figure 3c). This means that intact H-Ras proteins could be accumulated at endomembranes apart from lysosome transport, as lipidated H-Ras is known to undergo membrane-trafficking between the endomembrane system and PM.^2^ Therefore, we confirmed whether the exogenous H-Ras underwent lipid modifications by western blotting and mass spectrometry. To eliminate any effect from the endogenous H-Ras, the FLAG-H-Ras was delivered into HeLa cells and then purified from the cells 22 h after EP and confirmed by western blotting analysis (Figure S15). The purified FLAG-H-Ras underwent farnesylation and carboxyl methylation at position C186, as confirmed by liquid chromatography tandem mass spectrometry (LC–MS/MS) analysis (Figure S16). The lipidation-mediated membrane localization of the exogenous H-Ras was further supported by the data that the truncated H-Ras (trH-Ras; residues 1–171), lacking the C-terminal HVR, continuously distributed in the cytosol instead of PM localization even at 22 h after EP (Figure 3a). The H-Ras C181S/C184S mutant (a palmitoylation-deficient mutant) showed no PM localization at 22 h post-EP (Figure 3a), in line with previous findings that H-Ras cannot be released from Golgi without C181 and C184 palmitoylation.^45^ In addition, a small extent of cytosolic distribution was observed even at 22 h after EP (Figure 3a). Overall, our results show that the exogenous H-Ras was properly processed during the endogenous Ras trafficking in HeLa cells.

### Detection of the In-Cell ^19^F-NMR Signals of the ^19^F-Labeled H-Ras in HeLa Cells

**Figure 4:**
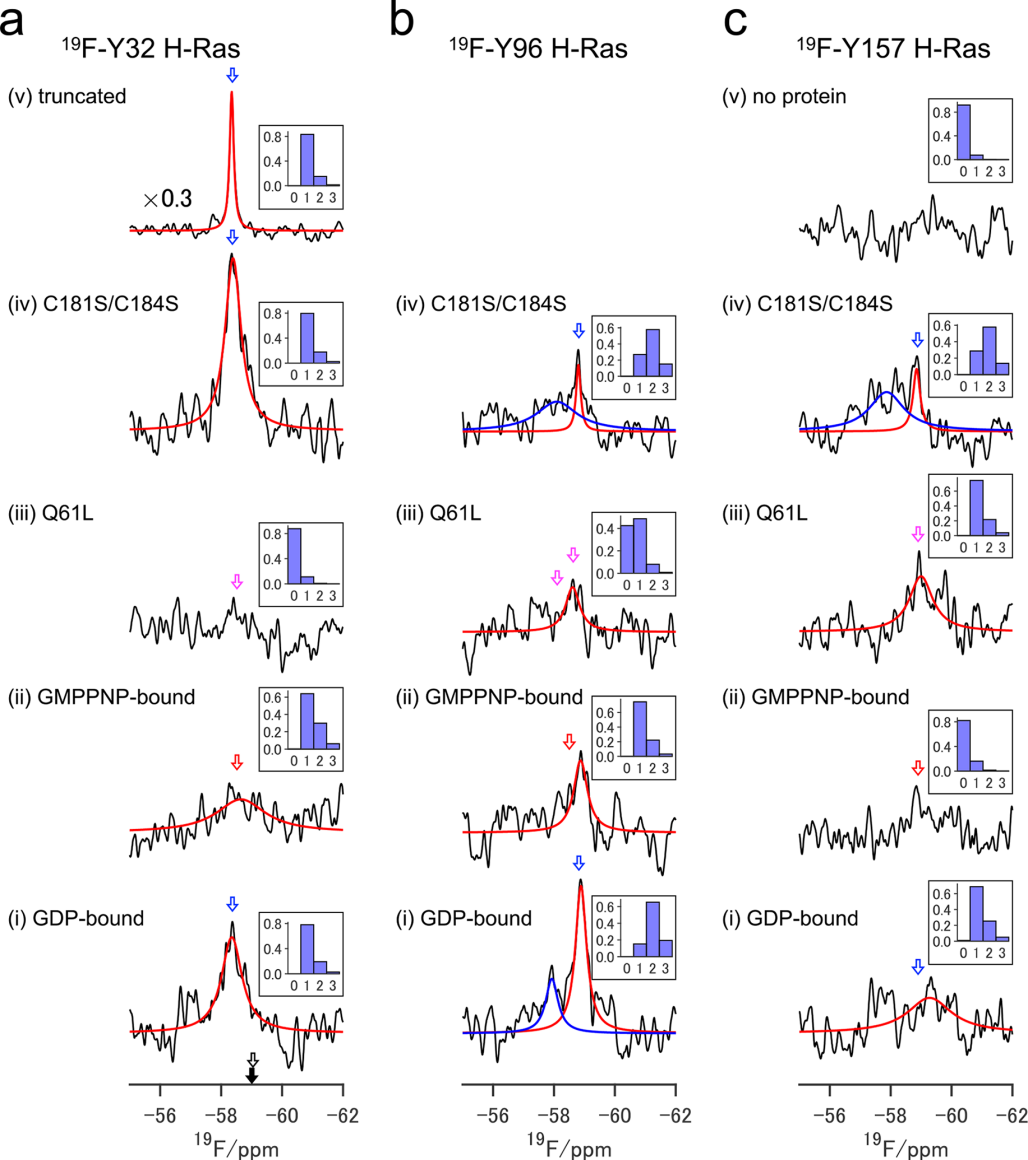
In-cell NMR spectra of the ^19^F-Y32 (a), ^19^F-Y96 (b), and ^19^F-Y157 (c) H-Ras proteins. The GDP-bound WT (i), GMPPNP-bound WT (ii), Q61L mutant (iii), and C181S/C184S mutant (iv) are shown in each ^19^F-labeled H-Ras (a)–(c). In (a), the truncated ^19^F-Y32 H-Ras is displayed with 0.3 times scale along the vertical axis in (v). In (c), the spectrum of no protein delivery is shown in (v). Red and blue lines represent deconvoluted signal components calculated by Bayesian inference using the Markov chain Monte Carlo (MCMC) method against the most probable number of signal components (either 1 or 2). The probabilities of the number of signal components obtained from the Bayesian free energies are inserted on each in-cell NMR spectrum. Blue, red, and magenta open arrows indicate corresponding in vitro chemical shifts of the GDP- and GMPPNP-bound signals of the WT and GTP-bound signals of the Q61L mutant. In (a), black open and filled arrows show the chemical shift of denatured ^19^F-Y32 H-Ras and free OCF_3_Phe, respectively.

The in-cell NMR experiments were initiated 22 h post-EP when the intracellular distribution of the exogenous H-Ras in HeLa cells was clearly observed (Figure 3a,c). More than 90% cell viability and no detectable NMR signals in the cell suspension media were observed after in-cell NMR measurements. We also recorded the ^1^H-NMR spectra of the HeLa cells before and after each in-cell NMR experiment. The peak intensities of lactate, phosphocholine, and mobile lipids, one of the biomarkers for cell metabolism,^46^ did not change significantly (Figure S17), indicating that each in-cell NMR experiment was conducted in the natural cellular environment. However, the intracellular GTP levels may be low in this condition, as expected from the result that intracellular ATP was entirely depleted without using a bioreactor, which continuously supplies fresh medium to the cells in an NMR sample tube.^47^ The ^19^F-NMR spectra of ^19^F-Y32, ^19^F-Y96, and ^19^F-Y157 H-Ras WT proteins showed worse sensitivity than those acquired in vitro (Figure 4). To verify the influence of nonspecific interaction with cellular components and lipid membranes on the in-cell NMR spectra of H-Ras, we performed in vitro NMR experiments in the presence of 10–40% (v/v) cell lysate and 0.15–0.27% (w/v) small unilamellar vesicle liposome. Neither of them induced any change in the ^19^F-NMR spectra of H-Ras (Figures S18 and S19). In contrast, the in-cell NMR spectrum of the ^19^F-Y32 trH-Ras, uniformly distributed in the cytosol (Figure 3a), exhibited signal broadening, mainly due to the reduced rotational diffusion of H-Ras molecules in the cytosol (Figures 4a(v) and S20). However, its linewidth was approximately 30 Hz, less than twice the corresponding in vitro signal, indicating that the worse NMR spectra sensitivity was not only due to the macromolecular crowding effect but rather influenced by the strongly restricted rotational motion of the H-Ras molecules tethered to the membrane compartments. Moreover, intense signals were observed for the 2% sodium dodecyl sulfate (SDS)-solubilized cellular fractions, thus confirming the accumulation of the exogenous H-Ras at the membrane compartments (Figure S21). These results indicate that the NMR signals of exogenously delivered H-Ras reflect the same native functional state as the endogenous one.

### Interpretation of the In-Cell NMR Spectra

Owing to the severely poor signal-to-noise (S/N) ratio of the in-cell ^19^F-NMR spectra, a Bayesian spectral deconvolution combined with the MCMC approach was adopted to ensure the objectivity of spectral interpretation. In contrast to the point estimation approach using local optimization methods such as least-squares fitting, spectral deconvolution by Bayesian inference can avoid falling into local minima and overfitting the noise. Therefore, it is more reliable for data interpretation. First, the probability of the number of signal components (0, 1, 2, and 3) was estimated by the Bayesian free energy, assuming the Lorentzian line shape (Figure S22). Then, the posterior probability distributions of three parameters, magnetization, chemical shift, and linewidth were calculated for each signal model^48^ (Figures S23 and S24). Since only a few signal components have been estimated in our study, this stepwise approach is simpler than a reversible-jump Markov chain Monte Carlo (MCMC) method, which simultaneously estimates the number of signal components and component parameters.^49^ Hereafter, magnetization, chemical shift, and linewidth are described as maximum-a-posteriori (MAP) estimators accompanied with 95% credible intervals (CIs) in parentheses otherwise noted, and their details are summarized in Figure S23 and Table S2. We confirmed that there were no signal components in the in-cell NMR spectra without any protein delivery based on Bayesian free energy analysis (Figure 4c(v)).

### Characterization of H-Ras Protein in the Living Cells

The GDP-bound ^19^F-Y32 H-Ras WT showed one signal component at the chemical shift corresponding to its in vitro spectrum with a linewidth of ∼400 (243–740) Hz (Figure 4a(i)). The GMPPNP-bound ^19^F-Y32 H-Ras WT also displayed one signal component; however, its linewidth was considerably broader at ∼1000 (502–4997) Hz (Figure 4a(ii)), as expected from an intrinsically broad signal of the corresponding in vitro spectrum (Figure 2a(ii)). The exogenous H-Ras were localized at the PM and accumulated at the endomembranes (Figure 3). However, these states were indistinguishable in their in-cell NMR spectra, possibly due to their line-broadening or overlapping. Consequently, based on Bayesian free energy analysis, one signal component was the most probable signal number. We also note that neither free OCF_3_Phe resulting from the lysosomal degradation nor denatured ^19^F-Y32 H-Ras WT signals were observed at those expected chemical shifts (−58.85 and −58.91 ppm, respectively; Figures 4a and S25), suggesting that the signal components in the in-cell NMR spectra were derived from the exogenously delivered H-Ras, which was correctly folded in the cells. By comparing peak integration of the in-cell ^19^F-NMR signal of the ^19^F-Y32 trH-Ras with that of its in vitro, an effective concentration of the exogenous H-Ras protein was estimated to be ∼10 μM, which is about one order of magnitude higher than that of endogenous H-Ras (∼1.6 μM).^50^ In contrast, the intracellular concentrations of Raf1 in HeLa cells are ∼0.013 μM,^50^ more than one order of magnitude lower than that of endogenous H-Ras. Therefore, the bound population of exogenous H-Ras with endogenous Raf1 was less than 1% based on its *K*_D_ value (Figure 1f). As most Ras effectors in human tissues are almost in the same concentration range as Raf1 and their affinities to Ras are generally weaker than Raf1,^51^ the in-cell NMR signal of the H-Ras with effector-bound forms would be undetectable. Despite sharper in vitro GTP-bound signals of the ^19^F-Y32 H-Ras Q61L mutant than those of the corresponding GMPPNP-bound WT (Figure 2a(ii) and (v)), its in-cell NMR spectra exhibited no signal components (Figure 4a(iii)). This suggests that the conformational equilibrium in the continuously activated oncogenic H-Ras is different from that in the activated H-Ras WT, thus inducing extensive line-broadening beyond NMR detection in the cells. The ^19^F-Y32 H-Ras C181S/C184S mutant showed one intense signal component (Figure 4a(iv)) that could be attributed to the H-Ras molecules in the cytosol, as seen in the intense in-cell NMR signal of the trH-Ras (Figure 4a(v)).

The GDP-bound ^19^F-Y96 H-Ras WT exhibited two signal components; the main component appeared with intense magnetization at the same chemical shift of its in vitro, and the second component shifted downfield (red and blue in Figure 4b(i)). Although the MAP linewidths of both signal components were almost the same at ∼200 Hz, the latter showed large posterior probability distribution with ∼6700 Hz of the upper limit of 95% CI (Figure S23 and Table S2). Two signal components were also observed for the ^19^F-Y96 H-Ras C181S/C184S mutant (Figure 4b(iv)). However, the linewidth of the main signal component (red in Figure 4b(iv)) was ∼40 (20–600) Hz, and its MAP estimator was comparable to that of trH-Ras (∼30 Hz), thereby indicating that the main signal component was derived from H-Ras distributed in the cytosol. Since the palmitoyl-deficient mutant cannot be localized at the PM, the second signal component likely reflected the endomembrane-accumulated state. Notably, the endomembrane-accumulated state could also be observed in the GDP-bound ^19^F-Y96 H-Ras WT, as the chemical shift of its second signal component (blue in Figure 4b(i)) was close to the second component of the C181S/C184S mutant (blue in Figure 4b(iv)). Consequently, the main signal component of the GDP-bound ^19^F-Y96 H-Ras WT (red in Figure 4b(i)) should be assigned as the PM-localized state since the H-Ras WT proteins have no distribution in the cytosol, unlike the palmitoyl-deficient mutant (Figure 3a). On the contrary, the GMPPNP-bound ^19^F-Y96 H-Ras WT showed one signal component (Figure 4b(ii)). Although the MAP estimator of the linewidth was ∼200 Hz, similar to that of the main component of the GDP-bound state, its posterior probability distribution was large with ∼1110 Hz of the upper limit of 95% CI (Figure S23 and Table S2), suggesting a potential wider linewidth. Similarly, the ^19^F-Y96 H-Ras Q61L mutant resulted in one signal component with a similar linewidth and a slightly smaller magnetization than that observed for the GMPPNP-bound ^19^F-Y96 H-Ras WT. However, the posterior probability of the no-signals model was comparable to that of the one-signal model, implying further signal broadening close to the noise level (Figure S22). Overall, all in-cell NMR spectra of the ^19^F-Y32 H-Ras and ^19^F-Y96 H-Ras revealed that H-Ras adopts different conformational states between its active and inactive states and other activated states between the WT and oncogenic Q61L mutant on membranes in the cells.

### Conformational Multiplicity of the H-Ras on the Membranes

In the case of the ^19^F-Y157 H-Ras WT, one signal component with weak magnetization and a significantly broader linewidth at ∼900 (325–5438) Hz was observed for the GDP-bound form, and no detectable signal component was obtained for the GMPPNP-bound form (Figure 4c(i) and (ii)). The ^19^F-Y157 H-Ras Q61L mutant showed one signal component whose linewidth was ∼460 (163–1535) Hz (Figure 4c(iii)). The ^19^F-Y157 H-Ras C181S/C184S mutant displayed two signal components; one was narrower, and the other was broader with ∼96 (43–1750) and ∼710 (256–1360) Hz linewidths, respectively (Figure 4c(iv)). Similar to the palmitoyl-deficient ^19^F-Y96 H-Ras mutant, the former and latter components obtained for this mutant could be assigned to the cytosolic distributed and the endomembrane accumulated H-Ras molecules, respectively. Considering the intrinsic narrow in vitro NMR signals of the ^19^F-Y157 H-Ras WT regardless of nucleotide-bound states (Figure 2c), the extensive signal broadening in its in-cell NMR spectra cannot be explained only by the restricted rotational motion of the H-Ras molecules tethered to the PM. Instead, it could reflect on the exchange-induced signal broadening, probably due to the transient interaction of the helix α5 with the PM. Previous FRET analysis^8^ and MD simulation^10^ demonstrated the interaction of helix α5 with the membrane depending on nucleotide-bound states, where the helix α5 lies on the membrane surface (membrane-associated) in the GTP-bound form, while it is rather distant from the protein-membrane interface (membrane-distinct) in the GDP-bound form. A similar nucleotide-dependent orientation preference of the G-domain in respect to the PM has been proposed for a Ras homolog enriched in the brain (Rheb) by NMR^52^ and MD^53^ studies. However, these preferable configurations are considered to be a population shift between the GTP- and GDP-bound states because membrane orientation of the G-domain is highly dynamic through C-terminal HVR.^7^ Therefore, the helix α5 potentially interacts with the PM in both nucleotide-bound states that are consistent with the extensive signal broadening of the in-cell NMR spectra of the ^19^F-Y157 H-Ras WT without depending on its nucleotide-bound states (Figure 4). However, since there is no detectable in-cell NMR signal of the GMPPNP-bound form (Figure 4b), the membrane-associated population would be rather pronounced for the GMPPNP-bound form, which is consistent with the previous observations in FRET- and MD-based studies.^8,10^ In contrast to GMPPNP-bound ^19^F-Y157 H-Ras WT, the ^19^F-Y157 H-Ras Q61L mutant showed one signal component whose line width and its upper CI limit were smaller than those of GDP-bound ^19^F-Y157 H-Ras WT (Figure 4c and Table S2). These results suggest that the oncogenic mutation may alter the population balance with respect to WT. Our findings indicate that H-Ras adopts conformational multiplicity on the PM in the native cellular environment. A conformational multiplicity of the Ras G-domain on the PM has also been proposed in a previous study on K-Ras4B; the membrane-bound and -tethered conformations coexist in fast dynamic exchange that can effectively recruit Raf for activation at the PM.^7^

## Conclusions

This study demonstrates that utilizing in-cell NMR spectroscopy combined with site-specific incorporation of OCF_3_Phe enables the structural interpretation of membrane-associated H-Ras in the native cellular environment. We also showed that Bayesian spectral deconvolution helps extract reliable data from the low S/N NMR data. The strategies demonstrated here provide a unique picture of the membrane-associated states of H-Ras at atomic resolution in living cells, which will further provide structural insights into another membrane-protein system in an intact manner.

## Methods

### Expression and Purification of H-Ras

All expression constructs of human H-Ras WT, and Q61L and C181S/C184S mutants were prepared using the plasmid pk7b2-NHisRas,^54^ which contains an NHis tag and a tobacco etch virus (TEV) protease recognition site, by overlap PCR using an In-Fusion Cloning Kit (Clontech). Additionally, H-Ras was fused with a FLAG-tag by inserting the FLAG sequence upstream of the H-Ras sequence with a short linker. All H-Ras proteins were expressed by the cell-free protein synthesis system using an *E. coli* cell extract with the dialysis method, where four reactions were set up in parallel, each containing a 9 mL inner reaction mixture in a dialysis tube immersed in a plastic container with 90 mL of outer solution, as previously described.^55−57^ The reaction was performed at 30 °C overnight with gentle shaking. The cell-free expression mixture was collected from the dialysis tube and diluted 3-fold with 20 mM Tris-Cl (pH 8.0) containing 300 mM NaCl, 5 mM imidazole, and 1 mM tris(2-carboxyethyl)phosphine (TCEP). The diluted mixture was centrifuged and filtered, and the clear solution was then applied to a HisTrap column (Cytiva). The bound proteins were eluted with a high concentration of 500 mM imidazole, which was subsequently removed using a HiPrep desalting column (Cytiva). The NHis tag was cleaved by incubating H-Ras proteins with TEV protease at 4 °C overnight and separated using a HisTrap column (Cytiva). The H-Ras proteins were further purified by chromatography using a HiTrap Q anion exchange column (Cytiva) and a Superdex 75 size exclusion column (Cytiva). For the expression of OCF_3_Phe incorporated ^19^F-labeled H-Ras proteins (^19^F-Y32, ^19^F-Y96, ^19^F-Y157 H-Ras WT, and Q61L and C181S/C184S mutants), the cell extract prepared from *E. coli* harboring a streptavidin-binding peptide (SBP)-tag fused release factor 1 (RF-1) on chromosome was treated with streptavidin beads to selectively remove RF-1 as previously described.^58^ In addition, 0.4 mg/mL OCF_3_Phe-tRNA synthetase (OCF3Phe-RS), 0.2 mg/mL sup-tRNA, and 1.5 mM OCF_3_Phe were additionally added to the inner reaction mixture, and 5 mM OCF_3_Phe was supplemented in the outer solution. The typical yield of the OCF_3_Phe-incorporated H-Ras was ∼10–30 mg/reaction tube, sufficient for a single in-cell NMR experiment.

### In Vitro NMR Spectroscopy

All in vitro 1D ^19^F-NMR experiments were performed using a 0.2 mM protein in 25 mM HEPES-KOH (pH 7.2) containing 120 mM KCl, 5 mM KH_2_PO_4_,10 mM MgCl_2_, and 1 mM DTT with 10% D_2_O at 565 MHz and 37 °C on a Bruker Avance III 600 MHz NMR spectrometer equipped with a QCI-F CryoProbe and processed with TopSpin (version 3.6, Bruker BioSpin); an exponential window function with a line-broadening of 20 Hz was used. The recycle time (acquisition plus delay) was 1.5 s. Spectra were acquired as 1024 scans of 65,536 complex points over a 237 ppm sweep width, for a total experimental time of ∼30 min. For the titration experiments of Raf1 RBD or RGL RBD, a solution containing 0.1 mM ^19^F-labeled H-Ras was mixed with an equimolar amount of Raf1 RBD or RGL RBD; spectra were acquired as 2048 scans. The ^19^F chemical shift values were referenced using 0.05% trifluoroacetic acid (TFA; −76.55 ppm). The linewidths of the ^19^F-NMR signals were defined with full width at half maximum (FWHM). ^1^H-^15^N correlation spectra of the ^15^N-labeled H-Ras proteins were acquired using a SOFAST-HMQC pulse sequence^59^ with 150 ms recycle time on a Bruker Avance III 600 MHz NMR spectrometer with a QCI-F CryoProbe at 37 °C. 1D ^1^H-NMR spectra of HeLa cells were recorded with the excitation sculpting sequence for water suppression. Spectra were acquired as 190 scans of 16,384 points over a 14 ppm sweep width, for an experimental time of ∼10 min.

### Cell Culture

HeLa cells were kindly provided by Dr. Takehisa Matsumoto at RIKEN Center for Biosystems Dynamics Research (BDR). The cells were grown at 37 °C under a 5% CO_2_ humidified atmosphere using high-glucose Dulbecco’s modified Eagle medium (DMEM; Thermo Fisher Scientific) supplemented with 10% fetal bovine serum (FBS; Thermo Fisher Scientific), 200 U/mL penicillin (PCN; Nacalai Tesque), and 200 μg/mL streptomycin (STR; Nacalai Tesque). For simplicity, we referred to this culture as DMEM unless stated otherwise.

### Intracellular Localization of the Fluorescent-Labeled H-Ras

For the fluorescent labeling of H-Ras WT and C181S/C184S mutants, solutions containing 0.5–0.7 mM H-Ras proteins in 100 mM sodium phosphate buffer (pH 8.3) were gently mixed with a 3-fold molar excess of Alexa Fluor 488 5-SDP Ester (Thermo Fisher Scientific) for 2 h at room temperature (∼25 °C). Excess free dyes were removed by buffer exchange with electroporation buffer (EPB; 25 mM HEPES-KOH (pH 7.2) containing 120 mM KCl and 5 mM KH_2_PO_4_) using Zeba Spin Desalting Columns (Thermo Fisher Scientific). The fluorescently labeled H-Ras proteins were delivered into HeLa cells by EP, as described previously,^44^ except using only a single high power poring pulse (100 V, 15 ms) in place of a combination of high-power poring and low power transfer pulses. Briefly, a total of 1.25 × 10^6^ HeLa cells were suspended in a 0.1 mL solution of 0.5–0.7 mM protein in EPB. Immediately after EP, cells were spread onto type-I collagen (Nippi)-coated 35 mm glass-based dish (IWAKI) containing DMEM and were incubated at 37 °C under a 5% CO_2_ humidified atmosphere for the desired durations as described in the text. DMEM was removed from the dish, followed by rinsing the cell surface twice with Dulbecco’s phosphate buffered saline (D-PBS; Nacalai Tesque) and then refilling the dish with DMEM without phenol red (Nacalai Tesque), supplemented with 10% FBS, 200 U/mL penicillin, and 200 μg/mL streptomycin. For nuclear staining, 2–3 drops of NucBlue Live ReadyProbes Reagent (Hoechst 33342; Thermo Fisher Scientific) were added to the media. All fluorescent images of the HeLa cells were acquired using confocal laser scanning microscopy (FLUOVIEW FV10i-DOC; OLYMPUS).

### In-Cell NMR Spectroscopy

The ^19^F-Y32, ^19^F-Y96, and ^19^F-Y157 H-Ras WT proteins and their respective Q61L and C181S/C184S mutants were delivered into HeLa cells by EP as described above, except that a total of 1.25 × 10^7^ cells were suspended in a 1 mL of 1 mM protein solution. The cells were incubated at 37 °C under a 5% CO_2_ humidified atmosphere for 22 h and then transferred into an NMR tube. In the case of trH-Ras, the incubation time after EP was 3 h. All in-cell 1D ^19^F-NMR spectra were recorded using the acquisition parameters similar to in vitro 1D ^19^F-NMR spectroscopy, except 7168 scans were acquired. The total experimental time was less than 3 h, which was within the lifetime of cells in an NMR sample tube. The viability of the HeLa cells after in-cell NMR measurements was confirmed using a Tali Viability Kit—Dead Cell Red (Thermo Fisher Scientific). After each in-cell NMR measurement, the HeLa cells were collected and separated from the suspending media by gentle centrifugation (200*g* × 5 min, repeated twice). The collected HeLa cells were sonicated in EPB with 10% D_2_O for lysis. Subsequently, the cell lysate was separated from the cell pellet by centrifugation (20,000*g* × 30 min). The membrane fractions of the HeLa cells were further solubilized from the cell pellets using 2% SDS solution with 10% D_2_O. The 1D ^19^F-NMR spectra of the suspending media, the cell lysate, and the membrane fractions were recorded using the same parameters with in-cell NMR measurement.

### Data Analysis for In-Cell NMR Spectra

Bayesian spectral deconvolution, assuming the absorptive Lorentzian line shape and the uniform prior distribution, was performed. The number of signals was estimated based on Bayes free energy calculated with the thermodynamic integration using MCMC runs at various temperatures.^48,60,61^ The posterior distribution of signal parameters, namely, magnetization, chemical shift, and linewidth, was also evaluated by MCMC. The preprocessing of data and the Bayesian inference were performed with MATLAB R2020a (MathWorks) software. First, the raw free induction decay (FID) data of in-cell ^19^F-NMR were processed by a MATLAB script which functioned as the same as “rm_digital_filter” of the nmrglue v.0.7 program.^62^ Then, the first 4096 complex points, which corresponded to the acquisition time period of 30.6 milliseconds, were zero-filled to 8192 points and Fourier-transformed without any apodization functions applied. Note that we confirmed that all of the in-cell NMR signals were sufficiently attenuated within 30.6 milliseconds. Each spectrum was baseline-corrected by the fourth-order polynomial curve fitting. The real part of the spectrum is used for the subsequent Bayesian analysis.

We assumed that each signal is absorptive Lorentzian:
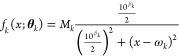
where *f_k_* is the model function for the *k*-th signal, **θ**_*k*_ = {*M_k_*, ω_*k*_, β_*k*_} is the explanatory variable, *M_k_* is the macroscopic magnetization, or the peak area, ω_*k*_ is the chemical shift, and β_*k*_ is the common logarithm of the FWHM. For the Bayesian inference, we adopted the uniform prior distributions within specified ranges, from 0 to 700 arbitrary unit (a.u.) for *M_k_*, from −62 to −55 ppm for ω_*k*_, and from 1 to 4 log_10_(Hz), which corresponded to 10 to 10,000 Hz of FWHM, for β_*k*_. Let *K* denote the total number of signals. The model function of the observed spectrum is the sum of the individual signals:
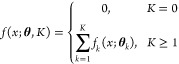


Let ***D*** = {*x_n_*, *y_n_*}_*n* = 1_^*N*^ be the spectral data, where *x_n_* is the chemical shift, *y_n_* is the intensity, and *N* = 8192 is the number of data points in the spectrum. Because of the low signal-to-noise ratio of in-cell NMR, the thermal noise is the dominated noise source. Together with applying no apodization function, we can safely assume the spectral noise is white Gaussian. Therefore, the log likelihood function is
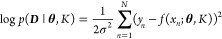
where σ^2^ is the variance of the noise, which is estimated using the latter part, namely, after 30.6 ms, of the FID.

In general, by MCMC at an inverse temperature β ∈ [0,1], we can obtain MCMC samples **θ**^β,1^...**θ**^β, *T*^ following the posterior probability^60,61^
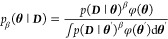


where φ is the prior probability of **θ**. The expected value of the function *G*(**θ**) over *p*_β_(**θ** | ***D***) can be approximated using the MCMC samples:^63^
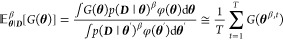


Suppose *K* is unknown and should be estimated using the data ***D***, the Bayes free energy is defined by^60,61^
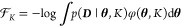


In this study, we calculated  by the thermodynamic integration using temperature steps 0 = β_0_ < β_1_ < ... < β_*J*_ = 1^48,60,61^
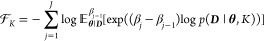


The mcmcstat software^63^ was used to perform MCMC at 46 temperature steps, namely, *J* = 45, β_1_ = 0.01, and β_*j* – 1_/β_*j*_ = 0.9006 for *j* ≥ 2. The total MCMC steps, the burn-in steps, and the thinning interval were set to be 400,000, 200,000, and 100, respectively, so that the number of MCMC samples was 2000. For each {β, *K*}, 19 individual MCMC chains were generated from random initial values. To avoid local minimum problem, we excluded at maximum 9 outliers out of 19 chains found by generalized extreme Studentized deviate (ESD) test^64^ evaluating  using ‘isoutlier’ command of MATLAB. Therefore, the total number of MCMC samples *T* for each {β, *K*} varied from 20,000 (2000 × 10) to 38,000 (2000 × 19) depending on how many chains were excluded as the outliers. The free energy was calculated for all *K* in the candidate set . We set the maximum *K* as 3, namely, , for two reasons. The first was by visual inspection we could find two signals at maximum. The second, which was more practical reason, was that we could not meaningfully discuss many signals due to the low signal-to-noise ratio. In addition, we confirmed that  in all cases, implying monotonic increase of  for *K* ≥ 4. The posterior probability of *K* was calculated by
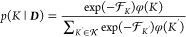


In this study, the prior probability φ(*K*) was assumed to be constant.

Once *K* is given, the posterior probability of **θ** is
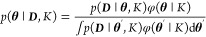


In this study, we performed MCMC at β = 1 to evaluate *p*(**θ** | ***D***, *K*) using the mcmcstat software^63^ The total MCMC steps, the burn-in steps, and the thinning interval were set to be 100,050,000, 50,000, and 200, respectively, so that the number of MCMC samples *T* = 500,000. Let **π**(**θ**^*t*^) denotes a set of all *K*! possible permutations of {**θ**_1_^*t*^, ..., **θ***_K_^t^*}. For example, when *K* = 2, **π**(**θ**^*t*^) = {{**θ**_1_^*t*^, **θ**_2_^*t*^}, {**θ**_2_^*t*^, **θ**_1_^*t*^}}. For each MCMC sample **θ**^*t*^, *t* = 1...*T*, arbitrary permutation of the signal order gives the same posterior probability, i.e.,
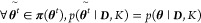


This means that the posterior distribution is *K*!-fold symmetry due to the arbitrary order of *K* chemical species. Therefore, if *K* ≥ 2, the marginal posterior distributions of the parameters of interest, *M_k_*, ω_*k*_, and β_*k*_, may be unfavorably contributed from the different chemical species. To avoid this problem, after the MCMC sampling of **θ**^1^, ..., **θ**^*T*^, we reordered the signals by the following iterative procedure. At first, an initial anchor point **θ̅** is randomly selected from the MCMC samples. Subsequently, **θ**^1^, ..., **θ**^*T*^ and **θ̅** are updated alternately in an iterative manner:
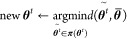

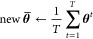


The iterations are executed until convergence or until the number of iterations is reached to 20. We adopted the standardized Euclidean distance as the distance measure in the parameter space:
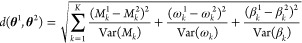


The histogram of the reordered MCMC samples was presented as the joint or the marginal posterior distribution. The reordered MCMC sample with the largest posterior density was presented as the MAP estimator. The limits of the credible interval were defined by the corresponding percentile points of the reordered MCMC samples.

While no apodization function was applied for the numerical analysis as mentioned, only for the visualization purpose, the exponential apodization function with the decay constant, or the line-broadening factor, of 200 Hz was applied prior to the Fourier transform to both the model FID, which was reconstructed from the MAP estimator, and the observed FID.

## Supporting Information Available

Experimental details; cell-free expression results of the ^19^F-labeled H-Ras; in vitro characterizations of the ^19^F-labeled H-Ras; time-course cell images of the HeLa cells delivered with H-Ras; western blot and LC–MS/MS analyses of the FLAG-H-Ras; ^1^H-NMR spectra of the HeLa cells; NMR spectra of the ^19^F-labeled H-Ras in the presence of cell lysates and liposomes; in-cell NMR spectrum of the ^19^F-Y32 trH-Ras; ^19^F-NMR spectra of the cell lysate and cellular fraction solubilized with 2% SDS; Bayesian spectral deconvolution details; and ^19^F-NMR spectra of a free OCF_3_Phe and denatured ^19^F-labeled H-Ras (PDF)
