# Supplementary material for: Direct Observation of Membrane-Associated H-Ras in the Native Cellular Environment by In-Cell 19F-NMR Spectroscopy
Source: JACS Au. 2023 Jun 1;3(6):1658–69. doi: 10.1021/jacsau.3c00108 (PMC10302746; doi:10.1021/jacsau.3c00108)
Supplement: Supplementary file 1 — au3c00108_si_001.pdf [file au3c00108_si_001.pdf]

Supporting information

Direct Observation of Membrane-Associated H-Ras in the Native Cellular Environment by In-Cell ^{19}F-NMR Spectroscopy

Masaomi Ikari[a‡], Hiromasa Yagi[a‡], Takuma Kasai [a,b‡], Kohsuke Inomata[a,b], Masahiro Ito[a], Kae Higuchi[a], Natsuko Matsuda[a,c], Yutaka Ito[d], and Takanori Kigawa[a*]

[a]RIKEN Center for Biosystems Dynamics Research, Kanagawa 230-0045, Japan

[b]PRESTO/Japan Science and Technology Agency, Saitama 332-0012, Japan

[c]SI Innovation Center, Taiyo Nippon Sanso Corporation, Tokyo 206-0001, Japan

[d]Department of Chemistry, Graduate School of Science, Tokyo Metropolitan University, Tokyo 192-0397, Japan

[‡]These authors contributed equally to this work.

[*]Corresponding author. Email: kigawa@riken.jp

Table of Contents

1. Experimental Section..2
2. Supporting Figures 9
3. Supporting Tables..40

1. Experimental Section

Expression and Purification of OCF$_3$Phe-RS

For expression of the *p*-trifluoromethoxyphenylalanine (OCF$_3$Phe) tRNA synthetase (OCF$_3$Phe-RS), the gene encoding the tyrosyl-RS mutant (Y32A/L65S/F108Q/Q109A/D158A/L162Y) from *Methanocaldococcus jannaschii*, which can specifically recognize OCF$_3$Phe[1], was synthesized and cloned into the plasmid pK7[2] with an additional tobacco etch virus (TEV) protease recognition site and a 6-histidine tag at the C-terminus, by overlap PCR using an In-Fusion Cloning Kit (Clontech). OCF$_3$Phe-RS was expressed by the cell-free protein synthesis system using an *Escherichia coli* cell extract with dialysis method, where four reactions were set up in parallel, each containing a 9 mL inner reaction mixture in a dialysis tube immersed in a plastic container with 90 mL of outer solution as previously described[3-5]. The reaction was performed at 30 °C overnight with gentle shaking. The cell-free expression mixture was collected from the dialysis tube and diluted 3-fold with 20 mM Tris-Cl (pH 8.0) containing 300 mM NaCl, 5 mM imidazole, and 1 mM tris(2-carboxyethyl)phosphine (TCEP). The diluted mixture was cleared by centrifugation and filtration, and then applied to a HisTrap column (Cytiva). The bound proteins were eluted with 500 mM imidazole, and the high concentration of imidazole was subsequently removed by a HiPrep desalting column (Cytiva). The NHis tag was cleaved by TEV protease at 4 °C overnight and separated from OCF$_3$Phe-RS by a HisTrap column (Cytiva). The OCF$_3$Phe-RS protein was further purified by chromatography on a HiTrap Q anion exchange column (Cytiva) and a Superdex 75 size exclusion column (Cytiva).

Preparation of Sup-tRNA

The transcription vector for the amber suppressor tRNA (sup-tRNA), which is the mutant of the tyrosyl-tRNA from *M. jannaschii* (5′-CCGGCGGUAGUUCAGCAGGGCAGAACGGCGGACU<u>CUA</u>AAUCCGCAUGGCGCUGGUUCAAAUCCGGCCCGCCGGACCA-3′, anticodon is underlined)[6], was cloned into the pUC19 plasmid with a T7 promoter attached at the 5′-end (pUC19-*Mj*Tyr-tRNA-CUA). The gene encoding sup-tRNA was amplified by PCR with the T7 promoter at the 5′-end from the plasmid pUC19-*Mj*Tyr-tRNA-CUA and purified using a PCR clean-up system (Promega). The sup-tRNA was transcribed from the amplified gene using a MEGAshortscript^{TM} T7 Transcription Kit (Invitrogen) at 37 °C for over 40 h, and then purified by phenol-chloroform extraction and ethanol precipitation.

Nucleotide Exchange

To obtain GMPPNP-bound H-Ras protein, the bound nucleotide was exchanged by an incubation with a 10-fold molar excess of GMPPNP (Merck) and alkaline phosphatase (AP; 4 U/mg of H-Ras protein), in 40 mM Tris-Cl (pH 7.5) containing 200 mM NaCl, 10 mM $MgCl_2$, 10 μM $ZnCl_2$, and 2 mM TCEP at 37 °C for 2 h[7]. Free nucleotides and AP were removed by passage through a Superdex 75 size exclusion column (Cytiva). The quantity of the Ras-bound nucleotide was analyzed by chromatography on a Mono Q column (Cytiva), as follows. A small portion of the H-Ras protein solution was mixed with an equal volume of 10% trichloroacetic acid to denature and precipitate the H-Ras protein. The bound nucleotides

should be extracted in the supernatant. The precipitations were removed by centrifugation (20,000 *g* × 5 min), and then the supernatant was lyophilized. The extracted nucleotides were redissolved in 20 mM Tris-Cl (pH 8.0) containing 50 mM NaCl, and then loaded onto a Mono Q column (Cytiva). The nucleotides were eluted by a linear gradient of 50 - 500 mM NaCl.

Expression and Purification of Raf1 RBD and RGL RBD

The Ras-binding domains (RBDs) from human Raf1 (Raf1 RBD; residues 51–131) and human RGL (RGL RBD; residues 631–735) were cloned into the pGEX-6P-3 plasmid (Cytiva) with an N-terminal GST tag and a PreScission protease recognition site. The GST-tagged Raf1 RBD and RGL RBD proteins were expressed in *E. coli* BL21 (DE3) pLysS strain. Cells were grown overnight in 5 mL of LB media and subsequently inoculated into 1 L of LB media at 37 °C. The protein expression was induced by adding 1 mM isopropyl β-D-1-thiogalactopyranoside (IPTG) when the cells reached an OD_{600} of 0.6, and the culture was continued at 24 °C overnight. For the purification of the Raf1 RBD and RGL RBD, the cells were lysed by sonication in 20 mM Tris-Cl (pH 8.0) containing 200 mM NaCl, protease inhibitors (Roche), and 2 mM dithiothreitol (DTT). The lysate was centrifuged, and then the supernatant was applied to a GSTrap column (Cytiva). The bound proteins were eluted with reduced glutathione, and then the GST tag was cleaved by PreScission protease (Cytiva) at 4 °C overnight. The Raf1 RBD and RGL RBD proteins were further purified by chromatography on a Superdex 75 size exclusion column (Cytiva).

GTP Hydrolysis Assay

The intrinsic GTP hydrolysis assay was measured by the GTPase-Glo assay system[8] (Promega) according to the manufacturer's instructions. Briefly, H-Ras proteins were serially diluted in the GTPase/GAP buffer to a range of concentrations (0, 2.5, 5, 10, 20, and 40 μM), and a 5 μL of each solution was mixed with 5 μL of 10 μM GTP in a low-flange white 384-well plate (Corning). The reaction was incubated at room temperature (~25 °C) protected from light for 90 min at 23 °C. Subsequently, a 10 μL of GTPase-Glo Reagent mix was added to each well and incubated while shaking for 30 min at 23 °C. Finally, a 20 μL of Detection Reagent was added to each well, incubated for 10 min at 23 °C, and luminescence was measured on a SpectraMax-i3 Plate reader (Molecular Devices) with 300 ms read time. To perform GAP-stimulated GTP hydrolysis or GEF activity assays, the range of H-Ras protein concentrations (0, 0.02, 0.04, 0.08, 0.16, 0.31, 0.63, 1.25, 2.5, 5, 10, and 20 μM) were prepared, and the reaction was performed with the addition of 1 μM GAP domain of RASA1 (GAP-334; residues 714–1047) or catalytic domain of Son of Sevenless (SOS^{cat}; residues 564–1049). All experiments were repeated three times. For the determination of EC_{50} values, concentrations of H-Ras proteins were plotted against relative fluorescence units of the average of three independent experiments. The data were analyzed by Bayesian inference using two-dimensional grid search assuming a sigmoidal curve model (see more detail in the following section "Data Analysis for GTP Hydrolysis Assay").

Data Analysis for GTP Hydrolysis Assay

The noise level as a function of the luminescence at each data point was estimated assuming the following model:

$$\sigma(F) = \sqrt{{\sigma_{\mathrm{A}}}^2 + (\sigma_{\mathrm{R}} F)^2} \qquad \text{(S1)}$$

where $\sigma(F)$ is a standard deviation of the noise for the data point with the true luminescence F, σ_{A} is a constant representing the absolute noise which mainly is derived from the luminescence readout, and σ_{R} is a constant representing the relative noise which mainly is derived from the liquid handling. The constants were estimated using the duplicate ($N = 2$) experiments for each data point as follows:

$$\sigma_{\mathrm{A}} = \sqrt{\frac{1}{M(N-1)} \sum_{m=1}^{M} \sum_{n=1}^{N} (F_{mn} - \hat{\mu}_m)^2} \qquad \text{(S2)}$$

$$\sigma_{\mathrm{R}} = \sqrt{\frac{1}{M(N-1)} \sum_{m=1}^{M} \sum_{n=1}^{N} \left(\frac{F_{mn} - \hat{\mu}_m}{\hat{\mu}_m}\right)^2} \qquad \text{(S3)}$$

where $m = 1,2, \dots M$ is a data point index, F_{mn} is an observed luminescence, and $\hat{\mu}_m = \frac{1}{N}\sum_n X_{mn}$ is an estimated true luminescence for the data point m. The data points with $\hat{\mu}_m < 1{,}000\ \mathrm{a.u.}$ and $\hat{\mu}_m > 10{,}000\ \mathrm{a.u.}$ were used for the estimation of σ_{A} and σ_{R}, respectively. The estimated values of σ_{A} and σ_{R} were 76.35 a.u. and 0.0391, respectively.

The hydrolysis reaction rate is quite complicated and time-dependent because it depends on the catalysis rate, association/dissociation rates of nucleotides, and concentration of nucleotides. Therefore, instead of a model based on the chemical reaction kinetics, we adopted the following logistic function, which is the same form as Hill equation, as a phenomenological model:

$$f(x; \boldsymbol{\theta}) = \frac{A}{1 + \left(\frac{x}{B}\right)^C} \qquad \text{(S4)}$$

where x is the Ras concentration, $f(x)$ is the luminescence, and $\boldsymbol{\theta} = \{A, B, C\}$ is the explanatory variable. In particular, B represents the apparent hydrolysis activity, corresponding to the Ras concentration that hydrolyzes a half of the total GTP. Assuming the Gaussian noise, the log likelihood function is

$$L(\boldsymbol{\theta}) = -\frac{1}{2} \sum_i \left(\frac{y_i - f(x_i; \boldsymbol{\theta})}{\sigma(y_i)}\right)^2 \qquad \text{(S5)}$$

where i is the data index, and y_i is the observed luminescence. Assuming the uniform prior distribution, we calculated the posterior distribution of $\boldsymbol{\theta}$ for each experimental condition by a three-dimensional grid search. Further local optimization using "lsqnonlin" function of MATLAB R2020a software (MathWorks) was performed to obtain maximum-a-posteriori (MAP) estimator $\widehat{\boldsymbol{\theta}} = \underset{\boldsymbol{\theta}}{\operatorname{argmax}}\, L(\boldsymbol{\theta})$. In short, we fitted data to Eq. S4 in a Bayesian manner. The MAP estimator is the least-squares solution.

Real-Time NMR Nucleotide Exchange Assay

A 0.2 mM GDP-loaded ^{15}N-labeled H-Ras WT or ^{15}N-labeled ^{19}F-Y32 H-Ras WT proteins was mixed with a 10-fold molar excess of GTPγS (Guanosine 5'-*O*-(3-thiotriphosphate) tetralithium salt; Merck) in a 4 mm NMR tube. Sequential ^{1}H-^{15}N SOFAST-HMQC experiments[9], each spectrum was acquired in 5 min, were collected throughout the time course of the exchange reaction. To calculate the nucleotide exchange rate, the peak intensities of the selected cross-peaks were extracted from individual spectrum and plotted against time. The data were analyzed by Bayesian inference using two-dimensional grid search assuming an exponential association model (see more detail in the following section "Data analysis for Real-Time NMR Nucleotide Exchange Assay"). GEF-mediated nucleotide exchange assays were performed in the same manner, with the addition of SOS^{cat} at a molar ratio of 1:12,000 to each H-Ras protein.

Data Analysis for Real-Time NMR Nucleotide Exchange Assay

The total of 11 (G13, S17, A18. T35. E37, T58, L79, C118, T148, Y157, and R164) and 19 (G10, G13, S17, A18, I21, Q25, V29, T35, E37, D54, M72, F90, C118, T148, Y157, R164, and three unassigned) isolated cross peaks on the ^{1}H-^{15}N SOFAST-HMQC spectra were selected for the H-Ras WT and ^{19}F-Y32 H-Ras WT, respectively, as the cross peaks of GDP-bound form which were not overlapped with the cross peaks of GTPγS-bound form. The total of 2 (Q95 and S106) and 3 (Q95, S106, and one unassigned) isolated cross peaks were selected for the H-Ras WT and ^{19}F-Y32 H-Ras WT, respectively, as the signals of GTPγS-bound form which were not overlapped with the signals of GDP-bound form. Assuming the Gaussian noise, the log likelihood function is

$$L(\boldsymbol{\theta}) = -\frac{1}{2\sigma^2}\left\{\sum_i \left(\min_{A_{\mathrm{GDP},i}} I_{\mathrm{GDP},i}(t) - f_{\mathrm{GDP}}\left(t; T, C, A_{\mathrm{GDP},i}\right)\right)^2 + \sum_i \left(\min_{A_{\mathrm{GTP\gamma S},i}} I_{\mathrm{GTP\gamma S},i}(t) - f_{\mathrm{GTP\gamma S}}\left(t; T, A_{\mathrm{GTP\gamma S},i}\right)\right)^2\right\} \quad \text{(S6)}$$

where $\boldsymbol{\theta} = \{T, C\}$ is the explanatory parameter, σ is the standard deviation of the noise evaluated using the spectral region without cross peaks (-2 to 2 ppm and 11 to 12 ppm), and $I_{\mathrm{GDP},i}(t)$ and $I_{\mathrm{GTP\gamma S},i}(t)$ are the i-th cross-peak intensities as the functions of time from the start of the NMR measurement. f_{GDP} and $f_{\mathrm{GTP\gamma S}}$ are the phenomenological model functions assuming that both are single exponential sharing the same time constant T:

$$f_{\mathrm{GDP}}(t; T, C, A) = A\left((1 - C)\exp\left(-\frac{t}{T}\right) + C\right) \quad \text{(S7)}$$

$$f_{\mathrm{GTP\gamma S}}(t; T, A) = A\left(1 - \exp\left(-\frac{t}{T}\right)\right) \quad \text{(S8)}$$

Assuming the uniform prior distribution, we calculated the posterior distribution of $\boldsymbol{\theta}$ for each experimental condition by a two-dimensional grid search. Further local optimization using "lsqcurvefit" function of MATLAB R2020a software (MathWorks) was performed to obtain MAP estimator $\widehat{\boldsymbol{\theta}} = \underset{\boldsymbol{\theta}}{\operatorname{argmax}} L(\boldsymbol{\theta})$. In short, we fitted data to Eq. S7 and S8 in a Bayesian manner. The MAP estimator is the least-squares solution.

Effector Binding Assay

The effector binding assays were performed using a Biacore T200 (Cytiva). An anti-GST antibody was immobilized on CM5 chip (Cytiva) by amine coupling following the manufacture's recommended protocol. The fusion proteins of GST and the Raf1 RBD and RGL RBD (GST-Raf1 RBD and GST-RGL RBD, respectively) were immobilized on anti-GST antibody-coated CM5 chip in TBS-T buffer (20 mM Tris-Cl (pH 7.4) containing 150 mM NaCl, and 0.05% (v/v) Tween 20) containing 5 mM $MgCl_2$ and 10 μM GMPPNP (TBS-T* buffer) using a constant flow rate of 5 μL/min to achieve the response level at 180 or 90 RUs for GST-Raf1 RBD or GST-RGL RBD, respectively. For the interaction of the H-Ras protein with the GST-Raf1 RBD, the multi-cycle kinetics analysis was performed. GMPPNP-loaded H-Ras proteins were flowed across the surface of the chip at a constant flow rate of 50 μL/min for 120 s (contact time), then dissociated from the GST-Raf1 RBD surface for 120 s (dissociation time) by flowing TBS-T* buffer across the surface of the chip. All H-Ras proteins were measured at a range of concentrations (0, 1.56, 3.13, 6.25, 12.5, 25, and 50 nM). A 50 nM GDP-loaded H-Ras proteins were also tested in the same way, except using TBS-T* buffer without GMPPNP as a negative control. For the binding of the H-Ras protein to the GST-RGL RBD, the steady-state affinity analysis was performed in the similar way of the interaction with the GST-Raf1 RBD, except using 30 μL/min of a constant flow rate, and 180 s for the contact and dissociation times. H-Ras WT, and ^{19}F-Y96 and ^{19}F-Y157 H-Ras WT proteins were measured at a range of concentrations (0, 0.117, 0.469, 0.938, 1.875, 3.75, 7.5, 15, and 30 μM), while ^{19}F-Y32 H-Ras WT protein was tested at a range of concentrations (0, 1.17, 2.349, 4.69, 9.38, 18.75, 37.5, 75, 150, and 300 μM).

Subcellular Localization of the Fluorescent Labeled H-Ras with Lysosome

To monitor the subcellular localization of H-Ras protein with lysosome, a CytoPainter Lysosomal Staining Kit (Abcam) were used for an organelle marker. A total of 1.25×10^6 HeLa cells were electropolated with a 0.1 mL solution of 0.7 mM Alexa-488 labeled H-Ras protein. After 21 h incubation of the electropolated HeLa cells at 37 °C under a 5% CO_2 humidified atmosphere in high-glucose Dulbecco's Modified Eagle Medium (DMEM; Thermo Fisher Scientific), supplemented with 10% Fetal Bovine Serum (FBS; Thermo Fisher Scientific), 200 U/mL penicillin (PCN; Nacalai Tesque), and 200 μg/mL streptomycin (STR; Nacalai Tesque), the DMEM was removed from the dish, followed by rinse on the cell surface with Hanks' Balanced Salt Solution (HBSS). Subsequently, the LysoRed Indicator solution was added, and the cells were incubated at 37 °C for 30 minutes under a 5% CO_2 humidified atmosphere. After rinse on the cell surface three times with HBSS, DMEM without phenol red (Nacalai Tesque), supplemented with 10% FBS, 200 U/mL PCN and 200 μg/mL STR was refiled on the dish. For nuclear staining, 2–3 drops of NucBlue® Live ReadyProbes® Reagent (Hoechst 33342; Thermo Fisher Scientific) were added to the media.

Immunofluorescence Microscopy of the FLAG-H-Ras and Lysosome

A total of 1.25×10^6 HeLa cells were electroporated with a 0.1 mL of 0.7 mM FLAG-tag-containing H-Ras (FLAG-H-Ras) protein. Subsequently, 0.4×10^6 electropolated HeLa

cells were seeded on an 18 × 18 mm glass coverslip (MATSUNAMI, Thickness No.1) and incubated for 22 h at 37 °C under a 5% CO_2 humidified atmosphere in DMEM. The cell surface was rinsed with D-PBS (Nacalai Tesque), and then the cells were fixed by adding a 4% paraformaldehyde Phosphate Buffer Solution (Nacalai Tesque) for 30 min at room temperature (~25 °C). After the cell surface was rinsed with D-PBS (Nacalai Tesque), the cells were permeabilized by adding a 0.3% Triton X-100 in D-PBS (Nacalai Tesque) for 15 min at room temperature (~25 °C). After the cell surface was rinsed with D-PBS (Nacalai Tesque) again, the cells were mixed with a 3% bovine serum albumin (BSA) and a 0.1% Triton X-100 in D-PBS (Nacalai Tesque), and incubated for 30 min at room temperature (~25 °C) to block unspecific binding of the antibodies. After the cell surface was rinsed with D-PBS (Nacalai Tesque) as usual, the cells were incubated for overnight at 4 °C with a mouse monoclonal anti-FLAG M2 antibody (Merck, 1:500 dilution) and in a rabbit monoclonal anti-LAMP1 antibody (Abcam, 1:100 dilution) for lysosome_LAMP1. After the cell surface was rinsed with a 0.1% BSA and a 0.1% Triton X-100 in D-PBS (Nacalai Tesque), the cells were incubated for 30 min at room temperature (~25 °C) with the secondary antibodies, an AlexaFluor488-conjugated goat anti-mouse IgG(H+L) polyclonal antibody (ThermoFisher, 1:200 dilution) and an Alexa Fluor 594-conjugated donkey anti-rabbit IgG(H+L) polyclonal antibody (ThermoFisher, 1:200 dilution). The cell surface was rinsed with D-PBS (Nacalai Tesque), and then the glass coverslip was sealed by mounting a slide glass coated with Prolong Glass Antifade Mountant with NucBlue Stain (ThermoFisher) and stored for overnight at room temperature (~25 °C).

Immunoprecipitation of the FLAG-H-Ras and Western Blotting

A total of 1.25×10^6 HeLa cells were electroporated with a 0.1 mL of 1 mM FLAG-H-Ras protein and incubated for 22 h at 37 °C under a 5% CO_2 humidified atmosphere in DMEM. Subsequently, the cells were detached from the dish by Accumax (Innovative Cell Technologies) and separated from the supernatant by centrifugation. The cells were mildly solubilized by suspension in 100 μL of TBS buffer (50 mM Tris-Cl (pH 7.4) containing 150 mM NaCl) containing 10 mM $MgCl_2$ and 1% CHAPS with sonication for 5 min. The solubilized fraction was separated from the precipitate by centrifugation and applied to a micro-spin column filled with Anti-FLAG® M2 Affinity Gel (Merck). In this step, the solubilized fraction was diluted by TBS buffer to reduce the detergent concentration to less than 0.1%. The reaction mixture was gently mixed at 4 °C for 4 h, and then other proteins that did not bind to the M2 antibody (unbound fraction) were removed by centrifugation. The M2 antibody was washed 3 times with 2.5 column volumes of TBS buffer. Subsequently, 150 μg/mL of FLAG-peptide was applied to the micro-spin column and gently mixed with the M2 antibody at 4 °C for 1 h. Finally, the FLAG-H-Ras protein was eluted by centrifugation of the micro-spin column.

The M2 antibody-unbound fraction and the eluted fraction from the micro-spin column, which should contain the FLAG-H-Ras protein, were loaded onto a 15% SDS-PAGE gel (DRC), and then transferred onto a PVDF membrane by a Trans-Blot Turbo Transfer System (Bio-Rad). After blocking, the membrane was probed with an anti-FLAG M2 antibody (Merck, 1:2,000 dilution). The secondary antibody was anti-mouse IgG antibody-HRP (Promega, 1:1,000 dilution). The blocking and antibody reactions were performed using an iBind Western

System (Novex). The membrane was developed using the ECL Prime reagent (Cytiva). Luminescence signals were detected with an ImageQuant LAS4000 imager (Cytiva).

LC-MS/MS Analysis

The purified FLAG-H-Ras protein was dissolved in 50 mM NH_4HCO_3 by buffer exchange, and then CH_3CN was added to the FLAG-H-Ras protein solution to 10% v/v. After adding DTT, the FLAG-H-Ras protein solution was incubated at 56 °C for 30 min, and then allowed to cool to room temperature (~25 °C). Subsequently, the FLAG-H-Ras protein solution was incubated at 37 °C for 30 min in the dark, after adding iodoacetamide (Merck). The FLAG-H-Ras protein was digested with AspN (WAKO) at 37 °C overnight. The digested peptides were desalted and concentrated with a StageTip C8 (Thermo Fisher Scientific).

The LC-MS/MS analysis was performed using an EASY-nLC 1000 (Thermo Fisher Scientific) and a Q Exactive mass spectrometer (Thermo Fisher Scientific) equipped with a nanospray ion source. The peptides were separated with a NANO-HPLC capillary column C18 (0.075 x 150 mm, 3 μm, Nikkyo Technos) using a 60 min gradient at a flow rate of 300 nL/min: 5–35% B in 48 min, and then 35–65% B in 12 min (solvent A: 0.1% formic acid; solvent B: 0.1% formic acid in acetonitrile). The resulting MS and MS/MS data were searched against the Swiss-Prot database, using Proteome Discoverer (Thermo Fisher Scientific) with the MASCOT search engine software (Matrix Science). These analyses were performed at the Support Unit for Bio-Material Analysis in the RIKEN Center for Brain Science (CBS), Research Resources Division (RRD).

Liposome Preparation

Small unilamellar vesicles (SUVs) composed of 1,2-dioleoyl-*sn*-glycero-3-phosphocholine (DOPC; Avanti) and 1,2-dioleoyl-*sn*-glycero-3-phospho-(1'-*rac*-glycerol) (DOPG; Avanti) at a molar ratio of 3.3:1 DOPC/DOPG were prepared. A 15 mg of DOPC and a 4.5 mg of DOPG were well mixed and completely dissolved in 300 μL of chloroform. The lipids were dried in an oxygen-free nitrogen stream, followed by at least 2 h under vacuum in a vacuum desiccator. The dried lipids were resuspended in EPB containing 10 mM $MgCl_2$ and 1 mM DTT, and subjected to bath sonication to achieve a 2% (w/v) vesicle solution. The vesicle solution was diluted with the same buffer to achieve a 0.5% (w/v) after removing insoluble particles by centrifugation, and then, further freeze-thawed in liquid N_2 and subjected to bath sonication for 15 min. This freeze-thaw and sonication cycle was repeated in 5 times to achieve SUVs with diameters ~50 nm as determined by dynamic light scattering (DLS).

2. Supporting Figures

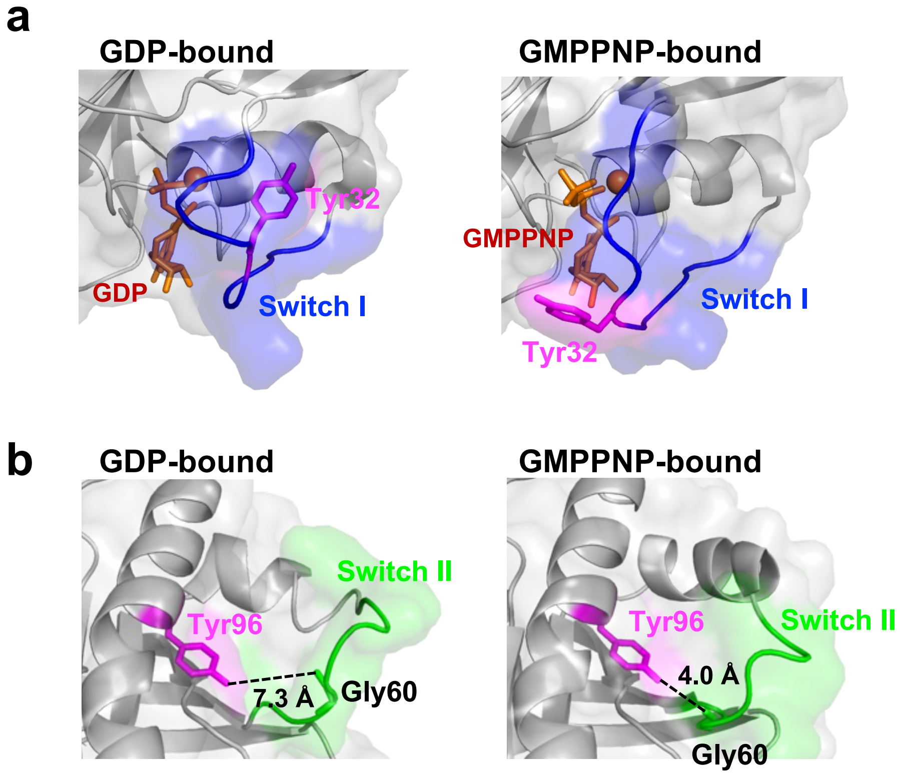

Figure S1. The local structural differences around the OCF_3Phe incorporation sites between the GDP- (PDB code: 4Q21) and GMPPNP-bound (PDB code: 5P21) forms of H-Ras. (a) The orientation difference of Tyr32. (b) The relative orientations of Tyr96 with respect to switch II.

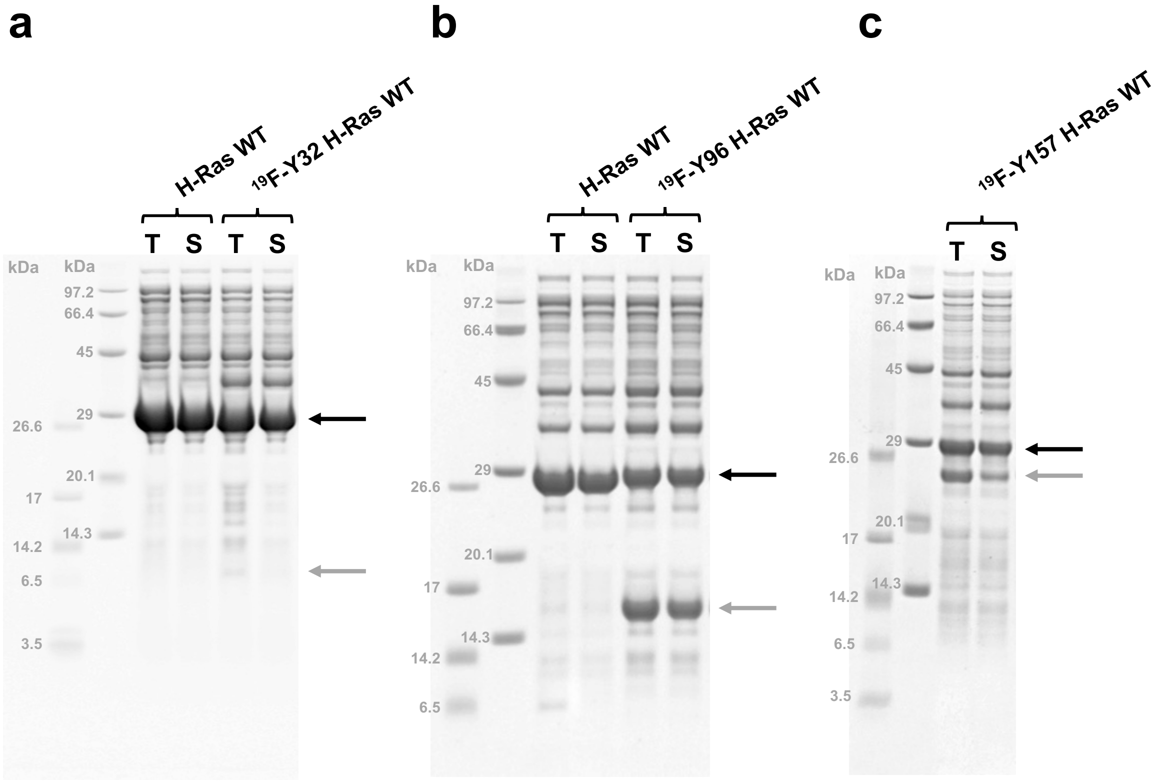

Figure S2. Cell-free expression of the ^{19}F-Y32 (a), ^{19}F-Y96 (b), and ^{19}F-Y157 H-Ras WT proteins (c). The expression level of each ^{19}F-labeled H-Ras protein is compared with that of the H-Ras WT. 15% SDS-PAGE gel, with two different types of molecular-weight size markers, visualized by Coomassie Brilliant Blue staining. T and S indicate the total expression and the soluble fractions, respectively. The expected band sizes of the full-length proteins are indicated by the black arrows. The gray arrows show the expected band sizes of the prematurely terminated products. Judging from the band widths of the full-length proteins and the prematurely terminated products, the efficiencies of OCF_3Phe incorporation into H-Ras protein were estimated to be ~100%, ~50%, and ~70% for the ^{19}F-Y32, ^{19}F-Y96, and ^{19}F-Y157 H-Ras WT, respectively.

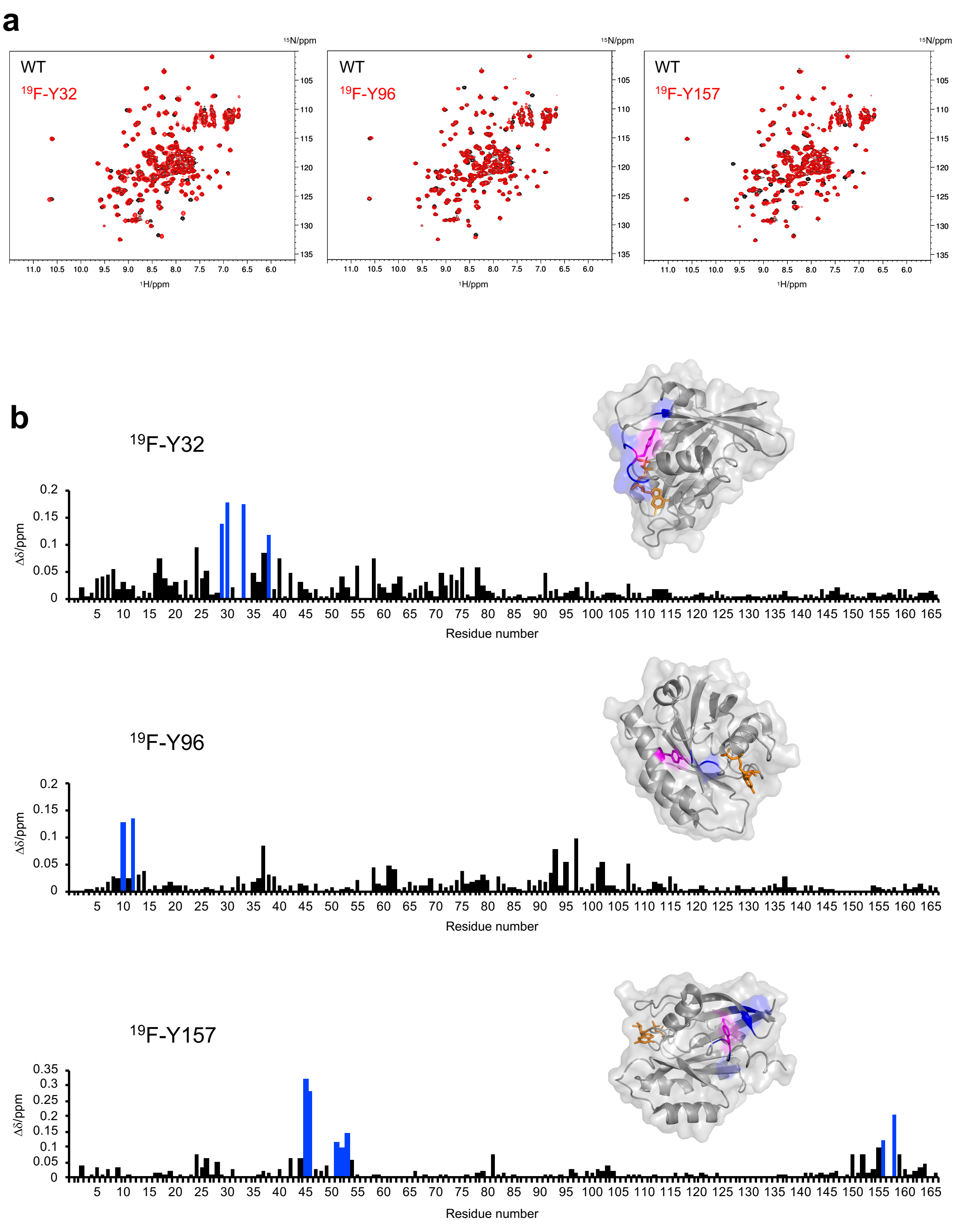

Figure S3. NMR characterization of the ^{19}F-labeled H-Ras. (a) Superimposition of the ^{1}H-^{15}N SOFAST-HMQC spectra of the ^{19}F-Y32, ^{19}F-Y96, and ^{19}F-Y157 H-Ras WT (shown in red) with corresponding the H-Ras WT spectrum (shown in black). Each spectrum represents the GDP-bound form. (b) The normalized chemical shift perturbations (CSPs; Δδ) induced to the backbone amide signals between the H-Ras WT and each ^{19}F-labeled H-Ras WT. The Δδ values were calculated using $\{(\Delta\delta^{1}H)^2 + (0.2\Delta\delta^{15}N)^2\}^{1/2}$. The residues with $\Delta\delta > 0.1$ ppm are shown in blue bars and mapped on the crystal structure (PDB code: 4Q21) with indicating the OCF_3Phe incorporated sites colored in magenta. GDP are shown by orange sticks. The large CSPs are observed only for the resonances in the vicinity of each OCF_3Phe incorporated site. The resonance assignments were transferred from the previous studies[2, 3].

a

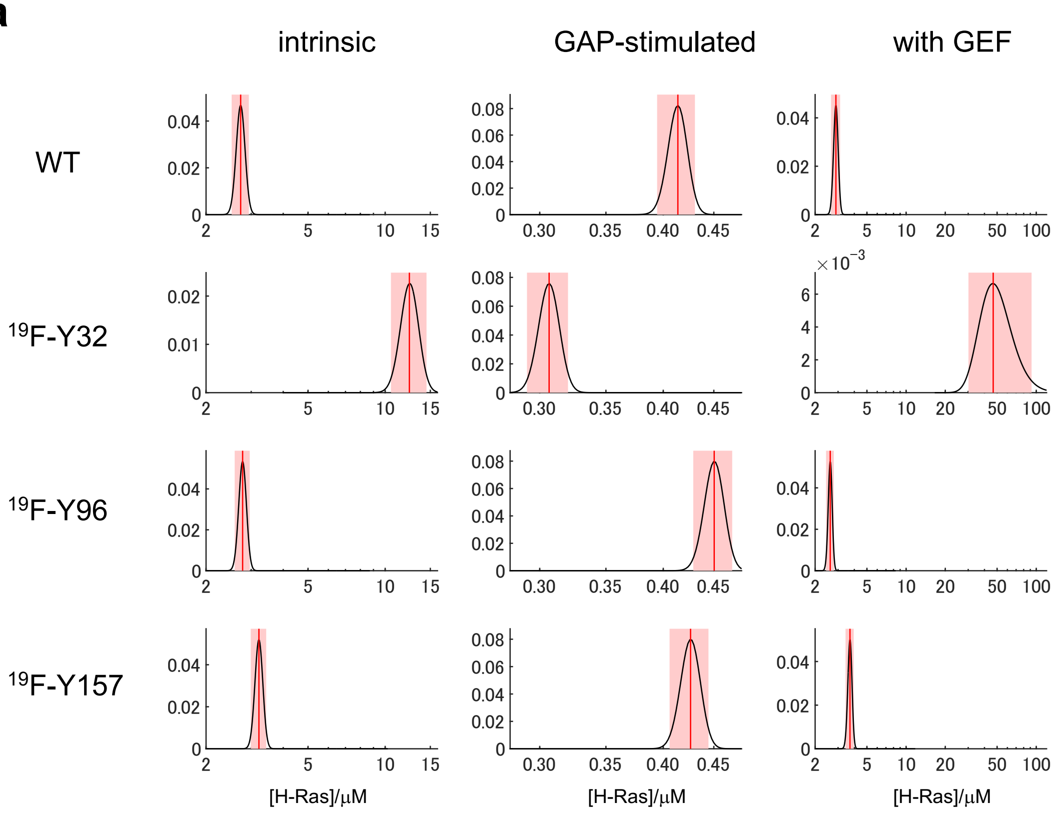

b

	EC$_{50}$/μM		
	intrinsic	GAP-stimulated	with GEF
WT	2.73 (2.52–2.94)	0.414 (0.394–0.431)	2.89 (2.65–3.11)
^{19}F-Y32	12.43 (10.53–14.50)	0.307 (0.291–0.320)	46.68 (30.12–92.05)
^{19}F-Y96	2.78 (2.59–2.96)	0.450 (0.429–0.470)	2.61 (2.43–2.78)
^{19}F-Y157	3.22 (2.99–3.43)	0.426 (0.406–0.444)	3.70 (3.42–3.96)

The values in the brackets show 95% credible intervals.

Figure S4. GTP hydrolysis assays using Promega GTPase-Glo system of the H-Ras WT and ^{19}F-Y32, ^{19}F-Y96, and ^{19}F-Y157 H-Ras WT proteins. (a) The posterior distributions of the H-Ras concentrations at EC$_{50}$ values calculated by Bayesian inference using a grid search. The results of intrinsic and GAP-stimulated GTP hydrolysis assays as well as GEF assay are shown for the H-Ras WT and ^{19}F-Y32, ^{9}F-Y96, and ^{9}F-Y157 H-Ras WT proteins. The maximum-a-posteriori (MAP) estimators and 95% credible intervals of the H-Ras concentrations at EC$_{50}$ values are indicated by red solid lines and red boxes, respectively, and summarized in the bottom table (b).

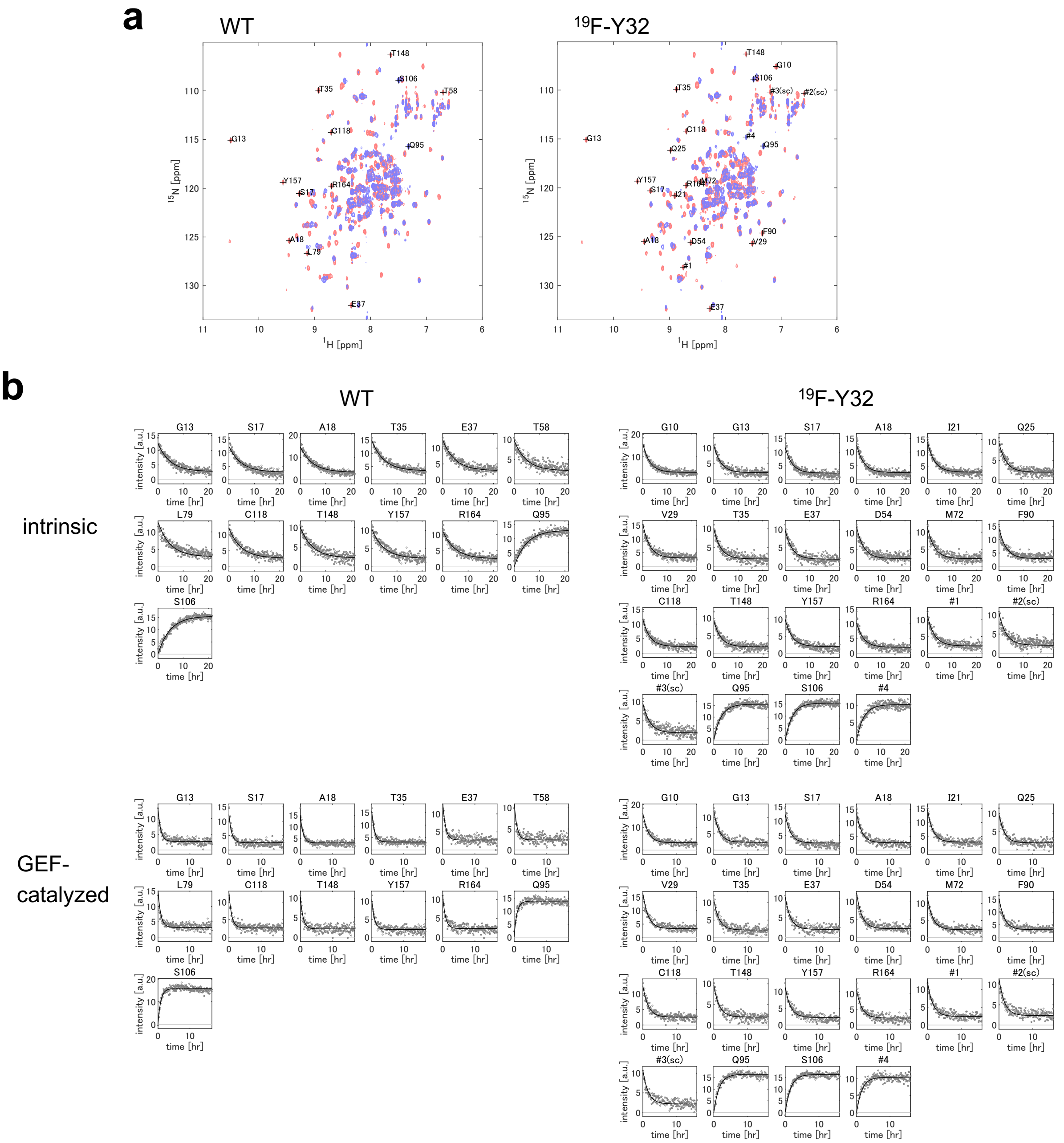

c

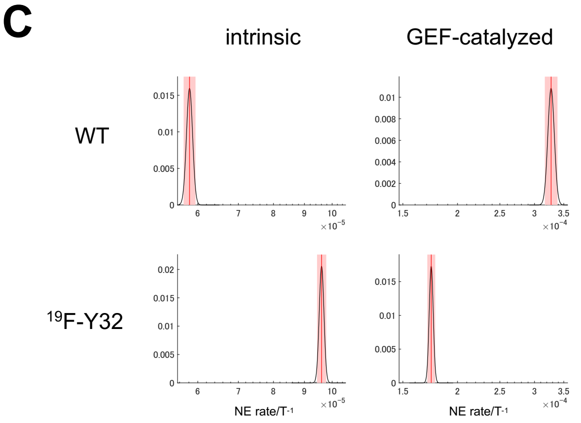

	intrinsic	
	NE rate/×10^{-5} s^{-1}	relative NE rate
WT	5.81 (5.69–5.95)	1
^{19}F-Y32	9.61 (9.44–9.78)	1.65 (1.61–1.70)

	GEF-catalyzed	
	NE rate/×10^{-5} s^{-1}	relative NE rate
WT	32.7 (31.7–33.9)	1
^{19}F-Y32	17.4 (17.1–17.8)	0.532 (0.511–0.553)

The values in the brackets show 95% credible intervals.

Figure S5. Intrinsic and GEF-catalyzed nucleotide-exchange (NE) assays for the H-Ras WT and ^{19}F-Y32 H-Ras WT proteins. (a) Superimposition of the ^{1}H-^{15}N SOFAST-HMQC spectra of the H-Ras WT and ^{19}F-Y32 H-Ras WT proteins at the first time point, corresponding to the GDP-bound form (shown in red) with those at the last time point, reflecting the GTPγS-bound form (shown in blue). The selected peaks to use to determine the NE rates are labeled. (b) Real-time NE curves of the H-Ras WT and ^{19}F-Y32 H-Ras WT proteins. Intensities of the selected peaks are plotted versus time. Most peaks were selected from the GDP-bound form, except for the peaks of Q95 and S106 in the H-Ras WT protein, and those of Q95, S106, and an unassigned peak in the ^{19}F-Y32 H-Ras WT protein, which were picked from the GTPγS-bound form. The straight lines represent Eqs.S7 or S8 (in the SI Experimental Section) fitted to the experimental data. (c) The posterior distributions of NE rates of the H-Ras WT and ^{19}F-Y32 H-Ras WT proteins calculated by Bayesian inference using a grid search. The maximum-a-posteriori (MAP) estimators and the 95% credible intervals of the NE rates are indicated by red solid lines and red boxes, respectively, and summarized in the tables. The relative NE rates of the ^{19}F-Y32 H-Ras WT protein referred to those of the H-Ras WT protein are also indicated.

a

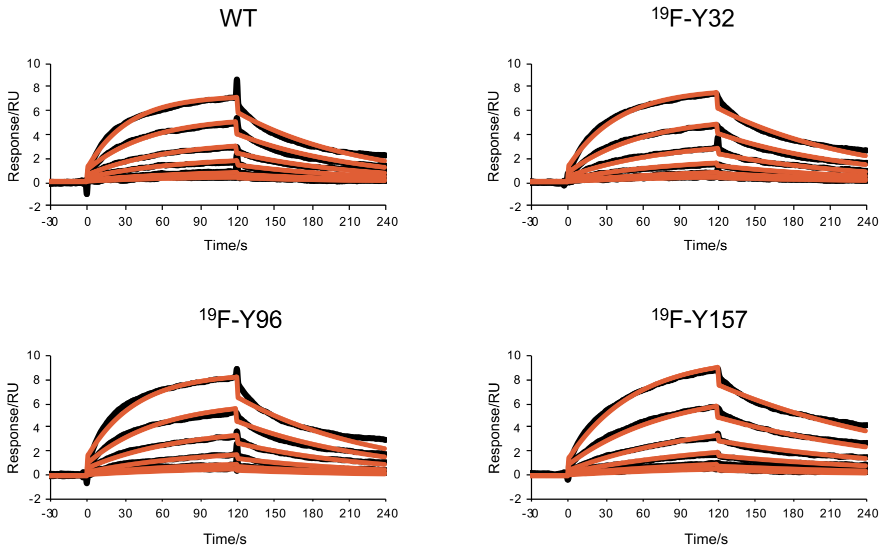

b

	k_{on}/Ms^{-1}	k_{off}/1s^{-1}	K_D/M
WT	3.901 x 10^5	1.023 x 10^{-2}	2.621 x 10^{-8}
^{19}F-Y32	3.184 x 10^5	0.883 x 10^{-2}	2.774 x 10^{-8}
^{19}F-Y96	3.311 x 10^5	0.902 x 10^{-2}	2.724 x 10^{-8}
^{19}F-Y157	2.533 x 10^5	0.598 x 10^{-2}	2.361 x 10^{-8}

Figure S6. Binding kinetic assays of the H-Ras WT and ^{19}F-Y32, ^{19}F-Y96, and ^{19}F-Y157 H-Ras WT proteins to Raf1 Ras-binding domain (RBD) by SPR. (a) SPR sensorgrams of binding curves for each H-Ras protein binding to GST-Raf1 RBD immobilized on an anti-GST sensor chip (shown in black). The curves corresponding to each H-Ras protein concentrations are 1.56, 3.13, 6.25, 12.5, 25, and 50 nM from the bottom to top. Each sensorgram was analyzed using a 1:1 binding model by Biacore T200 Evaluation Software v1.0. The curve fittings are highlighted in red. The association rates (k_{on}), dissociation rates (k_{off}), and dissociation constants (K_D) of each H-Ras protein for Raf1 RBD are summarized in the bottom table (b).

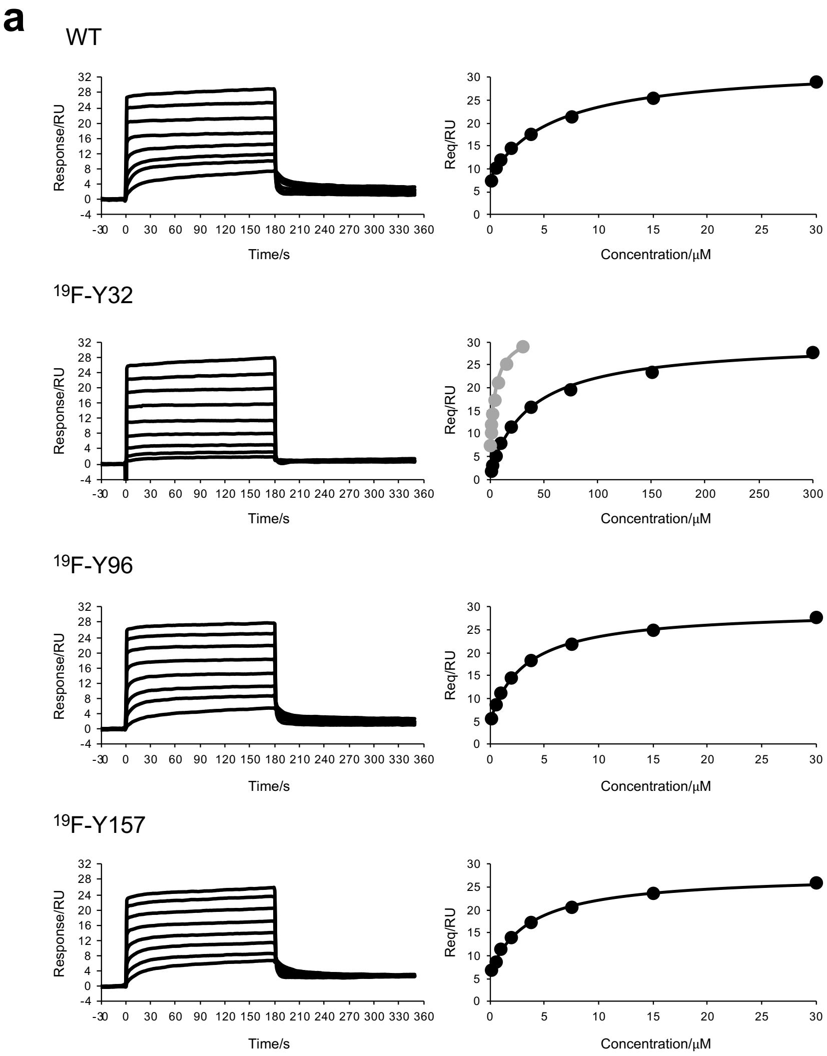

b

	K_D/μM	R_{max}/RU	R^2
WT	5.795	32.55	0.9713
^{19}F-Y32	38.31	30.13	0.9802
^{19}F-Y96	3.161	29.48	0.9799
^{19}F-Y157	3.548	27.75	0.9805

Figure S7. Binding affinity assays of the H-Ras WT and ^{19}F-Y32, ^{19}F-Y96, and ^{19}F-Y157 H-Ras WT proteins to RGL Ras-binding domain (RBD) by SPR. (a) SPR sensorgrams of binding curves for each H-Ras protein binding to GST-RGL RBD immobilized on an anti-GST sensor chip. The curves corresponding to each H-Ras protein concentrations are 0.117, 0.469, 0.938, 1.875, 3.75, 7.5, 15, and 30 μM for the H-Ras WT, ^{19}F-Y96, and ^{19}F-Y157 H-Ras WT, and 1.17, 2.349, 4.69, 9.38, 18.75, 37.5, 75, 150, and 300 μM for the ^{19}F-Y32 H-Ras WT from the bottom to top. Each sensorgram was analyzed using a 1:1 binding model by Biacore T200 Evaluation Software v1.0 and affinity of each H-Ras protein for RGL RBD at the equilibrium were evaluated by plotting of the response units at equilibrium (Req) versus the H-Ras protein concentrations. Each Req was derived from the steady-state plateau of the association phases of each sensorgram. For the ^{19}F-Y32 H-Ras WT protein, the Reqs of the H-Ras WT are also plotted (shown in grey) in the same graph for comparison. The dissociation constants (K_D) of each H-Ras protein for RGL RBD are summarized in the bottom table (b).

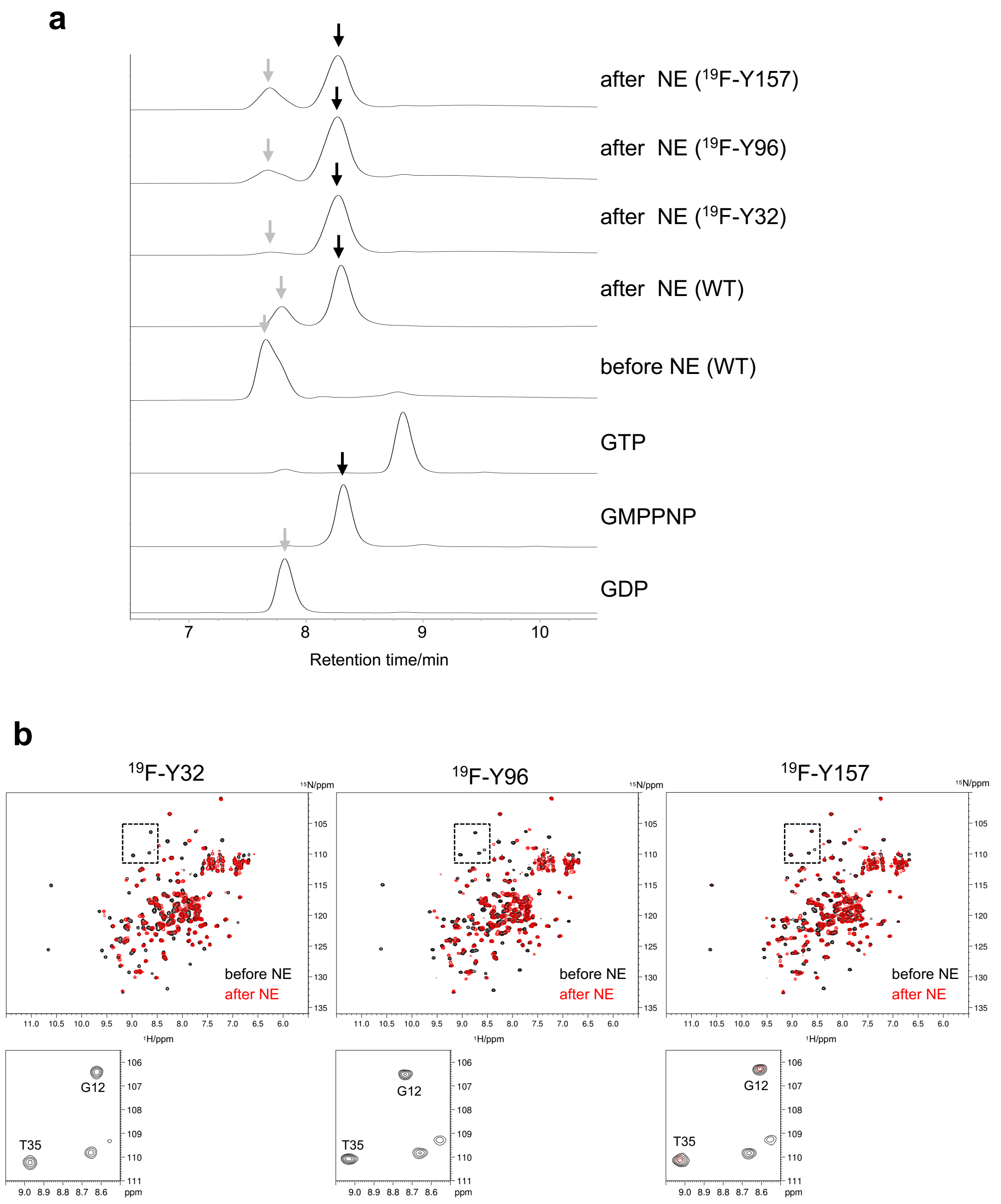

Figure S8. Evaluation of the nucleotide-exchange (NE) procedure from GDP to GMPPNP. (a) Mono Q chromatogram profiles of the before and after NE procedures of the H-Ras WT protein, and the after NE procedures of the ^{19}F-Y32, ^{19}F-Y96, and ^{19}F-Y157 H-Ras WT proteins compared with those of free GDP, free GMPPNP, and free GTP. The peaks corresponding to GDP and GMPPNP are indicated by gray and black arrows, respectively. An intrinsically bound nucleotide to the purified H-Ras protein is GDP. The yields of NE procedures were judged to be ~96%, ~84%, and ~72% for the ^{19}F-Y32, ^{19}F-Y96, and ^{19}F-Y157 H-Ras WT, respectively, by comparing the integrations of the gray and black peak areas. (b) Comparison of the ^{1}H-^{15}N SOFAST-HMQC spectra of the ^{19}F-Y32, ^{19}F-Y96, and ^{19}F-Y157 H-Ras WT proteins before (shown in black) and after (shown in red) NE procedures, which correspond to the GDP- and GMPPNP-bound forms, respectively. The relatively low NE yield of the ^{19}F-Y157 H-Ras WT protein resulted in the appearance of residual GDP-bound signals, exemplified in the resonances of G12 and T35 in the expansions from the dash-boxed areas.

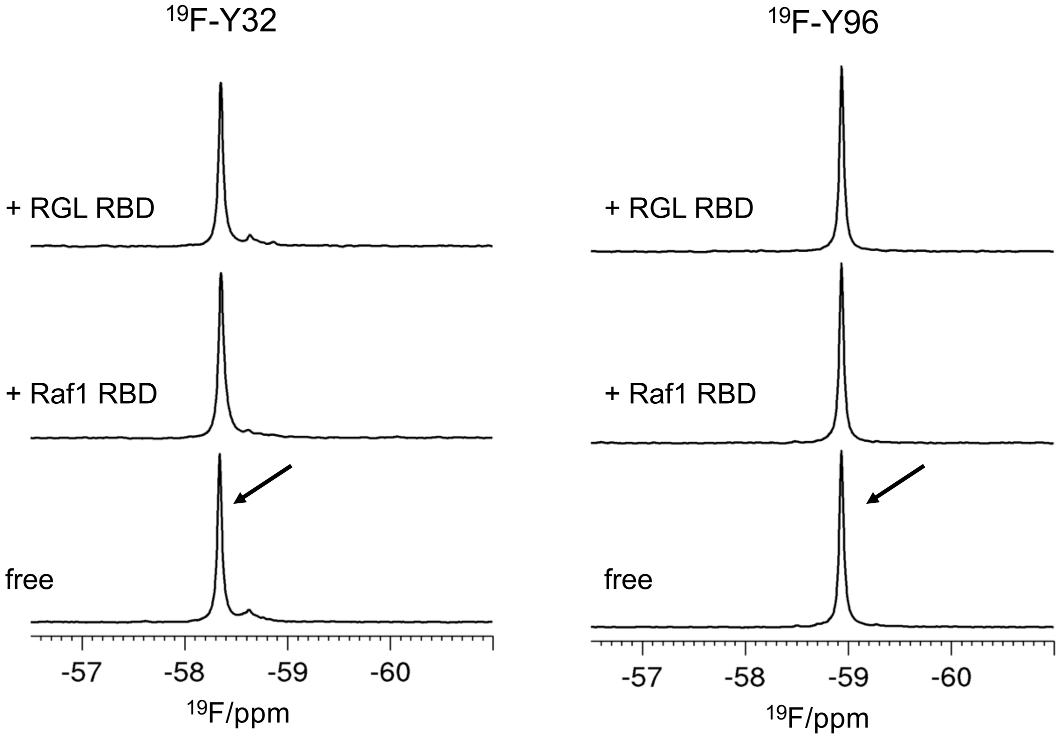

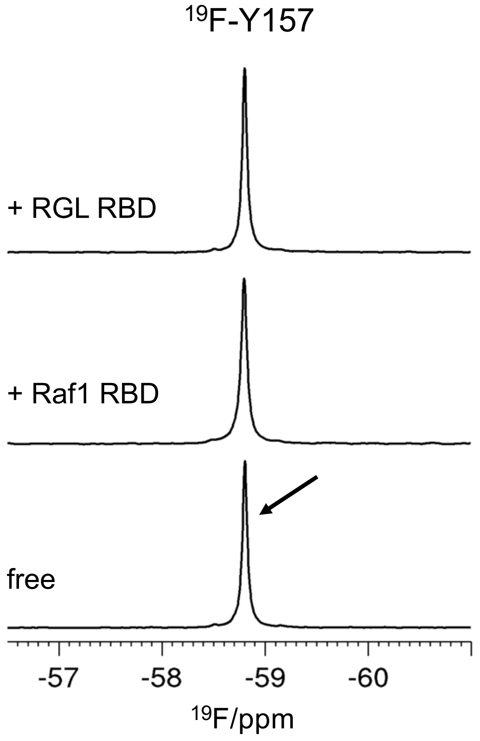

	linewidth/Hz		
	free	+ Raf1 RBD	+ RGL RBD
^{19}F-Y32	28.90	32.43	28.97
^{19}F-Y96	29.14	37.29	30.55
^{19}F-Y157	27.39	27.32	26.69

Figure S9. *In vitro* 1D ^{19}F-NMR spectra of the GDP-bound ^{19}F-Y32, ^{19}F-Y96, and ^{19}F-Y157 H-Ras WT proteins in the absence (free) and presence of Raf1 Ras-binding domain (RBD) and RGL RBD at the molar ratio of 1 to 1. No chemical shift changes were observed for the ^{19}F-NMR signals (shown by arrows) of all ^{19}F-labeled H-Ras WT proteins. The linewidths of each ^{19}F-NMR signal are summarized in the table.

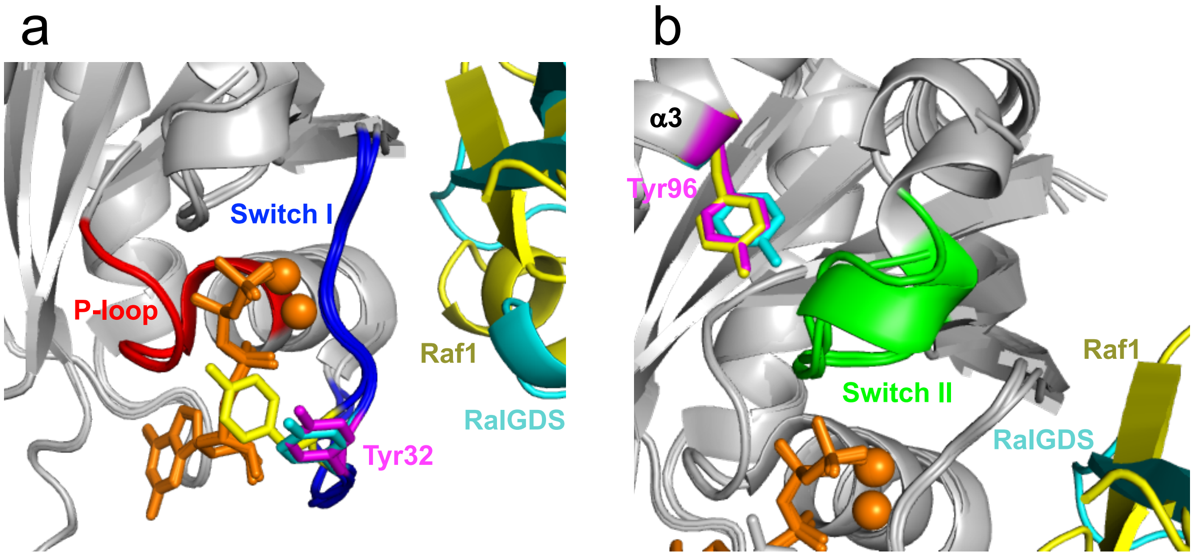

Figure S10. Interactions of H-Ras with the effector-proteins. (a) Superimposition of the GMPPNP-bound H-Ras (PDB code: 5P21) and its complexes with the Raf1 Ras-binding domain (RBD) (PDB code: 4G0N) and the RalGDS RBD (PDB code: 1LFD). The Tyr32 residues are shown as magenta, yellow, and cyan sticks for the free form and in the complexes with the Raf1 RBD and the RalGDS RBD, respectively. The bound Raf1 RBD and RalGDS RBD are colored in yellow and cyan, respectively. The switch I and P-loops are colored in blue and red, respectively. The GMPPNP and magnesium ions are represented by orange sticks and spheres, respectively. The aromatic side chain of Tyr32 interacts with the P-loop in the complex with the Raf1 RBD. (b) The orientation differences of the aromatic side chains of Tyr96. The Tyr96 residues are shown in the same colors as in (a). The aromatic side chain of Tyr96 moves in the complex with the RalGDS RBD, but not in the complex with the Raf1 RBD. The switch II regions are colored in green.

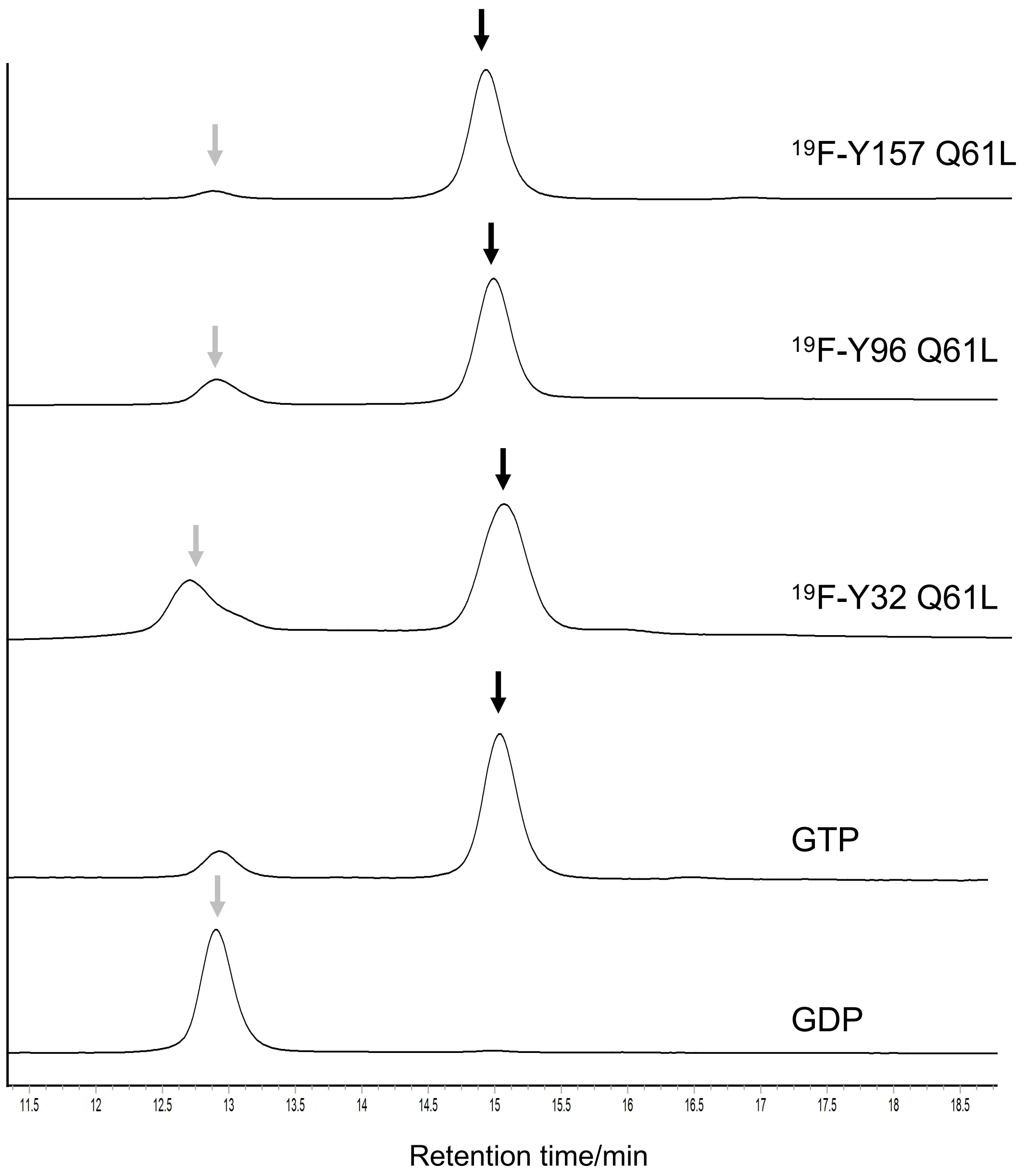

Figure S11. Evaluation of an intrinsically bound nucleotide to the ^{19}F-labeled H-Ras Q61L mutant proteins. Mono Q chromatogram of the purified ^{19}F-Y32, ^{19}F-Y96, and ^{19}F-Y157 H-Ras Q61L mutant proteins are compared with those of free GDP and free GTP. The peaks corresponding to GDP and GTP are indicated by gray and black arrows, respectively. Judging from the peak integrations, the ratios of the intrinsically bound GTP were ~70%, ~90%, and ~98% for the ^{19}F-Y32, ^{19}F-Y96, and ^{19}F-Y157 H-Ras Q61L mutant proteins, respectively.

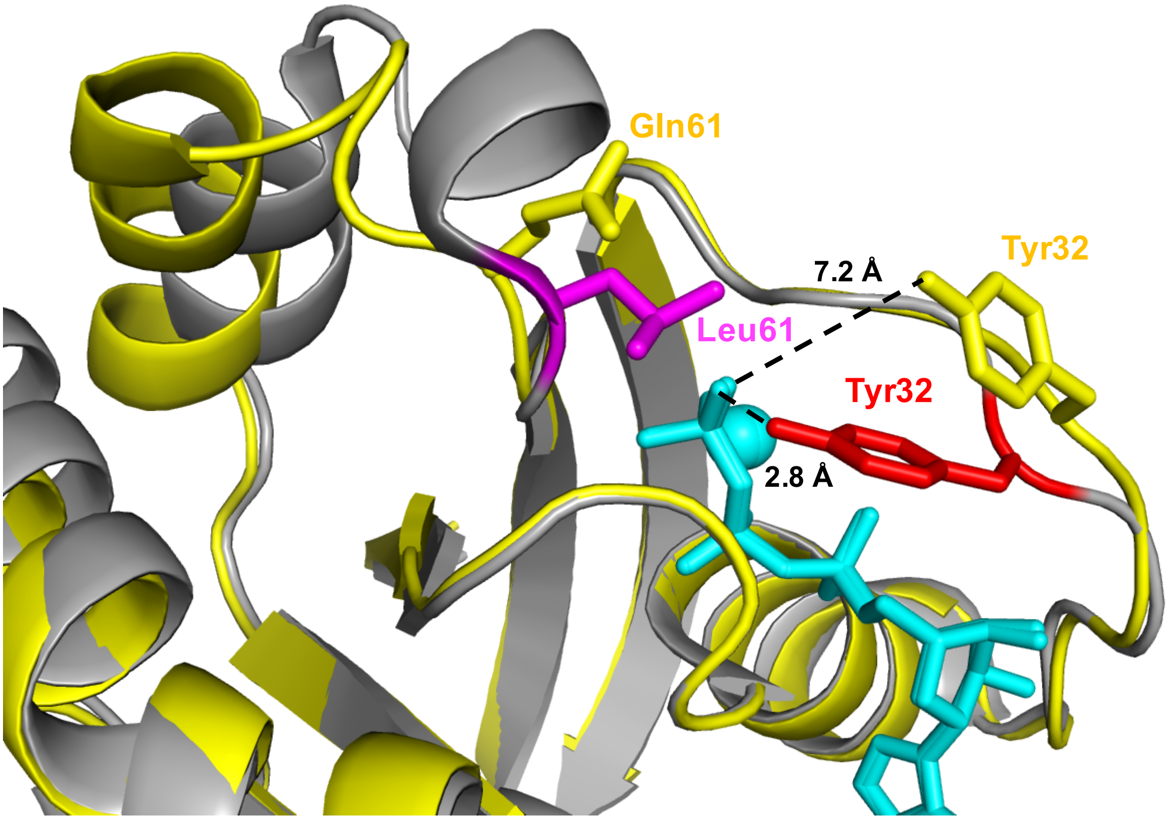

Figure S12. The side chain orientations of Tyr32. In the case of the H-Ras Q61L mutant (grey) (PDB code: 2RGD), the hydroxyl group of Tyr32 (highlighted in red) forms a hydrogen bond with the γ-phosphate of GMPPNP (cyan), whereas that is 7.2 Å away from the γ-phosphate of GMPPNP for the H-Ras WT (yellow) (PDB code: 5P21). Residues Gln61 for the H-Ras WT and Leu61 for the H-Ras Q61L mutant are highlighted as yellow and magenta sticks, respectively.

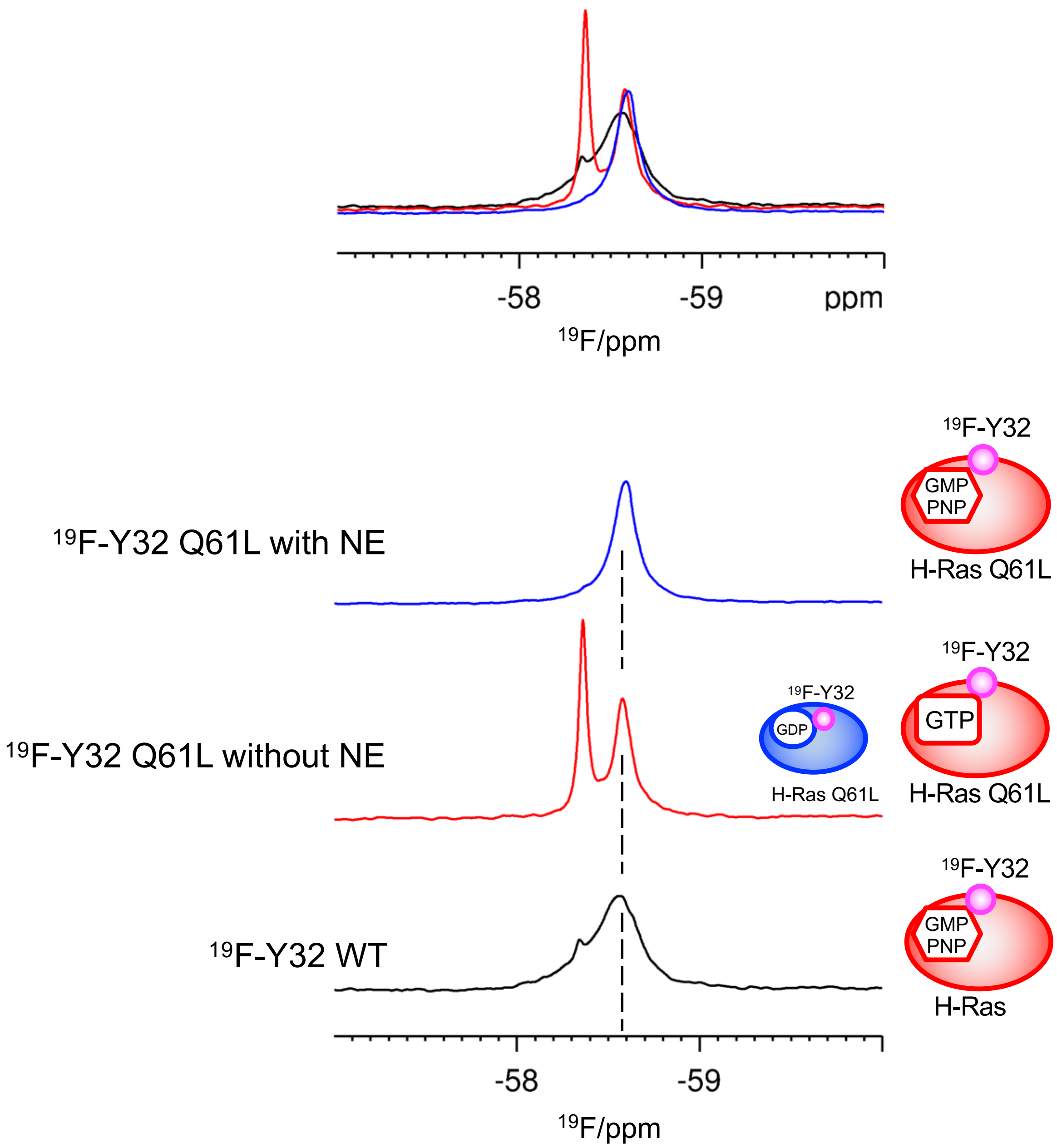

Figure S13. *In vitro* 1D ^{19}F-NMR spectra of the ^{19}F-Y32 H-Ras Q61L mutant with and without NE in comparison with that of the GMPPNP-bound ^{19}F-Y32 H-Ras WT. The GMPPNP-bound signal of the ^{19}F-Y32 H-Ras WT (bottom) and the GTP- and GMPPNP-bound signals of the ^{19}F-Y32 H-Ras Q61L mutant (middle and top, respectively) are shown in dashed line. The expected ^{19}F-NMR signal detections of the ^{19}F-labeled H-Ras are illustrated with cartoon models in each spectrum. These three spectra are overlayed in the same color on the top.

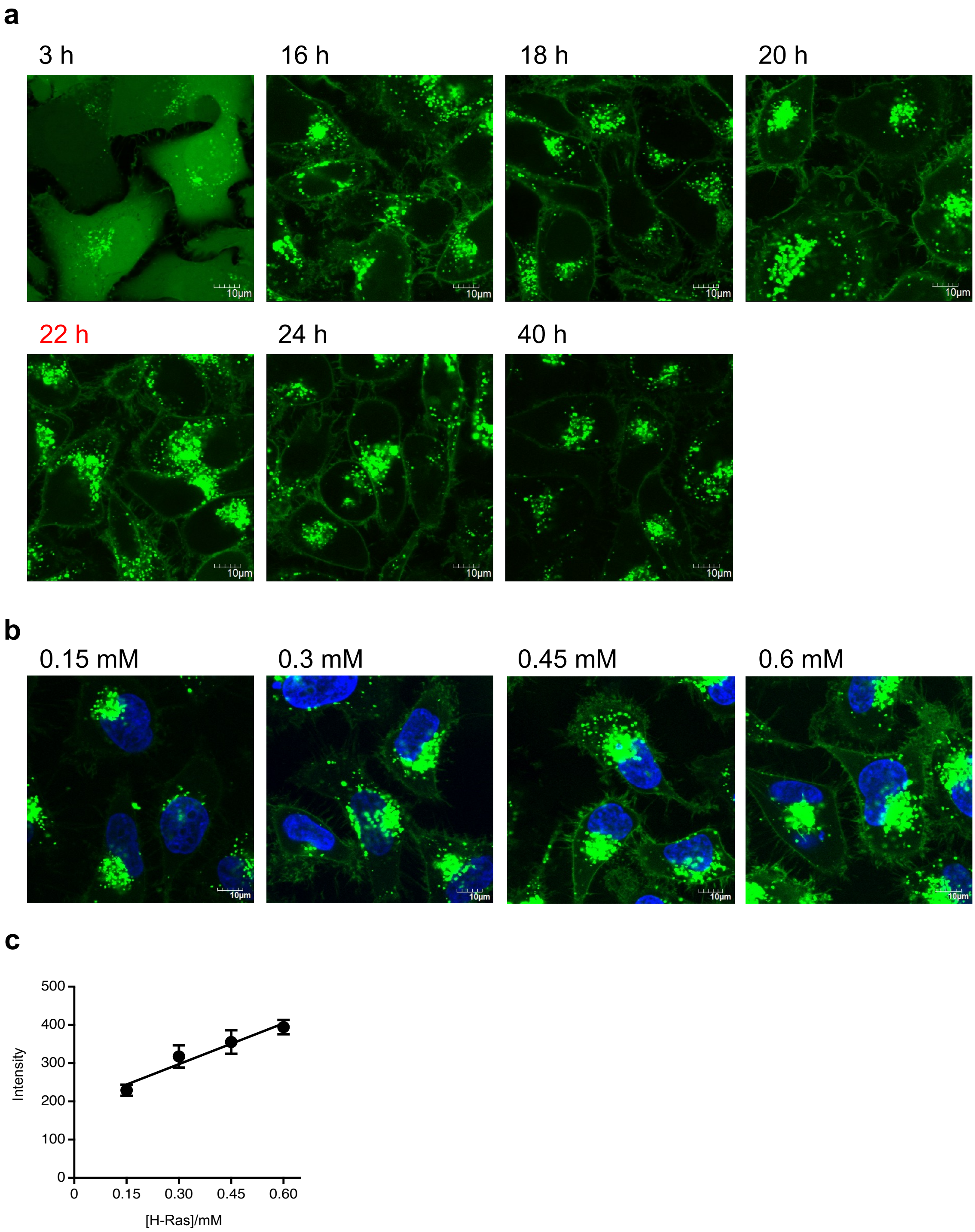

Figure S14. Intracellular distributions of the exogenously delivered GDP-bound H-Ras protein into HeLa cells by electroporation (EP). (a) The confocal microscope images of HeLa cells delivered with the Alexa-488 labeled H-Ras protein, with time evolution. A 0.6 mM H-Ras protein as used for EP. (b) Changes in the plasma membrane (PM) localization of the delivered H-Ras molecules with their delivery amounts. The confocal microscope images of HeLa cells delivered with various amounts of the Alexa-488 labeled H-Ras protein are shown. The images were captured at 22 h after EP. Nuclei were stained with Hoechst 33342. (c) The average fluorescent intensities of six different points at the PM are plotted versus delivered H-Ras protein concentration. Error bars represent the standard deviation of the fluorescent intensities for n = 6.

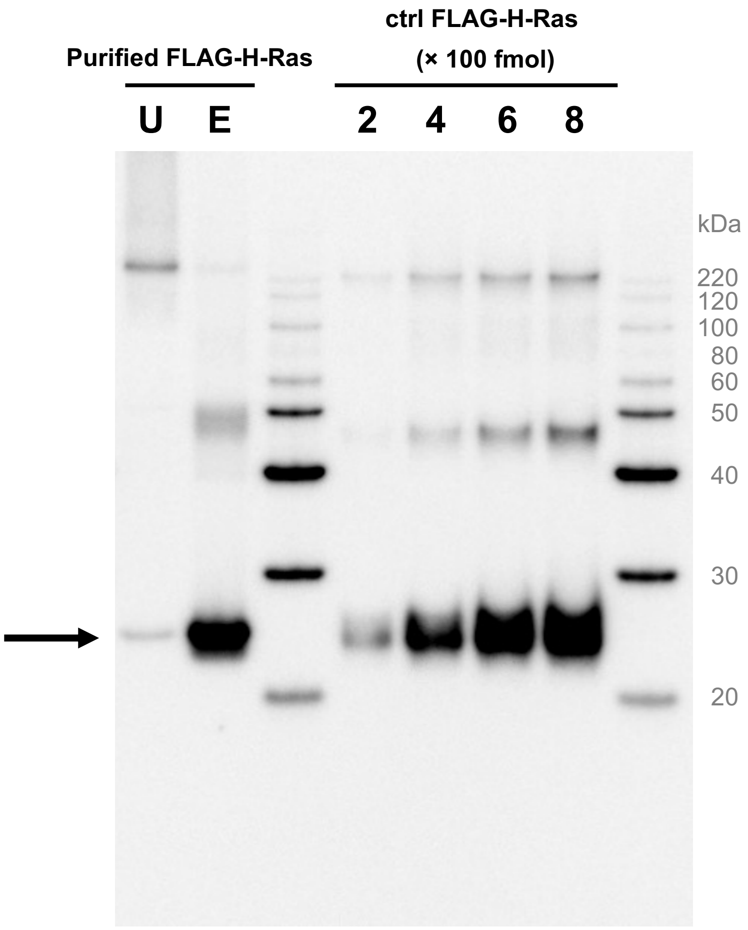

Figure S15. Western blotting of the FLAG-H-Ras isolated from the HeLa cells. The unbound fraction to the M2 antibody (U) and the elution fraction from the M2 antibody (E) were loaded onto the 15% SDS-PAGE gel, and then transferred onto a PVDF membrane. The membrane was probed with an anti-FLAG M2 antibody (Merck, 1:2,000 dilution). The secondary antibody was anti-mouse IgG antibody-HRP (Promega, 1:1,000 dilution). The FLAG-H-Ras protein band is indicated by the black arrow. *In vitro* FLAG-H-Ras protein samples (ctrl FLAG-H-Ras) were also loaded onto the same gel as a control.

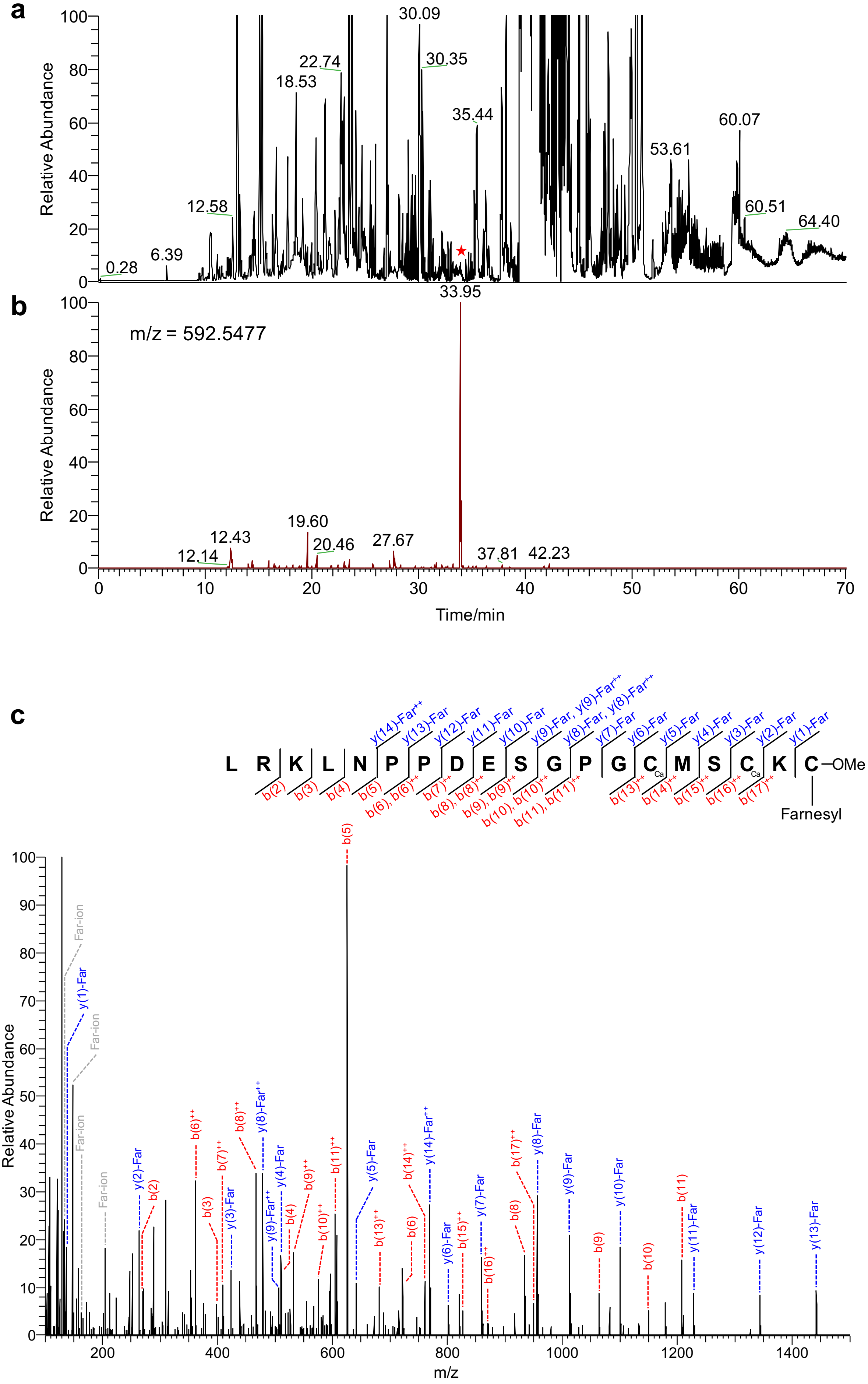
a
b
c
Relative Abundance
0.28
6.39
12.58
18.53
22.74
30.09
30.35
35.44
53.61
60.07
60.51
64.40
m/z = 592.5477
33.95
12.14
12.43
19.60
20.46
27.67
37.81
42.23
Time/min
L R K L N P P D E S G P G C M S C K C —OMe
Ca
Farnesyl
y(14)-Far++
y(13)-Far
y(12)-Far
y(11)-Far
y(10)-Far
y(9)-Far, y(9)-Far++
y(8)-Far, y(8)-Far++
y(7)-Far
y(6)-Far
y(5)-Far
y(4)-Far
y(3)-Far
y(2)-Far
y(1)-Far
b(2)
b(3)
b(4)
b(5)
b(6), b(6)++
b(7)++
b(8), b(8)++
b(9), b(9)++
b(10), b(10)++
b(11), b(11)++
b(13)++
b(14)++
b(15)++
b(16)++
b(17)++
Far-ion
m/z

Figure S16. LC-MS/MS analysis of the farnesylated C-terminal peptide from the isolated FLAG-H-Ras protein. (a) and (b) Base peak chromatograms from the analysis of the isolated FLAG-H-Ras protein. (a) Full MS, (b) Extracted ion chromatogram (m/z = 592.5477) for the peptide LRKLNPPDESGPGC(Ca)MSC(Ca)KC(Far)-OMe. The red asterisk indicates the retention time of the farnesylated C-terminal peptide (33.95 min). (c) MS/MS spectrum of the farnesylated C-terminal peptide, which corresponds to the peak at m/z = 592.5477. The observed fragment ions are indicated on the peptide sequence. The b- and y-ions are labeled in red and blue, respectively. The ions corresponding to the farnesyl group (marked in grey) were also detected. Ca, Far, and OMe are Carbamidomethyl, Farnesyl, and Carboxymethyl, respectively.

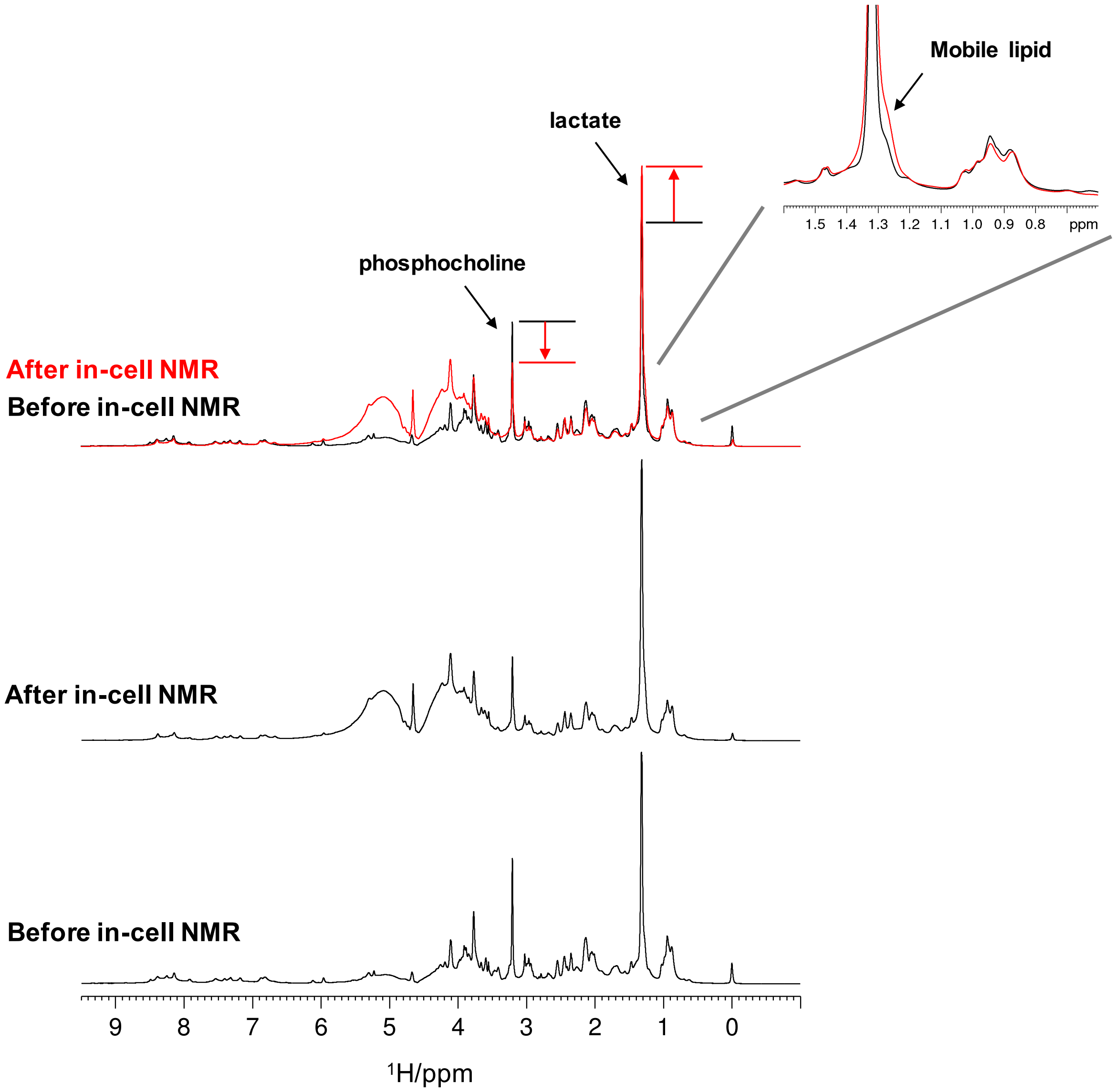

Figure S17. 1D ^{1}H-NMR spectra of the HeLa cells before and after in-cell NMR measurements. During 3 h in-cell NMR measurement, the peak intensity of lactate was increased and that of phosphocholine was attenuated. The intensity changes are indicated with red arrows in the superimposed spectra. The peak intensity of the mobile lipid was very slightly increased (shown in the expanded spectra).

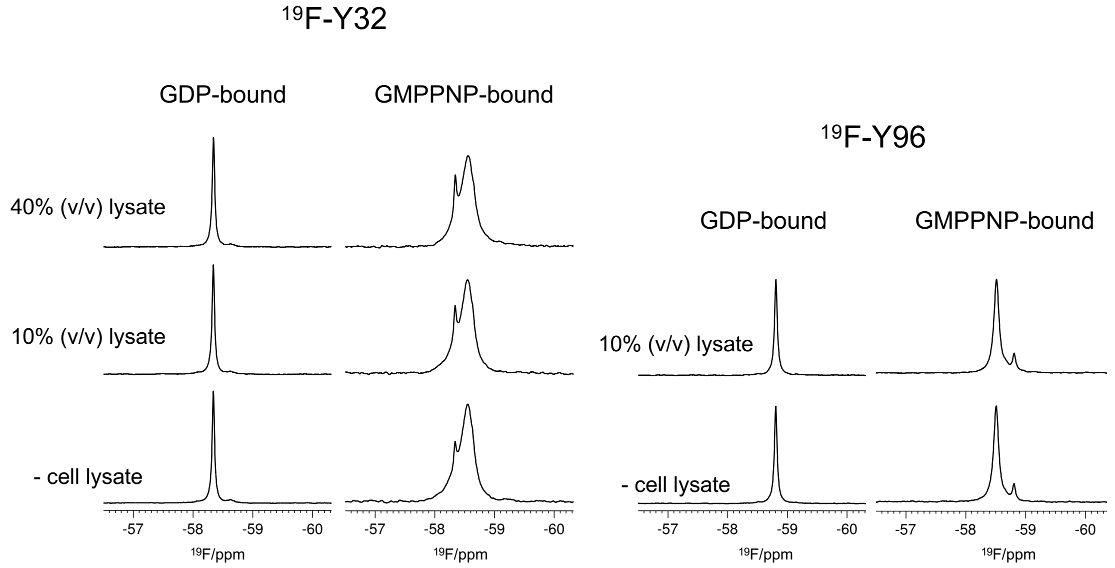

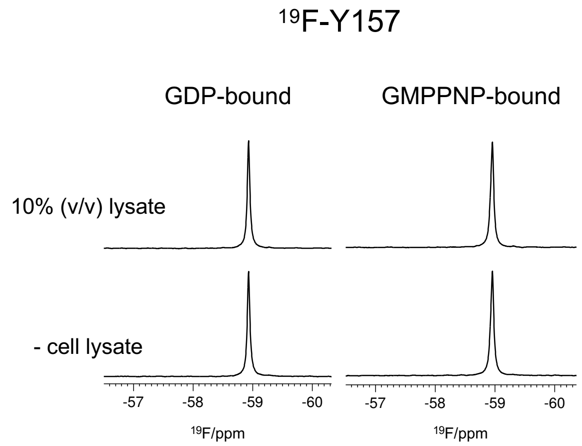

		linewidth/Hz	
	lysate/% (v/v)	GDP	GMPPNP
^{19}F-Y32	0	27.43	164.83
	10	27.61	164.77
	40	27.60	164.11
^{19}F-Y96	0	30.65	55.75
	10	30.46	55.79
^{19}F-Y157	0	27.76	32.77
	10	27.38	32.49

Figure S18. Effects of cell lysate on the NMR spectra of the ^{19}F-labeled H-Ras protein. *In vitro* 1D ^{19}F-NMR spectra of the ^{19}F-Y32, ^{19}F-Y96, and ^{19}F-Y157 H-Ras WT proteins in the absence and presence of the cell lysate are represented. The linewidths of each ^{19}F-NMR signal are summarized in the table. The ^{19}F-NMR signals of all ^{19}F-labeled H-Ras WT proteins were hardly affected by cell lysate in terms of their chemical shifts as well as linewidths.

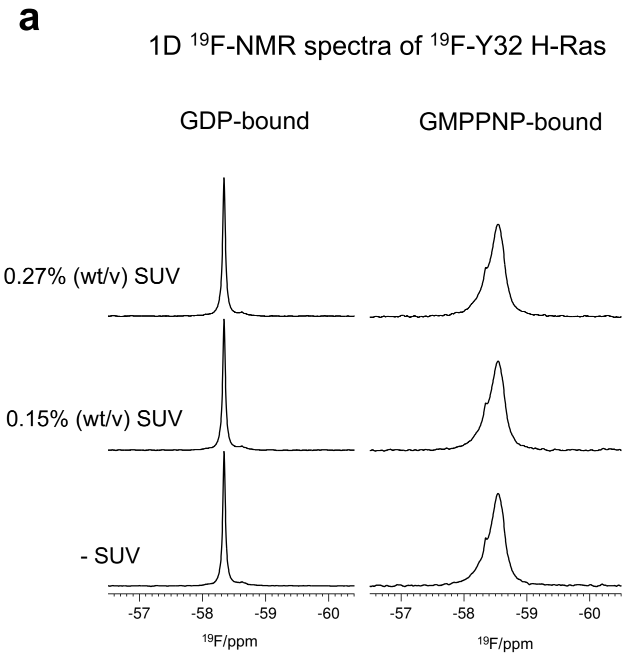

	linewidth/Hz	
SUV/% (wt/v)	GDP	GMPPNP
0	27.58	164.68
0.15	27.75	164.76
0.27	27.63	164.68

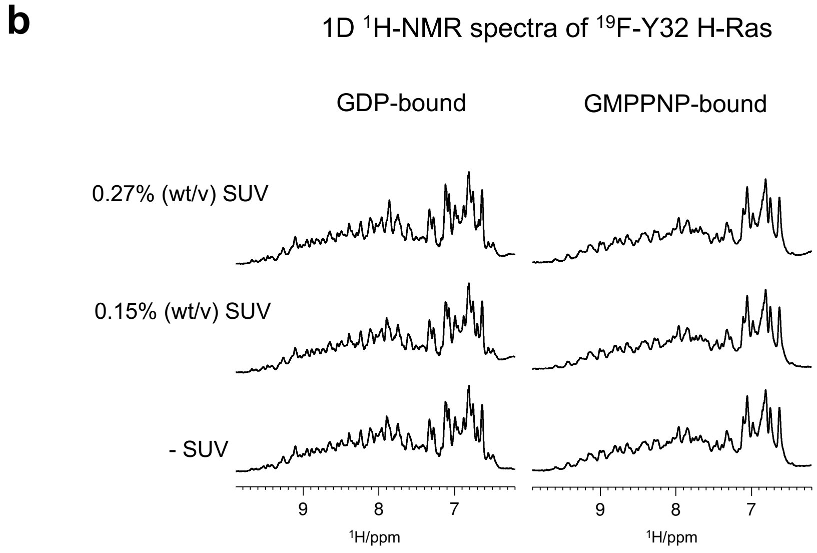

Figure S19. Effects of small unilamelar vesicle (SUV) liposome on the NMR spectra of the ^{19}F-labeled H-Ras protein. (a) *In vitro* 1D ^{19}F-NMR spectra of the ^{19}F-Y32 H-Ras WT protein in the absence and presence of SUVs. The linewidths of each ^{19}F-NMR signal are summarized in the table. The ^{19}F-NMR signals of ^{19}F-Y32 H-Ras WT proteins were hardly affected by SUVs in terms of their chemical shifts as well as linewidths. (b) Same as (a) except showing 1D ^{1}H-NMR spectra. Only aromatic and amide protons regions are represented. As with the case of the ^{19}F-NMR spectra, SUVs do not affect ^{1}H-NMR spectra of the ^{19}F-Y32 H-Ras WT protein.

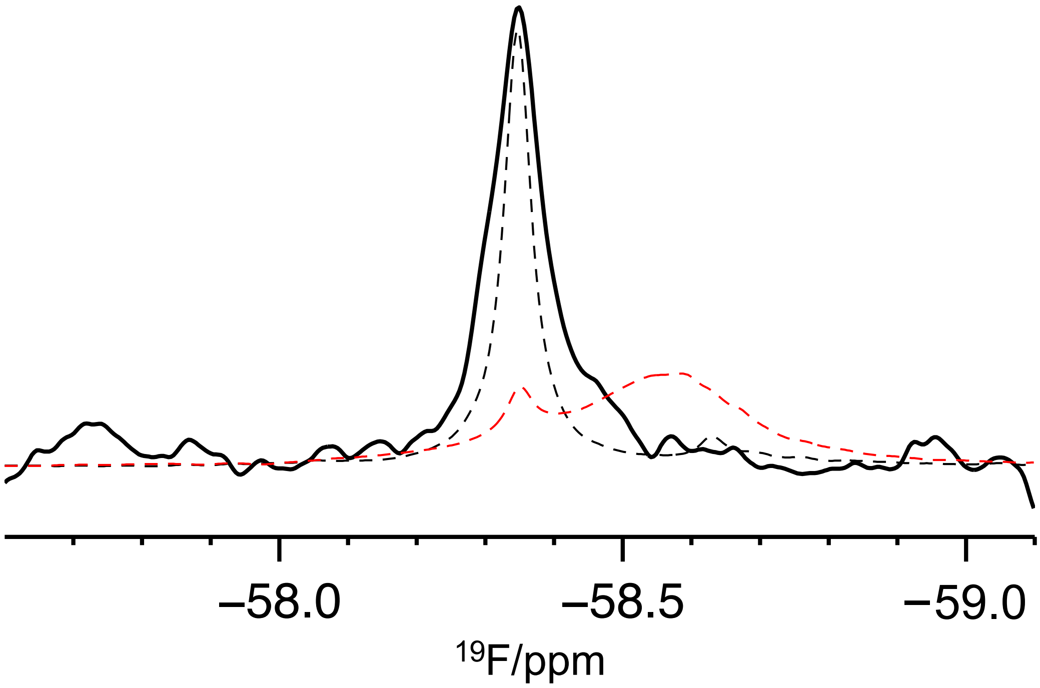

Figure S20. In-cell 1D ^{19}F-NMR spectrum of the GDP-bound ^{19}F-Y32 trH-Ras protein in HeLa cells. The black and red dashed spectra are the *in vitro* 1D ^{19}F-NMR spectra of the GDP- and GMPPNP-bound ^{19}F-Y32 H-Ras WT proteins, respectively, scaled down 4-fold relative to the in-cell NMR spectrum.

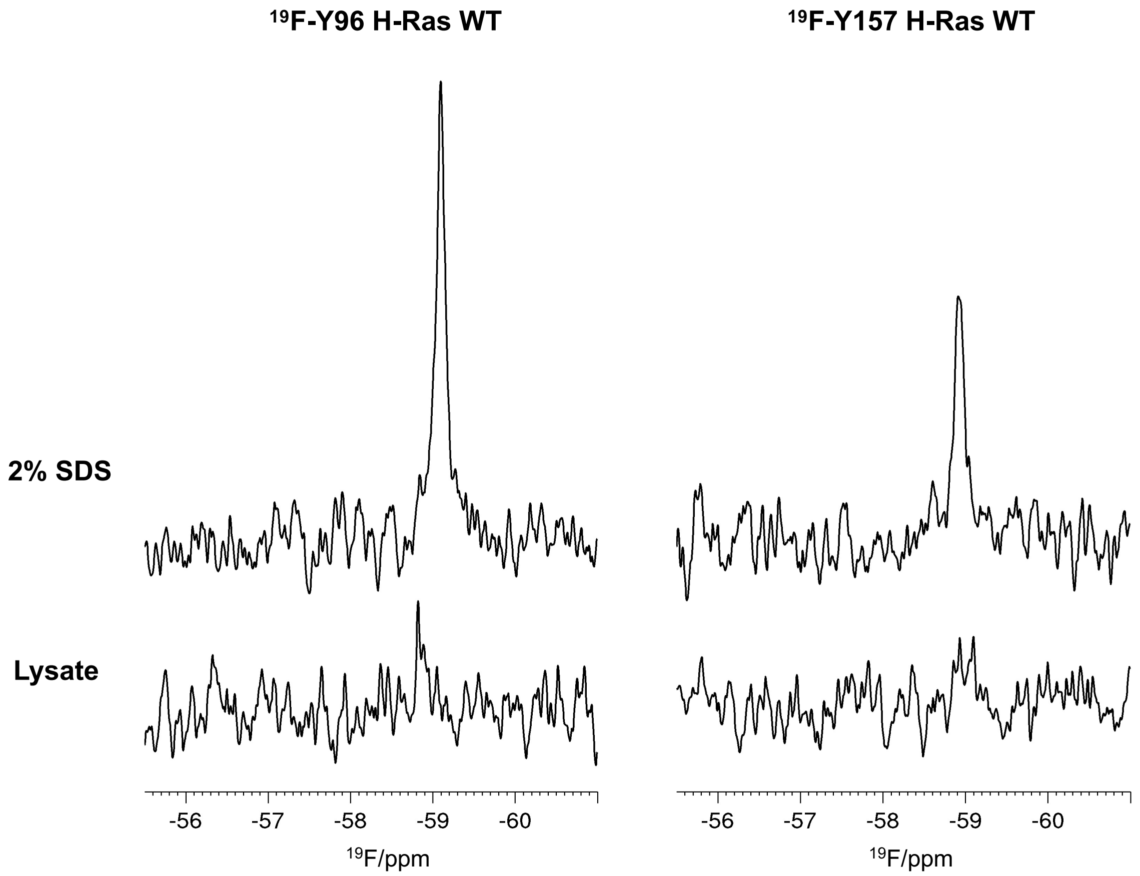

Figure S21. 1D ^{19}F-NMR spectra of the cell lysate and cellular fraction solubilized with 2% SDS. The GDP-bound ^{19}F-Y96 and ^{19}F-Y157 H-Ras WT proteins after their in-cell NMR measurements are exemplified.

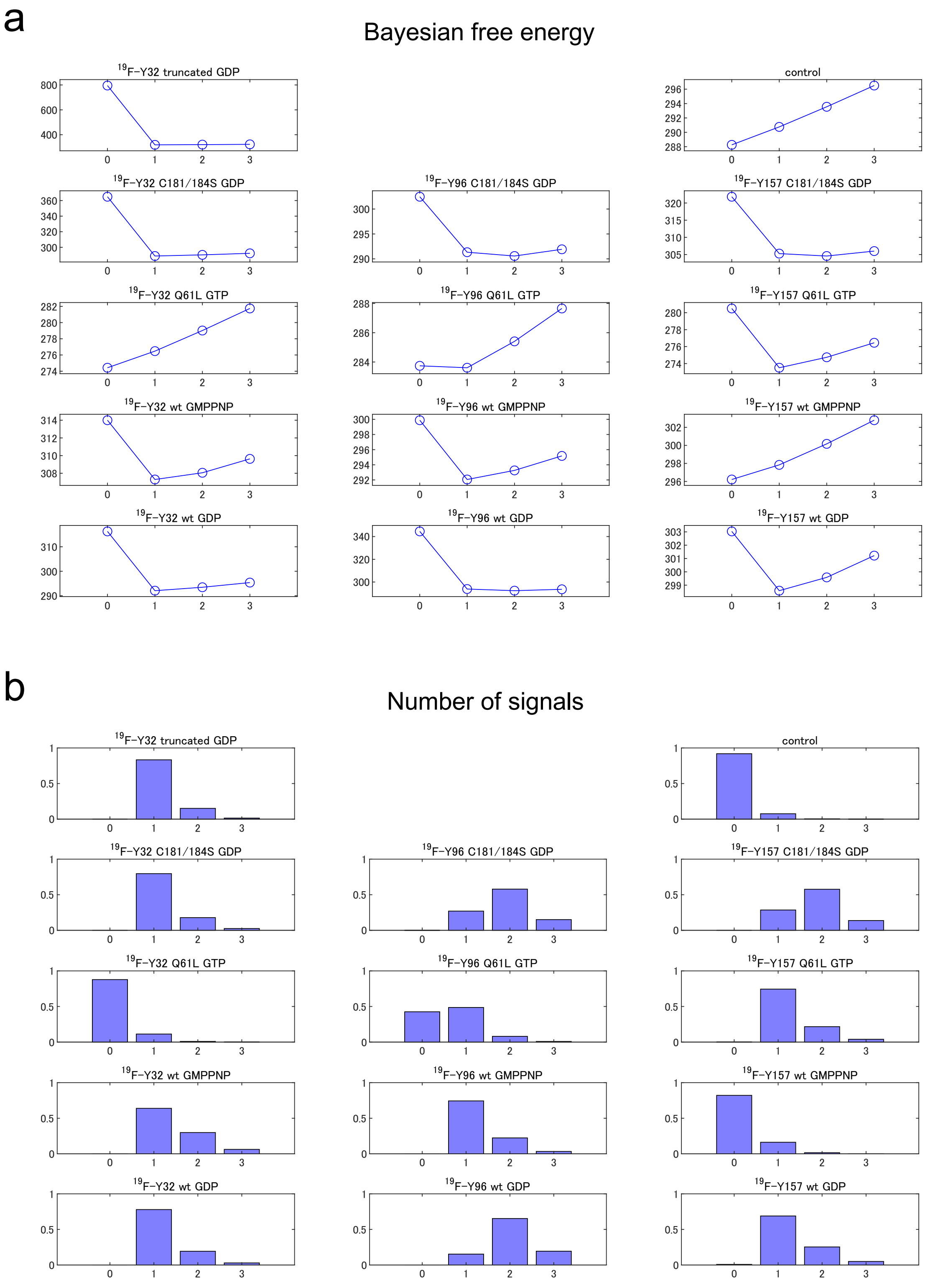

Figure S22. Verification of appropriate number of signal decomposition in the ^{19}F-NMR spectra. (a) Bayesian free energies of the spectral models assuming different number of Lorentz line-shaped signals, 0, 1, 2 and 3 for each in-cell NMR spectrum. (b) The probabilities of the number of signal component in each in-cell NMR spectrum.

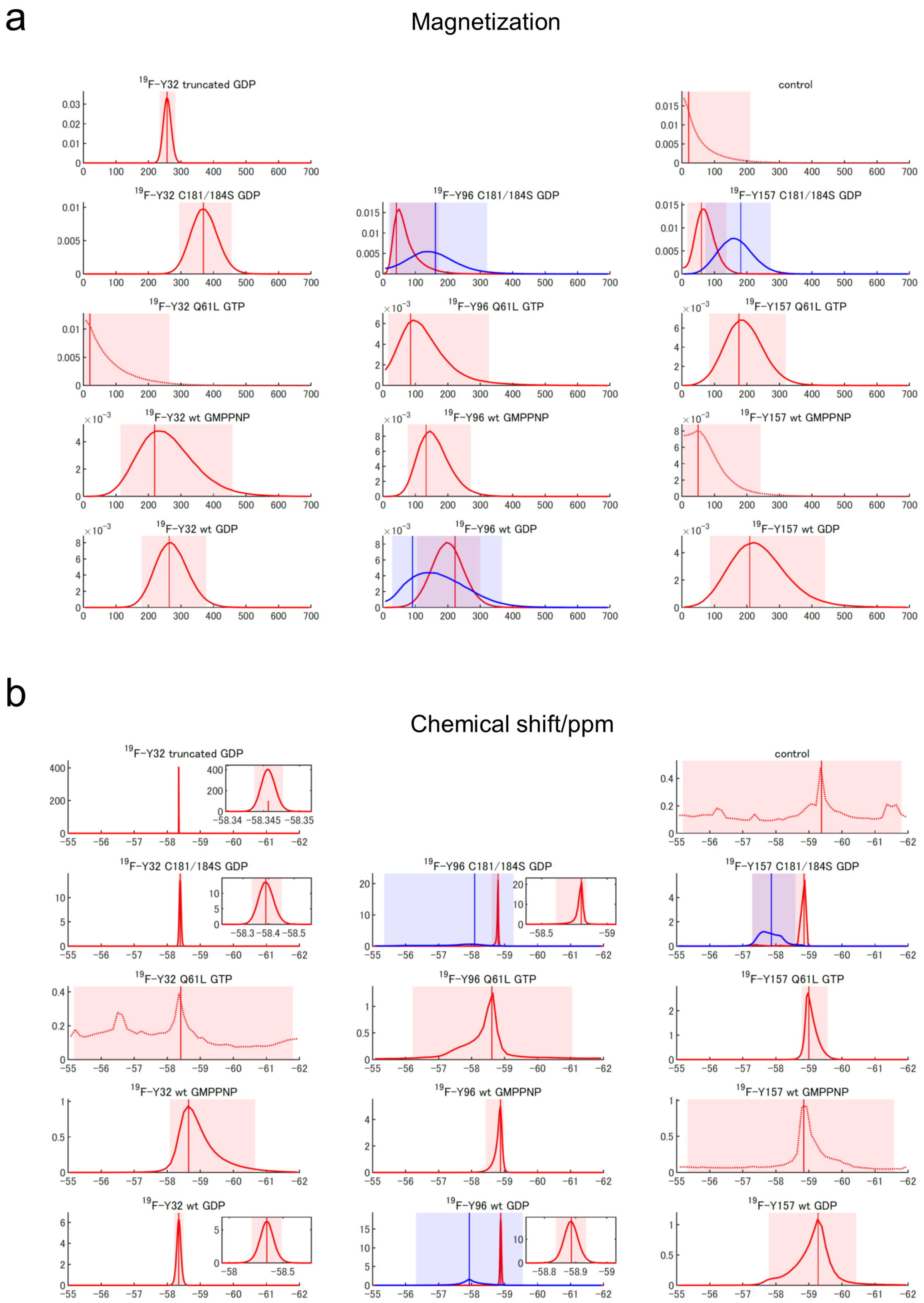
a
Magnetization
19F−Y32 truncated GDP
control
19F−Y32 C181/184S GDP
19F−Y96 C181/184S GDP
19F−Y157 C181/184S GDP
19F−Y32 Q61L GTP
19F−Y96 Q61L GTP
19F−Y157 Q61L GTP
19F−Y32 wt GMPPNP
19F−Y96 wt GMPPNP
19F−Y157 wt GMPPNP
19F−Y32 wt GDP
19F−Y96 wt GDP
19F−Y157 wt GDP
b
Chemical shift/ppm
19F−Y32 truncated GDP
control
19F−Y32 C181/184S GDP
19F−Y96 C181/184S GDP
19F−Y157 C181/184S GDP
19F−Y32 Q61L GTP
19F−Y96 Q61L GTP
19F−Y157 Q61L GTP
19F−Y32 wt GMPPNP
19F−Y96 wt GMPPNP
19F−Y157 wt GMPPNP
19F−Y32 wt GDP
19F−Y96 wt GDP
19F−Y157 wt GDP

C

Linewidth/Hz

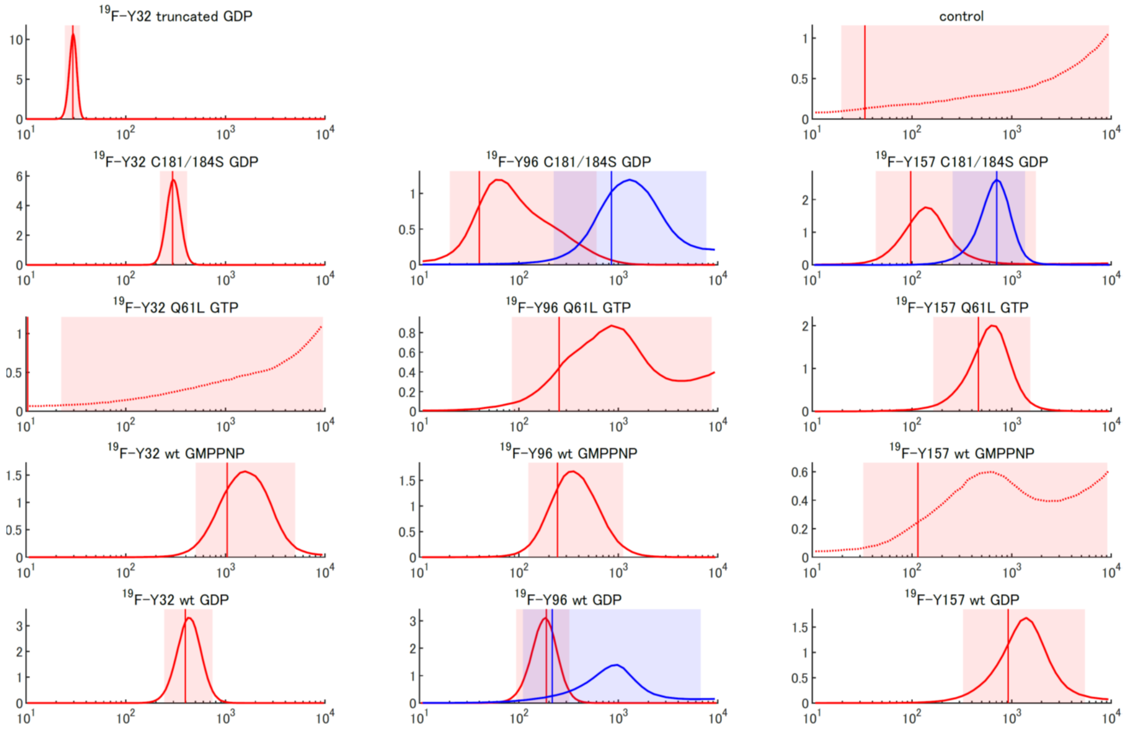

Figure S23. Bayesian spectral deconvolution of the in-cell NMR spectra. The posterior distributions of the magnetization (a), chemical shift (b), and signal linewidth (c) calculated by Bayesian inference using MCMC against the most probable number of signal component (either 1 or 2) obtained from the Bayesian free energies (see Figure S22). In the case that 0- or 1-signal model is the most probable, the posterior probabilities were calculated as 1-signal model and shown in red dashed- (0-signal model) or solid- (1-signal model) curves. In the case that 2-signal model is the most probable, the posterior probabilities were calculated as 2-signal model and shown in red and blue curves. The vertical lines indicate MAP estimators. The red and blue boxes show 95% credible intervals.

^{19}F-Y32 WT GDP, assuming 1 signal

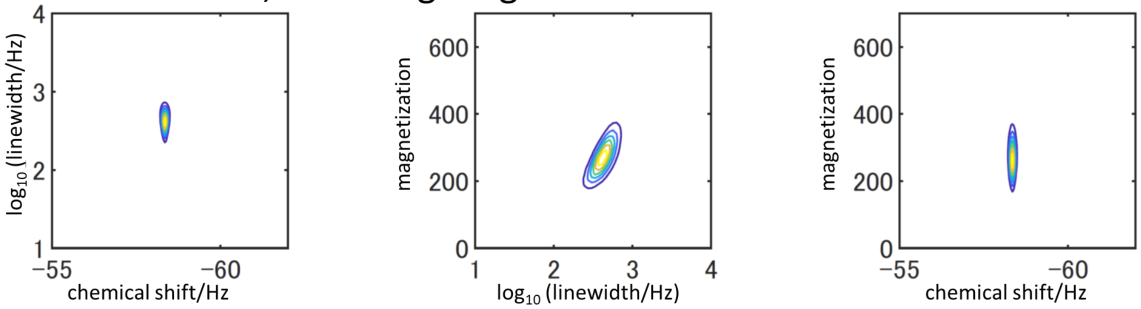

^{19}F-Y32 WT GMPPNP, assuming 1 signal

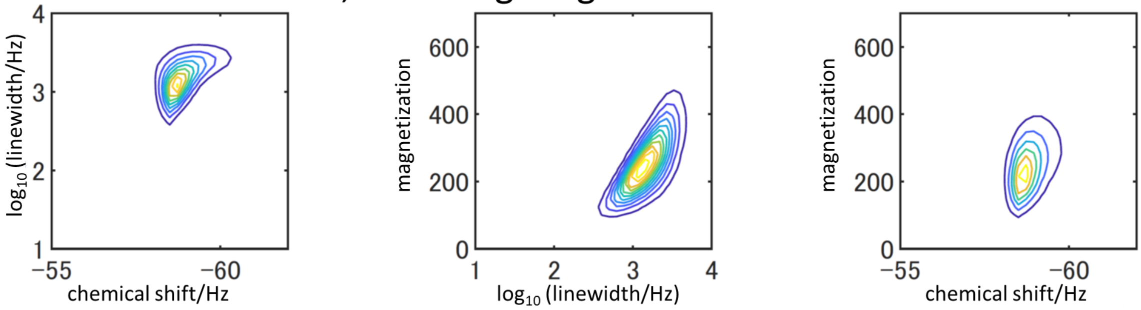

^{19}F-Y32 Q61L GTP, assuming 1 signal

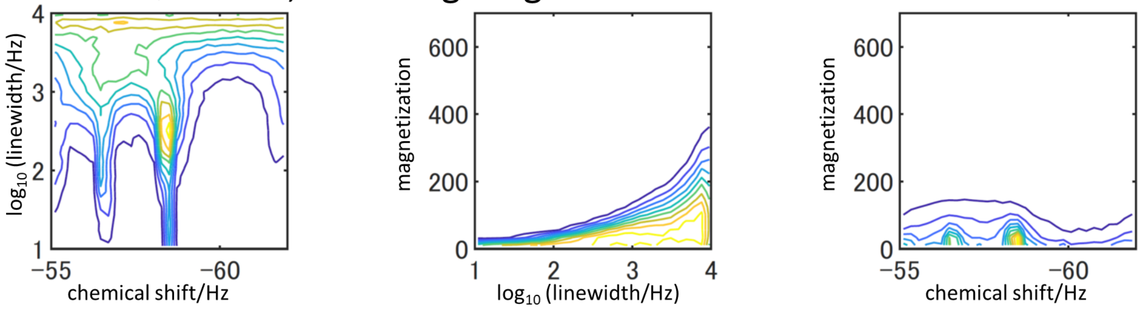

^{19}F-Y32 C181/184S GDP, assuming 1 signal

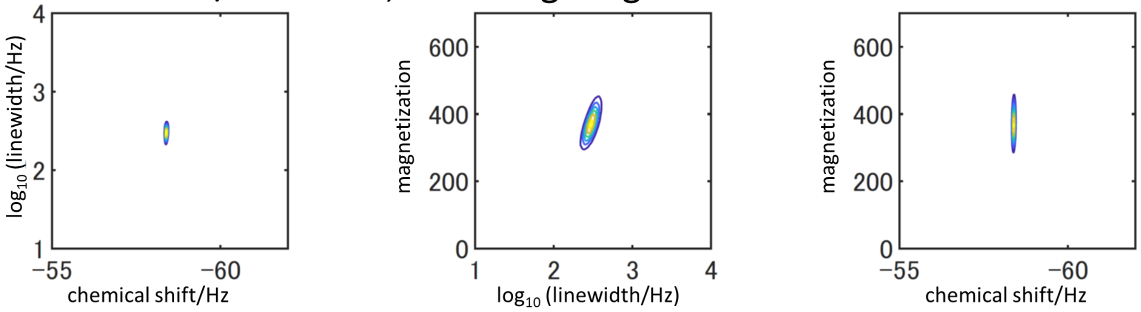

^{19}F-Y32 truncated GDP, assuming 1 signal

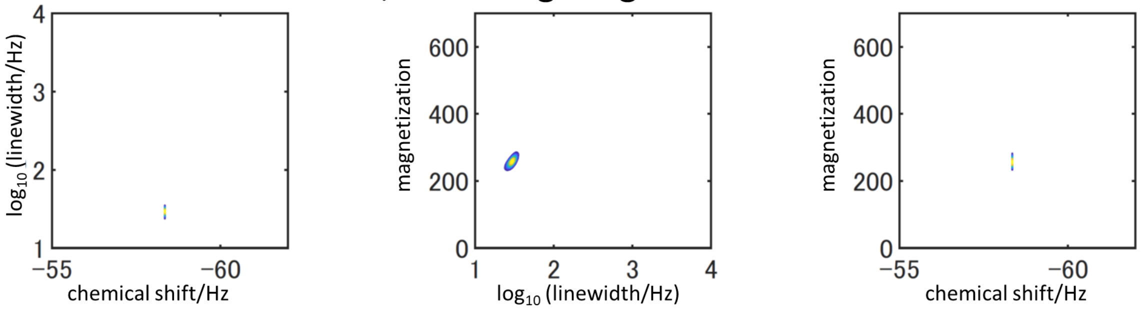

^{19}F-Y96 WT GDP, assuming 2 signals, signal #1

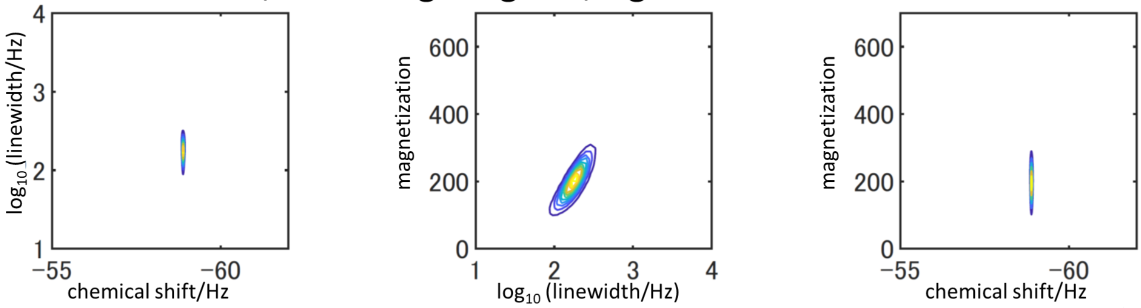

^{19}F-Y96 WT GDP, assuming 2 signals, signal #2

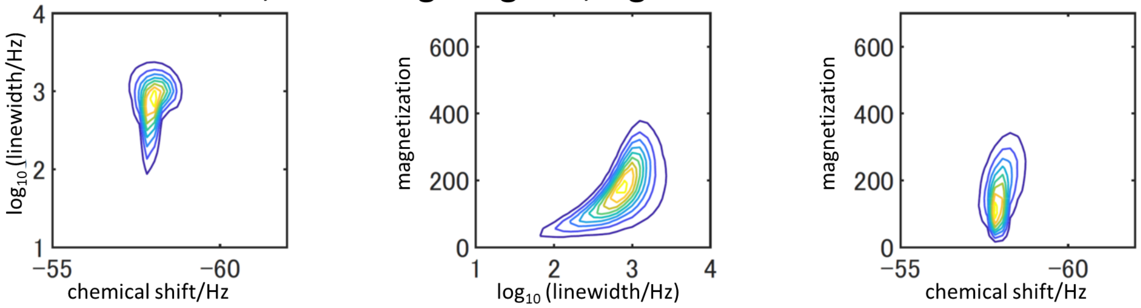

^{19}F-Y96 WT GMPPNP, assuming 1 signal

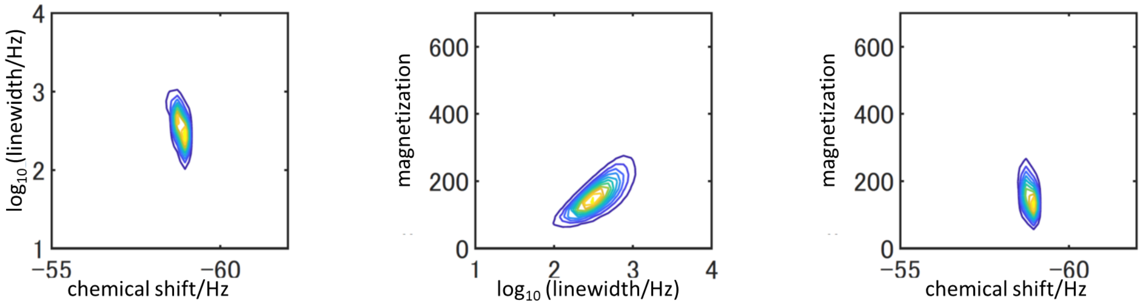

^{19}F-Y96 Q61L GTP, assuming 1 signal

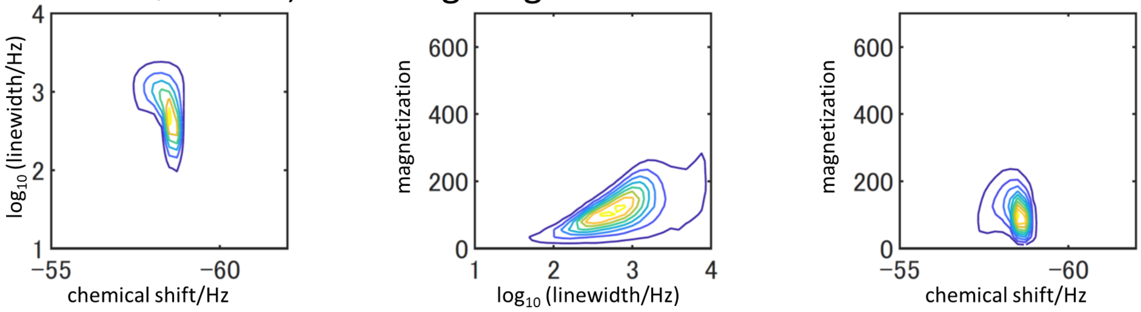

^{19}F-Y96 C181/184S GDP, assuming 2 signals, signal #1

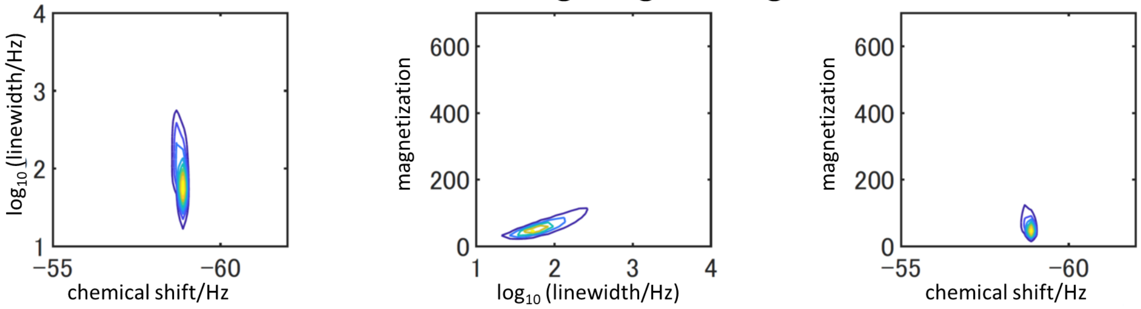

^{19}F-Y96 C181/184S GDP, assuming 2 signals, signal #2

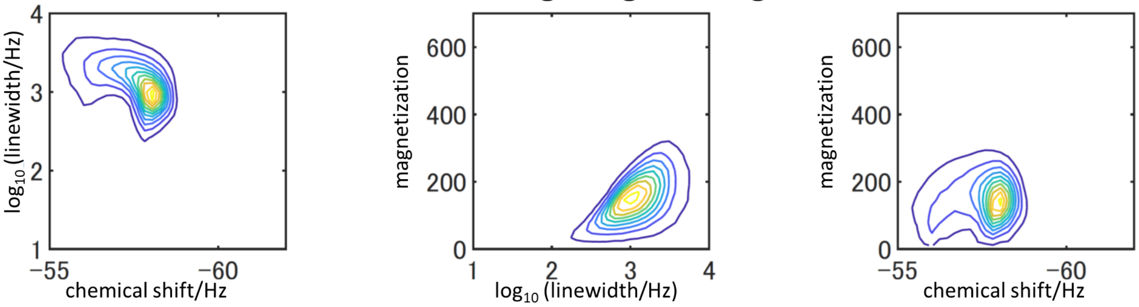

^{19}F-Y157 WT GDP, assuming 1 signal

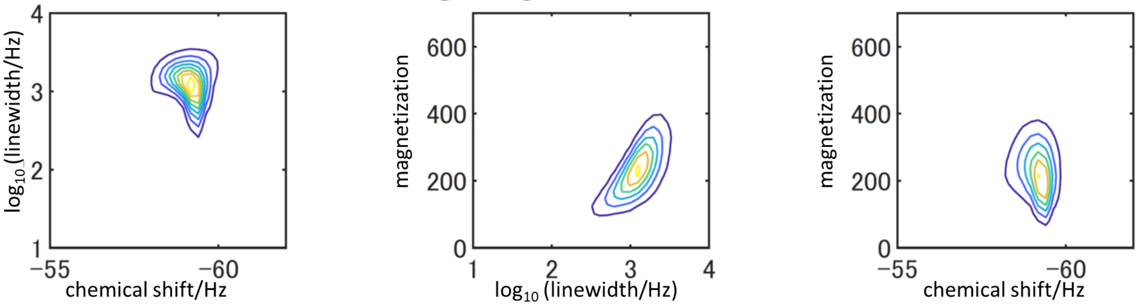

^{19}F-Y157 WT GMPPNP, assuming 1 signal

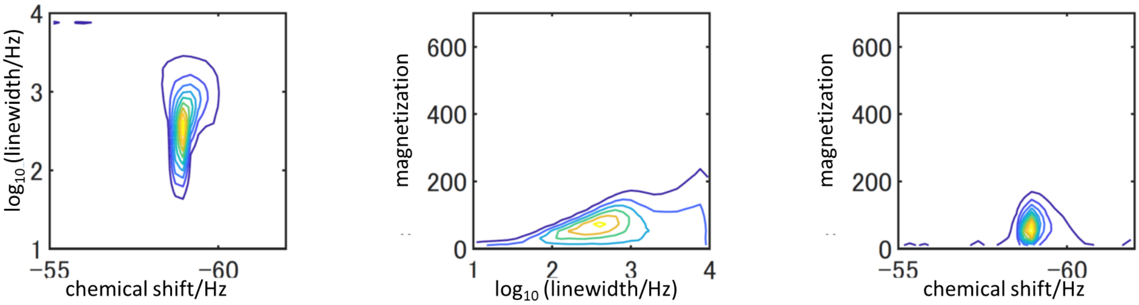

^{19}F-Y157 Q61L GTP, assuming 1 signal

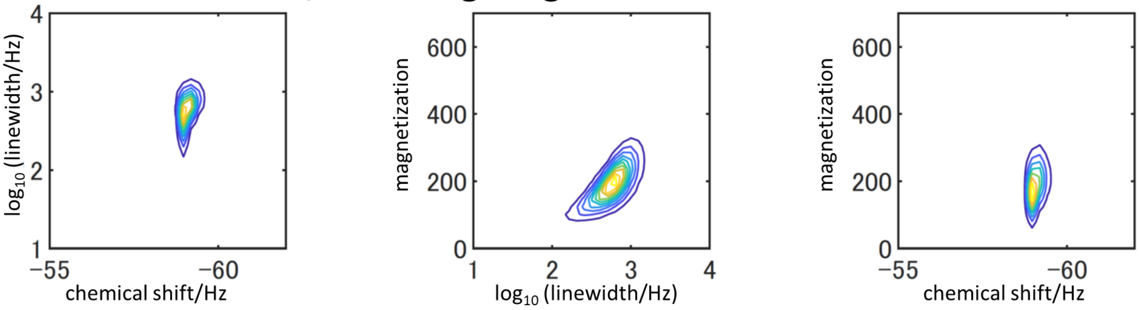

^{19}F-Y157 C181/184S GDP, assuming 2 signals, signal #1

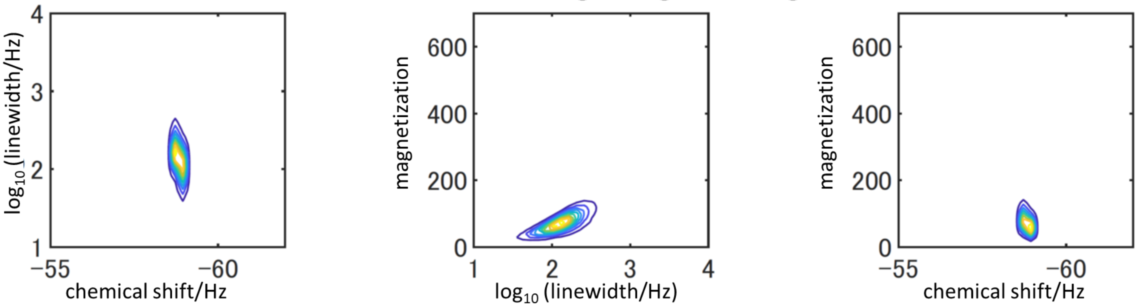

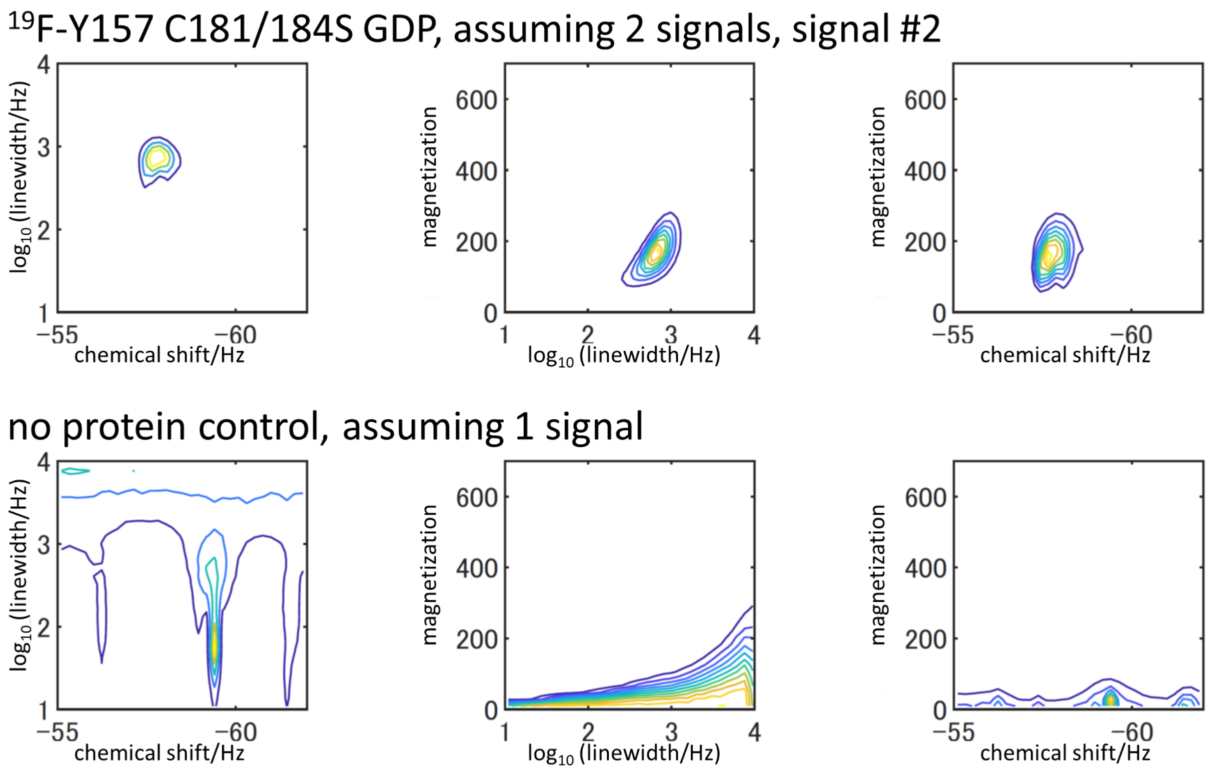

Figure S24. Two-dimensional joint posterior distribution of the pairs of the estimated parameters in Bayesian spectral deconvolution.

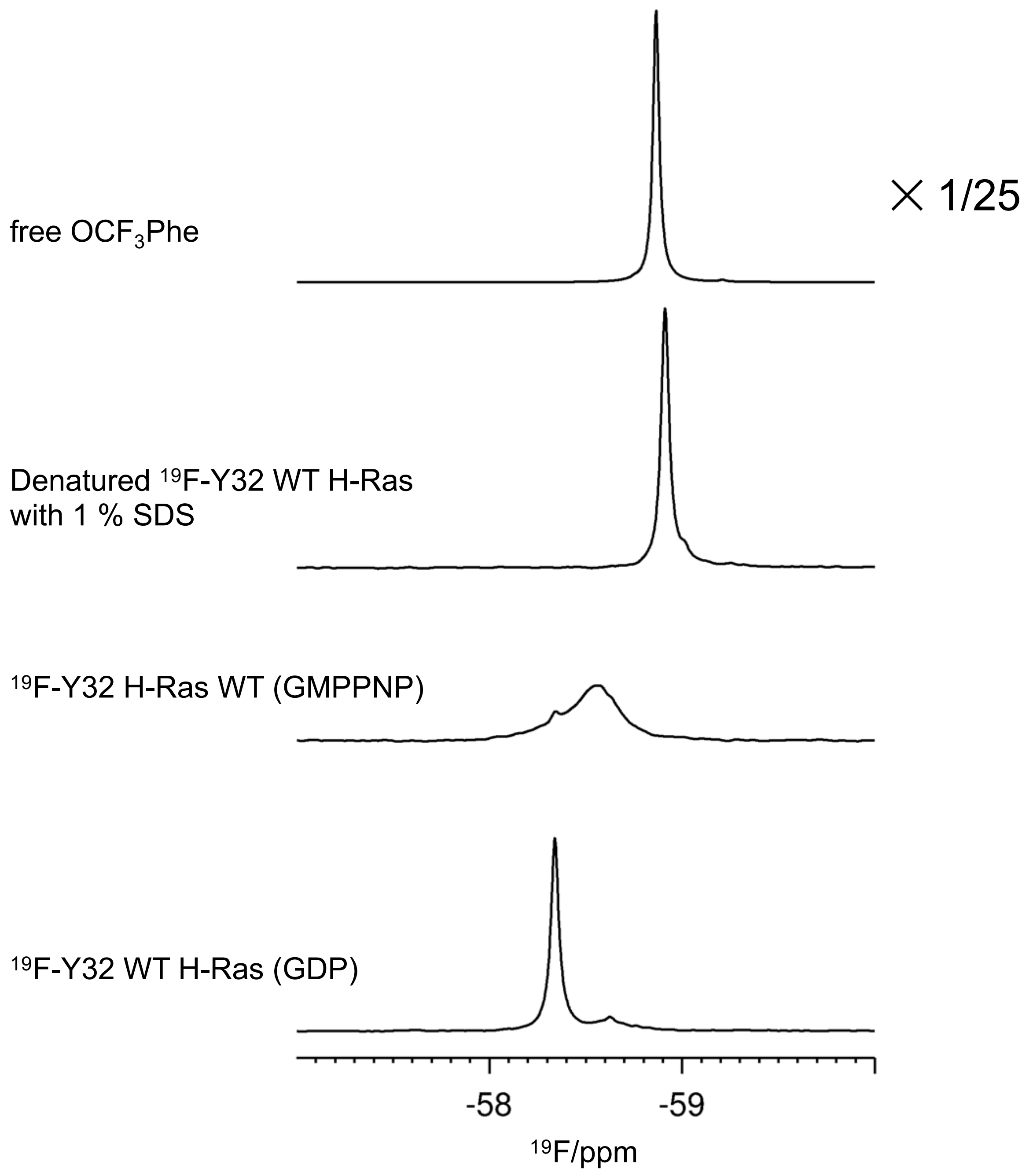

Figure S25. Comparison of *In vitro* 1D ^{19}F-NMR spectra. The spectra of GDP-bound ^{19}F-Y32 H-Ras WT, GMPPNP-bound ^{19}F-Y32 H-Ras WT, denatured ^{19}F-Y32 H-Ras WT with 1 % SDS, and free OCF_3Phe are shown from the bottom to top.

3. Supporting Tables

Table S1. The amino acid sequences of the proteins prepared in this study.

Name	Sequence
H-Ras WT	GSSGSSGMTEYKLVVVGAGGVGKSALTIQLIQNHFVDEYDPTIEDSYRKQVVIDGETCLLDILDTAGQEEYSAMRDQYMRTGEGFLCVFAINNTKSFEDIHQYREQIKRVKDSDDVPMVLVGNKCDLAARTVESRQAQDLARSYGIPYIETSAKTRQGVEDAFYTLVREIRQHKLRKLNPPDESGPGCMSCKCVLS
^{19}F-Y32 H-Ras WT[1]	GSSGSSGMTEYKLVVVGAGGVGKSALTIQLIQNHFVDEXDPTIEDSYRKQVVIDGETCLLDILDTAGQEEYSAMRDQYMRTGEGFLCVFAINNTKSFEDIHQYREQIKRVKDSDDVPMVLVGNKCDLAARTVESRQAQDLARSYGIPYIETSAKTRQGVEDAFYTLVREIRQHKLRKLNPPDESGPGCMSCKCVLS
^{19}F-Y96 H-Ras WT[1]	GSSGSSGMTEYKLVVVGAGGVGKSALTIQLIQNHFVDEYDPTIEDSYRKQVVIDGETCLLDILDTAGQEEYSAMRDQYMRTGEGFLCVFAINNTKSFEDIHQXREQIKRVKDSDDVPMVLVGNKCDLAARTVESRQAQDLARSYGIPYIETSAKTRQGVEDAFYTLVREIRQHKLRKLNPPDESGPGCMSCKCVLS
^{19}F-Y157 H-Ras WT[1]	GSSGSSGMTEYKLVVVGAGGVGKSALTIQLIQNHFVDEYDPTIEDSYRKQVVIDGETCLLDILDTAGQEEYSAMRDQYMRTGEGFLCVFAINNTKSFEDIHQYREQIKRVKDSDDVPMVLVGNKCDLAARTVESRQAQDLARSYGIPYIETSAKTRQGVEDAFXTLVREIRQHKLRKLNPPDESGPGCMSCKCVLS
H-Ras Q61L	GSSGSSGMTEYKLVVVGAGGVGKSALTIQLIQNHFVDEYDPTIEDSYRKQVVIDGETCLLDILDTAGLEEYSAMRDQYMRTGEGFLCVFAINNTKSFEDIHQYREQIKRVKDSDDVPMVLVGNKCDLAARTVESRQAQDLARSYGIPYIETSAKTRQGVEDAFYTLVREIRQHKLRKLNPPDESGPGCMSCKCVLS
^{19}F-Y32 H-Ras Q61L[1]	GSSGSSGMTEYKLVVVGAGGVGKSALTIQLIQNHFVDEXDPTIEDSYRKQVVIDGETCLLDILDTAGLEEYSAMRDQYMRTGEGFLCVFAINNTKSFEDIHQYREQIKRVKDSDDVPMVLVGNKCDLAARTVESRQAQDLARSYGIPYIETSAKTRQGVEDAFYTLVREIRQHKLRKLNPPDESGPGCMSCKCVLS
^{19}F-Y96 H-Ras Q61L[1]	GSSGSSGMTEYKLVVVGAGGVGKSALTIQLIQNHFVDEYDPTIEDSYRKQVVIDGETCLLDILDTAGLEEYSAMRDQYMRTGEGFLCVFAINNTKSFEDIHQXREQIKRVKDSDDVPMVLVGNKCDLAARTVESRQAQDLARSYGIPYIETSAKTRQGVEDAFYTLVREIRQHKLRKLNPPDESGPGCMSCKCVLS
^{19}F-Y157 H-Ras Q61L[1]	GSSGSSGMTEYKLVVVGAGGVGKSALTIQLIQNHFVDEYDPTIEDSYRKQVVIDGETCLLDILDTAGLEEYSAMRDQYMRTGEGFLCVFAINNTKSFEDIHQYREQIKRVKDSDDVPMVLVGNKCDLAARTVESRQAQDLARSYGIPYIETSAKTRQGVEDAFXTLVREIRQHKLRKLNPPDESGPGCMSCKCVLS
H-Ras C181S/184S	GSSGSSGMTEYKLVVVGAGGVGKSALTIQLIQNHFVDEYDPTIEDSYRKQVVIDGETCLLDILDTAGQEEYSAMRDQYMRTGEGFLCVFAINNTKSFEDIHQYREQIKRVKDSDDVPMVLVGNKCDLAARTVESRQAQDLARSYGIPYIETSAKTRQGVEDAFYTLVREIRQHKLRKLNPPDESGPGSMSSKCVLS
^{19}F-Y32 H-Ras C181S/184S[1]	GSSGSSGMTEYKLVVVGAGGVGKSALTIQLIQNHFVDEXDPTIEDSYRKQVVIDGETCLLDILDTAGQEEYSAMRDQYMRTGEGFLCVFAINNTKSFEDIHQYREQIKRVKDSDDVPMVLVGNKCDLAARTVESRQAQDLARSYGIPYIETSAKTRQGVEDAFYTLVREIRQHKLRKLNPPDESGPGSMSSKCVLS
^{19}F-Y96 H-Ras C181S/184S[1]	GSSGSSGMTEYKLVVVGAGGVGKSALTIQLIQNHFVDEYDPTIEDSYRKQVVIDGETCLLDILDTAGQEEYSAMRDQYMRTGEGFLCVFAINNTKSFEDIHQXREQIKRVKDSDDVPMVLVGNKCDLAARTVESRQAQDLARSYGIPYIETSAKTRQGVEDAFYTLVREIRQHKLRKLNPPDESGPGSMSSKCVLS
^{19}F-Y157 H-Ras C181S/184S[1]	GSSGSSGMTEYKLVVVGAGGVGKSALTIQLIQNHFVDEYDPTIEDSYRKQVVIDGETCLLDILDTAGQEEYSAMRDQYMRTGEGFLCVFAINNTKSFEDIHQYREQIKRVKDSDDVPMVLVGNKCDLAARTVESRQAQDLARSYGIPYIETSAKTRQGVEDAFXTLVREIRQHKLRKLNPPDESGPGSMSSKCVLS
trH-Ras	GSSGSSGMTEYKLVVVGAGGVGKSALTIQLIQNHFVDEYDPTIEDSYRKQVVIDGETCLLDILDTAGQEEYSAMRDQYMRTGEGFLCVFAINNTKSFEDIHQYREQIKRVKDSDDVPMVLVGNKCDLAARTVESRQAQDLARSYGIPYIETSAKTRQGVEDAFYTLVREIRQHKLRKL
^{19}F-Y32 trH-Ras[1]	GSSGSSGMTEYKLVVVGAGGVGKSALTIQLIQNHFVDEXDPTIEDSYRKQVVIDGETCLLDILDTAGQEEYSAMRDQYMRTGEGFLCVFAINNTKSFEDIHQYREQIK

	RVKDSDDVPMVLVGNKCDLAARTVESRQAQDLARSYGIPYIETSAKTRQGVEDA FYTLVREIRQHKLRKL
FLAG-H-Ras	GDYKDDDDKSSGSSGMTEYKLVVVGAGGVGKSALTIQLIQNHFVDEYDPTIEDS YRKQVVIDGETCLLDILDTAGQEEYSAMRDQYMRTGEGFLCVFAINNTKSFEDI HQYREQIKRVKDSDDVPMVLVGNKCDLAARTVESRQAQDLARSYGIPYIETSAK TRQGVEDAFYTLVREIRQHKLRKLNPPDESGPGCMSCKCVLS
Raf1 RBD	GPLGSMPSKTSNTIRVFLPNKQRTVVNVRNGMSLHDCLMKALKVRGLQPECCAV FRLLHEHKGKKARLDWNTDAASLIGEELQVDFLD
RGL RBD	GPLGSMSITSTVLPPVYNQQNEDTCIIRISVEDNNGNMYKSIMLTSQDKTPAVI QRAMLKHNLDSDPAEEYELVQVISEDKELVIPDSANVFYAMNSQVNFDFILRKK NILERPHRD
GST-Raf1 RBD	MSPILGYWKIKGLVQPTRLLLEYLEEKYEEHLYERDEGDKWRNKKFELGLEFPN LPYYIDGDVKLTQSMAIIRYIADKHNMLGGCPKERAEISMLEGAVLDIRYGVSR IAYSKDFETLKVDFLSKLPEMLKMFEDRLCHKTYLNGDHVTHPDFMLYDALDVV LYMDPMCLDAFPKLVCFKKRIEAIPQIDKYLKSSKYIAWPLQGWQATFGGGDHP PKSDLEVLFQGPLGSMPSKTSNTIRVFLPNKQRTVVNVRNGMSLHDCLMKALKV RGLQPECCAVFRLLHEHKGKKARLDWNTDAASLIGEELQVDFLD
GST-RGL RBD	MSPILGYWKIKGLVQPTRLLLEYLEEKYEEHLYERDEGDKWRNKKFELGLEFPN LPYYIDGDVKLTQSMAIIRYIADKHNMLGGCPKERAEISMLEGAVLDIRYGVSR IAYSKDFETLKVDFLSKLPEMLKMFEDRLCHKTYLNGDHVTHPDFMLYDALDVV LYMDPMCLDAFPKLVCFKKRIEAIPQIDKYLKSSKYIAWPLQGWQATFGGGDHP PKSDLEVLFQGPLGSMSITSTVLPPVYNQQNEDTCIIRISVEDNNGNMYKSIML TSQDKTPAVIQRAMLKHNLDSDPAEEYELVQVISEDKELVIPDSANVFYAMNSQ VNFDFILRKKNILERPHRD
OCF3Phe-RS	MDEFEMIKRNTSEIISEEELREVLKKDEKSAYIGFEPSGKIHLGHYLQIKKMID LQNAGFDIIILLADLHAYLNQKGELDEIRKIGDYNKKVFEAMGLKAKYVYGSEF QLDKDYTLNVYRLALKTTLKRARRSMELIAREDENPKVAEVIYPIMQVNDIHYL GVDVAVGGMEQRKIHMLARELLPKKVVCIHNPVLTGLDGEGKMSSSKGNFIAVD DSPEEIRAKIKKAYCPAGVVEGNPIMEIAKYFLEYPLTIKRPEKFGGDLTVNSY EELESLFKNKELHPMDLKNAVAEELIKILEPIRKRLENLYFQ

[1]"X" denotes OCF3Phe.

Table S2. MAP estimators and 95% credible intervals (indicated in brackets) of Bayesian spectral deconvolution of the in-cell NMR spectra.

	Magnetization	Chemical shift / ppm	Line width[a] / Hz
^{19}F-Y32 WT GDP	263 (179–377)	−58.35 (−58.49 to −58.21)	396 (243–740)
^{19}F-Y32 WT GMPPNP	218 (114–458)	−58.6 (−60.7 to −58.1)	1041 (502–4997)
^{19}F-Y32 Q61L GTP[b]	19 (2–263)	−58.4 (−61.8 to −55.2)	10 (22–9505)
^{19}F-Y32 C181/184S GDP	368 (295–455)	−58.39 (−58.45 to −58.33)	293 (220–411)
^{19}F-Y32 truncated GDP	257 (234–281)	−58.346 (−58.348 to −58.344)	29 (24–34)
^{19}F-Y96 WT GDP	222 (105–299)	−58.89 (−58.93 to −58.84)	189 (93–320)
	91 (28–366)	-57.93 (−59.55 to −56.31)	215 (109–6717)
^{19}F-Y96 WT GMPPNP	133 (76–270)	−58.87 (−58.98 to −58.43)	244 (124–1114)
^{19}F-Y96 Q61L GTP	84 (16–326)	−58.6 (−61.0 to −56.2)	252 (84–8651)
^{19}F-Y96 C181/184S GDP	41 (23–171)	−58.80 (−58.84 to −58.61)	39 (20–600)
	161 (18–320)	−58.09 (−59.27 to −55.35)	851 (223–7654)
^{19}F-Y157 WT GDP	208 (86–440)	−59.3 (−60.4 to −57.8)	924 (325–5438)
^{19}F-Y157 WT GMPPNP[b]	49 (3–241)	−58.8 (−61.6 to −55.3)	114 (32–9104)
^{19}F-Y157 Q61L GTP	175 (84–318)	−59.00 (−59.56 to −58.78)	463 (163–1535)
^{19}F-Y157 C181/184S GDP	59 (16–136)	−58.85 (−58.92 to −57.28)	96 (43–1750)
	181 (72–273)	−57.87 (−58.59 to −57.29)	709 (256–1360)
control[b]	20 (1–209)	−59.4 (−61.8 to −55.2)	33 (19–9482)

[a]Line widths are defined as FWHM.

[b]The most probable model by the Bayesian free energy analysis is the 0-signal model (Figure S22). However, the parameters shown are obtained from the 1-signal model instead of the 0-signal model.

References

(1) Cellitti, S. E.; Jones, D. H.; Lagpacan, L.; Hao, X.; Zhang, Q.; Hu, H.; Brittain, S. M.; Brinker, A.; Caldwell, J.; Bursulaya, B.; et al. *In vivo* incorporation of unnatural amino acids to probe structure, dynamics, and ligand binding in a large protein by nuclear magnetic resonance spectroscopy. *J. Am. Chem. Soc.* **2008**, *130*, 9268-9281.
(2) Kigawa, T.; Muto, Y.; Yokoyama, S. Cell-free synthesis and amino acid-selective stable isotope labeling of proteins for NMR analysis. *J. Biomol. NMR* **1995**, *6*, 129–134.
(3) Kigawa, T.; Yabuki, T.; Matsuda, N.; Matsuda, T.; Nakajima, R.; Tanaka, A.; Yokoyama, S. Preparation of *Escherichia coli* cell extract for highly productive cell-free protein expression. *J. Struct. Funct. Genomics* **2004**, *5*, 63–68.
(4) Matsuda, T.; Kigawa, T.; Koshiba, S.; Inoue, M.; Aoki, M.; Yamasaki, K.; Seki, M.; Shinozaki, K.; Yokoyama, S. Cell-free synthesis of zinc-binding proteins. *J. Struct. Funct. Genomics* **2006**, *7*, 93–100.
(5) Kigawa, T.; Yabuki, T.; Yoshida, M.; Tsutsui, M.; Ito, Y.; Shibata, T.; Yokoyama, S. Cell-free production and stable-isotope labeling of milligram quantities of proteins. *FEBS Lett.* **1999**, *442*, 15–19.
(6) Wang, L.; Magliery, T. J.; Liu, D. R.; Schultz, P. G. A new functional suppressor tRNA/aminoacyl−tRNA synthetase pair for the in vivo incorporation of unnatural amino acids into proteins. *J. Am. Chem. Soc.* **2000**, *122*, 5010-5011.
(7) Smith, S. J.; Rittinger, K. Preparation of GTPases for structural and biophysical analysis. *Methods Mol. Biol.* **2002**, *189*, 13–24.
(8) Mondal, S.; Hsiao, K.; Goueli, S. A homogenous bioluminescent system for measuring GTPase, GTPase activating protein, and guanine nucleotide exchange factor activities. *Assay Drug Dev. Technol.* **2015**, *13*, 444–455.
(9) Schanda, P.; Kupce, E.; Brutscher, B. SOFAST-HMQC experiments for recording two-dimensional heteronuclear correlation spectra of proteins within a few seconds. *J. Biomol. NMR* **2005**, *33*, 199–211.
